# On the use of elliptic PDEs for the parameterisation of planar multipatch domains: Part 1: Numerical algorithms and foundations of parametric control

**DOI:** 10.1007/s00366-024-01997-x

**Published:** 2024-05-17

**Authors:** Jochen Hinz, Annalisa Buffa

**Affiliations:** Institute of Mathematics, École Polytechnique Fédérale de Lausanne, Rte Cantonale, 1015 Lausanne, Vaud Switzerland

**Keywords:** Parameterisation techniques, Harmonic maps, Isogeometric analysis

## Abstract

This paper presents a parameterisation framework based on (inverted) elliptic PDEs for addressing the planar parameterisation problem of finding a valid description of the domain’s interior given no more than a spline-based description of its boundary contours. The framework is geared towards isogeometric analysis (IGA) applications wherein the physical domain is comprised of more than four sides, hence requiring more than one patch. We adopt the concept of harmonic maps and propose several PDE-based problem formulations capable of finding a valid map between a convex parametric multipatch domain and the piecewise-smooth physical domain with an equal number of sides. In line with the *isoparametric paradigm* of IGA, we treat the parameterisation problem using techniques that are characteristic for the analysis step. As such, this study proposes several IGA-based numerical algorithms for the problem’s governing equations that can be effortlessly integrated into a well-developed IGA software suite. We augment the framework with mechanisms that enable controlling the parametric properties of the outcome. Parametric control is accomplished by, among other techniques, the introduction of a curvilinear coordinate system in the convex parametric domain, for which more general elliptic PDEs are adopted. Depending on the application, parametric control allows for building desired features into the computed map, such as homogeneous cell sizes or boundary layers.

## Introduction

Isogeometric analysis (IGA) [1, 2] is a variant of the finite element method (FEM) that was conceived in an effort to bridge the gap between the geometrical and the numerical aspects of the computational science and engineering (CSE) workflow. Given a physical domain ${\Omega }$, the typical CSE workflow first converts the (typically spline-based) boundary representation provided by the CAD pipeline to a simplistic representation, which is then forwarded to a classical mesh generator. However, beyond the meshing step itself, the conversion from a spline-based description of $\partial {\Omega }$ to a simplistic representation of ${\Omega }_h \approx {\Omega }$ is regarded as a major robustness bottleneck [2]. Furthermore, in many applications it is desirable to translate analysis results provided by, for instance, FEM back to appropriate changes in $\partial {\Omega }$, which may be nontrivial, due to the differing geometrical formats.

To address these concerns, IGA employs the NURBS / spline-based modelling tools that are characteristic for CAD as a basis for both the geometrical modelling and the numerical analysis aspects of the CSE workflow. In IGA, the parametric description of $\partial {\Omega }$ is immediately forwarded to a routine that solves the parameterisation problem $\partial {\Omega }\rightarrow {\Omega }$, which becomes the IGA analogue of the classical meshing step. The operator that maps an appropriately-chosen parametric domain ${\hat{{\Omega }}}$ onto ${\Omega }$ is then utilised to perform a pullback of the governing equations into ${\hat{{\Omega }}}$, where the same set of splines is employed as a basis for standard FEM techniques. Besides its promise of reducing the conversion overhead, numerical simulation based on IGA is showing promising results as spline-based FEM discretisations have been demonstrated to perform better than their classical (Lagrangian) counterparts on a range of benchmark problems [3].

The majority of existing parameterisation techniques are based on blending the (typically four) segments of $\partial {\Omega }$ into the interior [4], (constrained and unconstrained) parameterisation quality optimisation [5–9] and PDE-based approaches [10–12]. While methods from all three categories, depending on the type of the geometry, show promising results, the majority have only been studied in the singlepatch setting, i.e., when the parametric domain ${\hat{{\Omega }}}$ is given by the unit quadrilateral. For complex domains ${\Omega }$, a single quadrilateral may be too restrictive, which is why existing methods may have to be combined with segmentation algorithms that divide ${\Omega }$ into smaller pieces, which are then parameterised from the unit quadrilateral one-by-one.

To address the limitations of the singlepatch setting, this paper introduces a PDE-based parameterisation framework that is compatible with multipatch domains ${\Omega }\subset {\mathbb {R}}^2$. The idea is to introduce a multipatch covering of an appropriately-chosen convex, polygonal parametric domain ${\hat{{\Omega }}} \subset {\mathbb {R}}^2$ and to construct a nondegenerate mapping operator ${{\textbf{x}}}: {\hat{{\Omega }}} \rightarrow {\Omega }\subset {\mathbb {R}}^2$ by approximately solving a PDE problem in ${\hat{{\Omega }}}$ over a spline basis defined on the multipatch topology. The underlying PDE problem approximates a map whose inverse is comprised of a pair harmonic functions in ${\Omega }$, wherein the boundary correspondence ${{\textbf{x}}}^{-1} \vert _{\partial {\Omega }} = \partial {\hat{{\Omega }}}$ becomes the Dirichlet boundary condition. We propose two different PDE-based formulations along with various IGA-based discretisations which are then studied in detail.

A major appeal of this framework is the fact that the patch interfaces establish themselves as part of the PDE solution and need not be strongly imposed using, for instance, segmentation. The parameterisation, including the interfaces, is continuous in the boundary data and straightforwardly differentiable.

For control over the parametric properties of the computed parameterisation, we augment the framework with mechanisms that change the properties of ${{\textbf{x}}}: {\hat{{\Omega }}} \rightarrow {\Omega }$ by:

1. mapping inversely harmonically into a parametric domain with a curvilinear, instead of a Cartesian coordinate system;
2. using more general elliptic PDEs to compute a mapping operator.

As for point 1., the coordinate transformation is accomplished by the introduction of a so-called controlmap ${\textbf{s}}: {\hat{{\Omega }}} \rightarrow {\hat{{\Omega }}}$. We propose several techniques for constructing controlmaps for various desired parameterisation features, as, e.g., cell size homogenisation. As the controlmap is defined globally (i.e., over the entire parametric domain ${\hat{{\Omega }}}$), control over the parametric properties includes the image of the patch interfaces under the mapping.

This work advances upon the foundational concepts previously introduced in related studies, specifically those outlined in [10–12]. Unlike earlier approaches that often relied on ad-hoc formulations, this study introduces numerical algorithms that are grounded in more mathematically rigorous and computationally efficient principles, drawing on the thoroughly analysed concepts presented in [13–16]. Furthermore, we enhance existing methodologies by incorporating regularisations aimed at overcoming specific challenges that hinder convergence in practical applications.

We extend the parametric control concept, first introduced in [12], to multipatch configurations. This extension is accompanied by the development of novel tools designed for multipatch applications, alongside a discussion of the nuanced challenges associated with PDE-based parametric control mechanisms.

Serving as the cornerstone of this scholarly series, this work delineates the fundamental concepts, methodologies, and theoretical underpinnings that will be further explored, expanded, and applied in subsequent studies.

### Notation

This paper denotes vectors in boldface. The $i-th$ entry of a vector is denoted by $x_i$. Similarly, the *ij*-th entry of a matrix is denoted by $A_{ij}$. Let ${\textbf{y}}: {\Omega }\rightarrow {\mathbb {R}}^m$ and ${{\textbf{x}}}: {\Omega }\rightarrow {\mathbb {R}}^n$. Vectorial derivatives are taken along the second axis and we interchangeably employ the denotation

$$\begin{aligned} {\partial _{{{\textbf{x}}}} {\textbf{y}} := \frac{\partial {\textbf{y}}}{\partial {{\textbf{x}}}}} \quad \text {with} \quad \left[ \frac{\partial {\textbf{y}}}{\partial {{\textbf{x}}}} \right] _{ij} = \frac{\partial y_i}{\partial x_j}{.} \end{aligned}$$

Here, the vectorial derivative $\partial _{{{\textbf{x}}}} {\textbf{y}}$ maps into ${\mathbb {R}}^{m \times n}$, while $\nabla _{{{\textbf{x}}}} {\textbf{y}}:= (\partial _{{{\textbf{x}}}} {\textbf{y}})^T$. Furthermore, we frequently work with vector spaces ${\mathcal {V}}$. By default, we employ the abuse of notation

1 $$\begin{aligned} {\mathcal {V}}^n = \underbrace{ {\mathcal {V}} \times \cdots \times {\mathcal {V}} }_{n \text { terms}} \end{aligned}$$

and similarly for tensorial spaces, i.e., ${\mathcal {V}}^{n \times n}$. Sobolev spaces are denoted by $H^s(\Omega )$ and vectorial Sobolev spaces by $H^s(\Omega , {\mathbb {R}}^n)$. If a finite-dimensional space ${\mathcal {V}}_h$ is introduced via the span of its basis, we always denote the basis by $\{ {\mathcal {V}}_h \}$.

By ${\text {Int}}\left( D \right)$, we denote the interior of a closed domain *D*, while ${\overline{{\Omega }}}$ denotes the closure of an open domain ${\Omega }$.

### Problem statement

Let ${\Omega }\subset {\mathbb {R}}^2$ be an open, simply connected Lipschitz domain whose boundary $\partial {\Omega }$ is parameterised by an even number $K = 2n, n \in {\mathbb {N}}^{\ge 2}$ of open (spline) curves $C_k \subset {\mathbb {R}}^2$, oriented in counterclockwise direction. We consider a curve that is open in the sense of sets, meaning it does not include its endpoints. With this definition, we have

$$\begin{aligned} \partial {\Omega }= \bigcup \limits _{{k \in \{1, \ldots , K\}}} {\overline{C}}_k, \quad \text {where} \quad i \ne j \implies C_i \cap C_j = \emptyset . \end{aligned}$$

We assume that the $C_k$ are parameterised in the positive direction from the open unit interval by the spline maps ${\textbf{f}}^k: (0, 1) \rightarrow {\mathbb {R}}^2$ with ${\textbf{f}}^k \in C^1((0, 1), \, {\mathbb {R}}^2)$ and nonvanishing tangent.

Furthermore, let ${\hat{{\Omega }}} \subset {\mathbb {R}}^2$ be a convex, polygonal parametric domain with *K* sides $L_k \subset {\mathbb {R}}^2$. Each $L_k$ is given by an open (in the sense of sets) line segment and the $L_k$ are oriented in counterclockwise direction. We have

$$\begin{aligned} \partial {\hat{{\Omega }}} = \bigcup \limits _{{k \in \{1, \ldots , K\}}} {\overline{L}}_k, \quad \text {where} \quad i \ne j \implies L_i \cap L_j = \emptyset . \end{aligned}$$

Each $L_k$ is parameterised in the positive direction on $\partial {{\hat{\Omega }}}$ by an affine map ${\textbf{l}}_k: (0, 1) \rightarrow {\mathbb {R}}^2$. Denoting the corners of $\partial {{\hat{\Omega }}}$ by $\{{\varvec{\xi }}^1, \ldots , {\varvec{\xi }}^K\} \subset {\mathbb {R}}^2$, the map takes the form

$$\begin{aligned} {\textbf{l}}_k(s)= & {} {\varvec{\xi }}^k (1 - s) + {\varvec{\xi }}^{k+1} s, \quad \\{} & {} \text {where superscripts are taken modulus } K. \end{aligned}$$

Assigning the $C_k$ to the $L_k$ in ascending order induces the boundary correspondence ${\textbf{F}}: \partial {\hat{{\Omega }}} \rightarrow \partial {\Omega }$ that satisfies

2 $$\begin{aligned} {\textbf{F}} \vert _{{\overline{L}}_k} = {\overline{C}}_k, \quad \text {or equivalently} \quad {\textbf{F}} \circ {\textbf{l}}_k = {\textbf{f}}_k, \end{aligned}$$

and we assume that ${\textbf{F}}: \partial {\hat{{\Omega }}} \rightarrow \partial {\Omega }$ parameterises a Jordan curve in ${\mathbb {R}}^2$.

We assume that ${\hat{{\Omega }}}$ is covered by a quadrangulation ${\mathcal {Q}}$ of a total of $N_p$ patches ${\hat{{\Omega }}}_i$, i.e.,

3 $$\begin{aligned} {\mathcal {Q}}&= \{ {\hat{{\Omega }}}_1, \ldots , {\hat{{\Omega }}}_{N_p} \}, \quad \text {with} \quad \nonumber \\ {\hat{{\Omega }}}&= {\text {Int}} \left( \bigcup \limits _{{\hat{{\Omega }}}_i \in {\mathcal {Q}}} \overline{{\hat{{\Omega }}}}_i \right) \quad \text {and} \quad i \ne j \implies {\hat{{\Omega }}}_i \cap {\hat{{\Omega }}}_j = \emptyset . \end{aligned}$$

Each ${\hat{{\Omega }}}_i$ is the image of the reference patch ${\Omega }^{\square } = (0, 1)^2$ under the diffeomorphic bilinear map ${{\textbf{m}}}^i: {\Omega }^{\square } \rightarrow {\hat{{\Omega }}}_i$. In keeping with established practices within the numerical analysis community, the quadrangulation’s edges are regarded as open sets. The set of all edges is denoted by $\Gamma$, while boundary edges are denoted by $\Gamma ^B:= \{L_1, \ldots , L_K\}$ and interior interfaces by $\Gamma ^I:= \Gamma {\setminus } \Gamma ^B$.

For boundary patches ${\hat{{\Omega }}}_i$, i.e., patches ${{\hat{\Omega }}}_i$ with $\partial {{\hat{\Omega }}}_i \cap \partial {{\hat{\Omega }}}\ne \emptyset$, the associated map ${{\textbf{m}}}^i$ restricted to the side of $\partial {\Omega }^{\square }$ that maps onto $L_k$, is given either by ${\textbf{l}}_k(s)$ or ${\textbf{l}}_k(1 - s)$, depending on the orientation along $\partial {\hat{{\Omega }}}$. We denote the set of boundary patches by ${\mathcal {Q}}^B$.

The interface edges between pairs of neighbouring patches are denoted by $\gamma _{ij}$ and the collection of interior interfaces is given by

$$\begin{aligned} \Gamma ^I= & {} \bigcup \limits _{{(i, j) \in F^I}} \gamma _{ij}, \quad \text {with} \quad \\ F^I:= & {} \left\{ (i, j) \, \, \vert \, \, {\text {Int}} \left( \overline{{\hat{{\Omega }}}_i} \cap \overline{{\hat{{\Omega }}}}_j \right) \text { is an open line segment in } {\hat{{\Omega }}} \right\} . \end{aligned}$$

Given ${{\hat{\Omega }}}$ and a suitable multipatch covering, we denote by $\varvec{\Xi }_i = (\Xi _{i, 1}, \Xi _{i, 2})$ the pair of local (open) knotvectors associated with the *i*-th patch along with the associated canonical spline basis $\{ \widehat{{\mathcal {V}}}_{h, i} \} \subset H^2({\Omega }^{\square })$. We denote by $\{ {\mathcal {V}}_h^{\text {disc}} \} \subset L^2({{\hat{\Omega }}})$ the basis that results from a push-forward of the local bases $\{ \widehat{{\mathcal {V}}}_{h, i} \}$, i.e,

$$\begin{aligned} \left\{ {\mathcal {V}}_h^{\text {disc}} \right\} = \bigcup \limits _{i \in \{1, \ldots , N_p \}} \left\{ v_h \circ ({\textbf{m}}^i)^{-1} \, \, \vert \, \, v_h \in \{ \widehat{{\mathcal {V}}}_{h, i} \} \right\} . \end{aligned}$$

Then, we define the space ${\mathcal {V}}_h:= {\text {span}}\,\{{\mathcal {V}}_h^{\text {disc}}\} \cap C^0({{\hat{\Omega }}})$ whose canonical basis $\{ {\mathcal {V}}_h \}$ follows from coupling the degrees of freedom of $\{{\mathcal {V}}_h^{\text {disc}} \}$ that are incident on the $\gamma _{ij} \in \Gamma ^I$ in the usual way. We assume that the knotvector pairs $\varvec{\Xi }_i$ result in a basis $\{ {\mathcal {V}}_h \}$ which forms a partition of unity on ${{\hat{\Omega }}}$ that is compatible with ${\textbf{F}}: \partial {{\hat{\Omega }}}\rightarrow \partial {\Omega }$ in the sense that the set

4 $$\begin{aligned} {\mathcal {U}}^{{\textbf{F}}}_h := \{ {\textbf{v}} \in {\mathcal {V}}_h^2 \, \, \vert \, \, {\textbf{v}} = {\textbf{F}} \text { on } \partial {{\hat{\Omega }}}\} \end{aligned}$$

is nonempty (i.e., the boundary correspondence is an element of the trace space of ${\mathcal {V}}_h^2$).

Having established the necessary conceptual groundwork, the purpose of this paper is providing a framework for finding a nondegenerate mapping operator ${{\textbf{x}}}_h: {\hat{{\Omega }}} \rightarrow {\Omega }$ with ${{\textbf{x}}}_h \in {\mathcal {U}}^{{\textbf{F}}}_h$. Since this paper’s methodology is based on variational formulations of elliptic PDEs, we adopt a notion of nondegeneracy in the sense of distributions. Denoting the Cartesian coordinate functions in ${\hat{{\Omega }}}$ by ${\varvec{\xi }}= (\xi _1, \xi _2)^T$, we call a map ${{\textbf{x}}}: {{\hat{\Omega }}}\rightarrow {\Omega }$ *nondegenerate* (NDG) if

5 $$\begin{aligned} { \det J({{\textbf{x}}}) \ge 0, \quad \text {almost everywhere in } {{\hat{\Omega }}}, } \end{aligned}$$

where $J({{\textbf{x}}}):= \partial _{{\varvec{\xi }}} {{\textbf{x}}}$ denotes the Jacobian matrix of ${\textbf{x}}: {\hat{{\Omega }}} \rightarrow {\Omega }$ in ${\hat{{\Omega }}}$. Similarly, we call a map *uniformly nondegenerate* (UNDG) if there are constants $0< c_1 \le c_2 < \infty$ such that

6 $$\begin{aligned} { c_1 \le \det J({{\textbf{x}}}) \le c_2, \quad \text {a.e. in } {{\hat{\Omega }}}. } \end{aligned}$$

Clearly, uniform nondegeneracy of ${{\textbf{x}}}_h \in {\mathcal {V}}_h^2$ is favoured over nondegeneracy by most applications but imposes stronger requirements on ${\textbf{F}}: \partial {{\hat{\Omega }}}\rightarrow \partial {\Omega }$ that are discussed in Sect. 3.3.

Besides nondegeneracy, this paper aims for mechanisms that allow for control over the parametric properties of ${{\textbf{x}}}_h: {\hat{{\Omega }}} \rightarrow {\Omega }$ while the framework should be implicitly differentiable, i.e., provide maps that are a continuous function of the supplied data, namely the boundary correspondence ${\textbf{F}}: \partial {{\hat{\Omega }}}\rightarrow \partial {\Omega }$.

### Related work

Isogeometric analysis has made possible the seamless integration of geometric modeling tools with numerical analysis. As such, it has seen interdisciplinary efforts of addressing the parameterisation problem, such as interpolation-based methods, generalisations of classical meshing algorithms to higher-order spline functions and methods originating from computer graphics. Examples of interpolation-based (IB) approaches are the bilinearly blended Coons’ patch approach [4] and Hermite blending [17, Chapter 5] for four-sided domains and their generalisations to general (convex) polygonal domains [18, 19]. Most IB approaches had already been conceived before the onset of IGA. While computationally inexpensive and often highly effective in practical applications, IB methods provide no guarantee of nondegeneracy and the resulting maps are therefore often folded.

Generalisations of classical (structured) meshing techniques to higher-order spline functions mainly rely on optimisation, both without [8, 9, 20] and with added constraints [5, 7]. Here, the higher-order nature of spline basis functions allows for quality criteria based on higher-order derivative information. As such, optimisation-based techniques have received more interest within the IGA-realm compared to their classical counterparts. Optimisation is usually nonconvex and faces the risk of converging to a local minimum or to a folded map in the unconstrained case. Optimising the map under an added constraint that is a sufficient condition for $\det J({{\textbf{x}}}_h) > 0$ in ${{\hat{\Omega }}}$, on the other hand, drastically increases computational complexity and furthermore requires finding a feasible initial iterate first. To overcome this shortcoming, penalty-based methods have been proposed [21, 22]. Optimisation-based approaches, in particular penalty-based and unconstrained, are straightforwardly generalised to the multipatch setting wherein the interface control points become degrees of freedom in the formulation. In [23], for instance, *patch adjacency graphs* are employed to study multipatch parameterisation based on optimisation.

Methods for multipatch region parameterisation that originate from computer graphics mainly rely on frame / cross field approaches [24, 25]. Cross field (CF) based approaches segment the physical domain into regions that are diffeomorphic to the unit quadrilateral, which are then parameterised one-by-one using a singlepatch parameterisation algorithm. In the IGA realm, besides multipatch parameterisation, this concept has seen applications to NURBS untrimming [26, 27]. CF-based approaches require an initial, valid parameterisation of the geometry, for instance a triangulation. While CF techniques offer limited control over the parametric properties across patch interfaces, they are suitable for a broad spectrum of geometric shapes.

Finally, the IGA community has seen efforts to generalise classical, PDE-based methods to higher-order splines via variational formulations [10, 12] or by leveraging the boundary element method [8]. A PDE-based method aimed at finding the inverse of a harmonic map between $\Omega$ and some convex multipatch domain ${{\hat{\Omega }}}$ is studied in [11]. The paper proposes an ad-hoc discretisation of a variational formulation of the *Elliptic Grid Generation* (EGG) equations. Building upon the groundwork established by [11], this paper presents algorithms that enhance both the mathematical rigor and the computational efficiency of the IGA treatment of the variational EGG equations.

## Theory

This paper proposes a framework for computing parameterisations ${{\textbf{x}}}_h: {{\hat{\Omega }}}\rightarrow {\Omega }$ based on harmonic maps. In the following, we present an in-depth discourse on harmonic maps as well as finite element techniques for elliptic equations in nonvariational form, which shall be adopted to formulate discretisations in Sect. 3.1.

### Harmonic maps

The motivation to seek the map ${{\textbf{x}}}_h: {{\hat{\Omega }}}\rightarrow {\Omega }$ as the inverse of a map that is harmonic in ${\Omega }$ stems from the following famous result:

#### Theorem 1

(Radó-Kneser-Choquet) The harmonic extension of a homeomorphism from the boundary of a Jordan domain ${\Omega }\subset {\mathbb {R}}^2$ onto the boundary of a convex domain ${{\hat{\Omega }}}\subset {\mathbb {R}}^2$ is a diffeomorphism in ${\Omega }$.

For proofs, we refer to [28–31]. It should be noted that the convexity of ${{\hat{\Omega }}}$ is a sufficient, but not a necessary condition. Furthermore, the same result is no longer true in ${\mathbb {R}}^3$ [32].

Theorem 1 has inspired many numerical approaches for finding a nondegenerate ${{\textbf{x}}}_h: {{\hat{\Omega }}}\rightarrow {\Omega }$ that approximates a map whose inverse is harmonic. Besides the nondegeneracy guarantee, this is furthermore explained by the regularity of harmonic maps which generally serve the map’s quality from a numerical standpoint.

Numerical approaches go back to the pioneering works of Winslow [33]. Letting ${{\textbf{x}}}= (x_1, x_2)^T$, and defining the metric tensor

$$\begin{aligned} G_{ij}({{\textbf{x}}}) = g_{ij} \quad \text {with} \quad g_{ij} = \partial _{\xi _i} {{\textbf{x}}}\cdot \partial _{\xi _j} {{\textbf{x}}}, \end{aligned}$$

Winslow’s original approach seeks the map ${{\textbf{x}}}: {{\hat{\Omega }}}\rightarrow {\Omega }$ as the result of the following minimisation problem

7 $$\begin{aligned} \frac{1}{2} \int \limits _{{\Omega }}{\text {tr}}\left( G^{-1} \right) \textrm{d} {{\textbf{x}}}\rightarrow \min \limits _{{{\textbf{x}}}}, \quad \text {s.t.} \quad {\varvec{\xi }}({{\textbf{x}}}) = {\textbf{F}}^{-1} \text { on } \partial {\Omega }. \end{aligned}$$

Letting

8 $$\begin{aligned} {\mathcal {U}}^{{\textbf{F}}} := \{ {\textbf{v}} \in H^1({{\hat{\Omega }}}, {\mathbb {R}}^2) \,\, \vert \,\, {\textbf{v}} = {\textbf{F}} \text {on} \partial {{\hat{\Omega }}}\}, \end{aligned}$$

a pullback leads to

9 $$\begin{aligned} C_W({\textbf{x}}) := \frac{1}{2} \int \limits _{{{\hat{\Omega }}}} \frac{{\text {tr}}(G)}{\det J} \textrm{d} {\varvec{\xi }}\rightarrow \min \limits _{{{\textbf{x}}}\in {\mathcal {U}}^{\textbf{F}}}{.} \end{aligned}$$

The associated optimality condition follows from taking Gateaux derivatives, i.e.,

10 $$\begin{aligned} \text {for optimal } {\textbf{x}}^{*}: \quad \left. \frac{\partial }{\partial \varepsilon } C_W \left( {\textbf{x}}^{*} + \varepsilon \varvec{\phi } \right) \right| _{\varepsilon = 0} = 0, \quad \forall \varvec{\phi } \in {\mathcal {U}}^{{\textbf{0}}}. \end{aligned}$$

A discretisation replaces ${\mathcal {U}}^{{\textbf{F}}} \rightarrow {\mathcal {U}}^{{\textbf{F}}}_h$ in (9). The minimisation of (9) is highly impractical since the domain of the integrand is the set of all ${{\textbf{x}}}\in {\mathcal {U}}^{{\textbf{F}}}$ that satisfy $\det J({{\textbf{x}}}) > 0$ (almost everywhere). As such, minimisation has to be initialised with a nondegenerate initial map which is generally hard to find.

An alternative formulation is based on the harmonicity requirement’s *classical form*:

11 $$\begin{aligned} \Delta {{\textbf{x}}}^{-1} = 0 \quad \text { in } {\Omega }, \quad \text {s.t.} \quad {{\textbf{x}}}^{-1} = {\textbf{F}}^{-1} \text { on } \partial {\Omega }, \end{aligned}$$

where the Laplace operator is to be understood component-wise. A pullback leads to

12 $$\begin{aligned} \Delta _{{{\textbf{x}}}} {\varvec{\xi }}= 0 \quad \text {in } {{\hat{\Omega }}}, \quad \text {s.t.} \quad {{\textbf{x}}}= {\textbf{F}} \text { on } \partial {{\hat{\Omega }}}, \end{aligned}$$

where $\Delta _{{\textbf{x}}}$ denotes the Laplace-Beltrami operator.

The pullbacks from both (9) and (12) inherently assume that ${{\textbf{x}}}^{-1}: {\Omega }\rightarrow {{\hat{\Omega }}}$ is invertible, thus potentially rendering the problems ill-posed in case ${{\hat{\Omega }}}$ is not convex. However, assuming convexity of ${{\hat{\Omega }}}$, we may multiply the two-component PDE from (12) by $T: {{\hat{\Omega }}}\rightarrow {\mathbb {R}}^{2 \times 2}$, with $T = \left( \det J \right) ^2 J({{\textbf{x}}})$ since *T* does not vanish in the interior. The result is a two-component PDE for ${{\textbf{x}}}: {{\hat{\Omega }}}\rightarrow {\Omega }$ which can be classified as a quasilinear second-order elliptic PDE in nondivergence form [10, 12, 34]:

13 $$\begin{aligned} i \in \{1, 2\}: \quad A(\partial _{{\varvec{\xi }}} {{\textbf{x}}}) :H(x_i) = 0, \quad \text {s.t.} \quad {{\textbf{x}}}= {\textbf{F}} \text { on } \partial {{\hat{\Omega }}}, \end{aligned}$$

where

$$\begin{aligned} H(y)_{ij}= & {} \frac{\partial ^2 y}{\partial \xi _i \partial \xi _j} \quad \text {denotes the Hessian in } {{\hat{\Omega }}}, \quad \text {while} \quad \\ A(\partial _{{\varvec{\xi }}} {{\textbf{x}}}):= & {} \begin{pmatrix} g_{22} &{} -g_{12} \\ -g_{12} &{} g_{11} \end{pmatrix} \end{aligned}$$

and $A :B$ denotes the Frobenius inner product between two matrices.

The multiplication by $T: {{\hat{\Omega }}}\rightarrow {\mathbb {R}}^{2 \times 2}$ removes $\det J$ from the original formulation’s denominator, allowing schemes based on (13) to be initialised with degenerate initial maps. On the other hand, minimisation based on (9) most likely yields a nondegenerate map, while this may not hold for approaches based on (13), due to the scheme’s truncation error.

A further possibility is basing the scheme on the harmonicity requirement’s *weak form*. More precisely, with ${\mathcal {V}} = H^1({\Omega })$ and $\mathring{{\mathcal {V}}}:= H^1_0(\Omega )$:

14 $$\begin{aligned}&\text {find } {{\textbf{x}}}^{-1} \in {\mathcal {V}}^2, \quad \text {s.t.} \quad \int \limits _{{\Omega }} \nabla \varvec{\phi } :\nabla {{\textbf{x}}}^{-1} \textrm{d} {{\textbf{x}}}= 0, \quad \nonumber \\&\quad \forall \varvec{\phi } \in \mathring{{\mathcal {V}}}^2 \quad \text {and} \quad {{\textbf{x}}}^{-1} = {\textbf{F}}^{-1} \text { on } \partial {\Omega }, \end{aligned}$$

which translates to an equation for ${{\textbf{x}}}: {{\hat{\Omega }}}\rightarrow {\Omega }$ via a pullback.

The relationship between the formulation based on (9) and (14) is that the latter reverses the order of operations, i.e., it takes the Gateaux derivative of (7) *before* performing the pullback from $\Omega$ into ${\hat{\Omega }}$. As such, the test functions $\varvec{\phi } \in \mathring{{\mathcal {V}}}^2$ are defined in $\Omega$ rather than in ${\hat{\Omega }}$.

This paper presents algorithms for approximating ${{\textbf{x}}}_h \approx {{\textbf{x}}}$ based on formulations (13) and (14). As the former is in nondivergence form, in the following we give a brief summary on the finite element treatment of nondivergence form equations.

### Nondivergence form equations

The finite element treatment of nondivergence form (NDF) equations is a relatively recent development with first contributions due to Lakkis and Pryer [13]. NDF-equations are of the form

15 $$\begin{aligned}&B :H(u) + \text { lower order terms } \nonumber \\&\quad = f \quad \text {a.e. in } {{\hat{\Omega }}}, \quad \text {s.t.} \quad u = g \text { on } \partial {{\hat{\Omega }}}. \end{aligned}$$

Here, $f \in L^2({{\hat{\Omega }}})$, while $B: {{\hat{\Omega }}}\rightarrow L^\infty ({{\hat{\Omega }}}, {\mathbb {R}}^{2 \times 2})$ is uniformly elliptic, i.e., there are constants $0< c_1 \le c_2 < \infty$ such that

16 $$\begin{aligned} c_1 \le \inf \limits _{{\varvec{\xi }}\in {\mathbb {R}}^2, \Vert {\varvec{\xi }}\Vert = 1} {\varvec{\xi }}^T B {\varvec{\xi }}\le \sup \limits _{{\varvec{\xi }}\in {\mathbb {R}}^2, \Vert {\varvec{\xi }}\Vert = 1} {\varvec{\xi }}^T B {\varvec{\xi }}\le c_2, \quad \text { a.e. in } {{\hat{\Omega }}}. \end{aligned}$$

The set of all symmetric and uniformly elliptic $B: {{\hat{\Omega }}}\rightarrow L^\infty ({{\hat{\Omega }}}, {\mathbb {R}}^{2 \times 2})$ is referred to as $\text {SPD}^{2 \times 2}({{\hat{\Omega }}})$. In the following, we take $g = 0$ and disregard lower order terms for convenience.

For $g = 0$ and ${{\hat{\Omega }}}\subset {\mathbb {R}}^2$ convex, it can be shown that $u \in H^2({{\hat{\Omega }}}) \cap H^1_0({{\hat{\Omega }}})$ as long as *B* satisfies the so-called Cordés condition [35]. In ${\mathbb {R}}^2$ the Cordés condition is implied by (16). Defining

17 $$\begin{aligned} \gamma (B) := {\text {tr}}(B) / \Vert B \Vert _F^2, \end{aligned}$$

where $\Vert \, \cdot \, \Vert _F$ denotes the Frobenius norm $\sqrt{A :A}$ of a matrix, finite element discretisations are based on the following Petrov-Galerkin formulation of the problem’s *strong form*:

18 $$\begin{aligned}&\text {find } u \in H^2({{\hat{\Omega }}}) \cap H^1_0({{\hat{\Omega }}}) \quad \text {s.t.} \quad \nonumber \\&\quad \int \limits _{{{\hat{\Omega }}}} \gamma (B) \tau (\phi ) \left( B :H(u) - f \right) \textrm{d} {\varvec{\xi }}= 0, \quad \forall \phi \in {\mathring{{\mathcal {V}}}}, \end{aligned}$$

for some suitably-chosen test space $\mathring{{\mathcal {V}}}$.

Here, $\tau : \mathring{{\mathcal {V}}} \rightarrow L^2({{\hat{\Omega }}})$ is a suitably-chosen operator that warrants coercivity of the associated bilinear form over finite-dimensional subspaces $\mathring{{\mathcal {V}}}_h \subset \mathring{{\mathcal {V}}}$. The (optional) scaling $\gamma (\, \cdot \,)$ guarantees that $\gamma (B) \, B$ resembles the identity matrix ${\mathcal {I}}^{2 \times 2}$ and simplifies the analysis of numerical schemes based on (18). The choices of $\tau : \mathring{{\mathcal {V}}} \rightarrow L^2({{\hat{\Omega }}})$ for $\mathring{{\mathcal {V}}} = H^2({{\hat{\Omega }}}) \cap H^1_0({{\hat{\Omega }}})$ are $\tau _{\text {NS}}(v) = \Delta v$ and $\tau _{\text {LS}}(v) = B :H(v)$ [14, 15, 36], while for $\mathring{{\mathcal {V}}} = H^1_0({{\hat{\Omega }}})$, $\tau _{\text {ID}}(v) = v$ [13, 37]. To enable discretisations over finite element spaces $\mathring{{\mathcal {V}}}_h \subset H^1_0({{\hat{\Omega }}})$, mixed-FEM formulations of (18) are introduced in [13, 14] while [15, 36] propose $C^0$ discontinuous Galerkin schemes that introduce interior penalty terms over the interfaces of the FEM mesh, acting on the discrete solution’s normal gradient.

As spaces ${\mathcal {V}}_h$ resulting from local spline-based constructions over multipatch topologies are generally only in $H^1({{\hat{\Omega }}})$, this paper adopts the mixed formulations based on Gallistl [14] and Lakkis-Pryer [13] as well as the $C^0$-DG formulation from [15] and applies them to linearisations of (13). In the case of $C^0$-DG, penalty terms can be restricted to the interior patch interfaces $\gamma _{ij} \in \Gamma ^I$ of ${{\hat{\Omega }}}$.

## Numerical schemes

In this section we propose several numerical schemes for finding approximate solutions of the inverse harmonicity formulations based on both (13) and (14). For this purpose, we recall the definitions of ${\mathcal {U}}^{{\textbf{F}}}$ and its discrete counterpart ${\mathcal {U}}_h^{{\textbf{F}}}$ from equations (8) and (4), respectively. The associated zero trace spaces are denoted by ${\mathcal {U}}^{{\textbf{0}}}$ and ${\mathcal {U}}^{{\textbf{0}}}_h$, respectively.

### NDF discretisations

The discretisations of (13) are based on a variation of the Petrov-Galerkin formulation from (18). Here, we restrict ourselves to the choices $\tau \in \{\tau _{\text {NS}}, \tau _{ID} \}$. In the following, we propose iterative solution strategies targeting a variational form of (13). For the sake of a unified presentation, we introduce ${\mathcal {U}}^{{\textbf{F}}, 2}:= {\mathcal {U}}^{{\textbf{F}}} \cap H^2({{\hat{\Omega }}}, {\mathbb {R}}^2)$, with ${\mathcal {U}}^{\textbf{F}}$ as defined in Sect. 2.1, in addition to the form ${\mathcal {L}}: \text {SPD}^{2 \times 2}({{\hat{\Omega }}}) \times {\mathcal {U}}^{{\textbf{F}}, 2} \times {\mathcal {U}}_{\text {test}} \rightarrow {\mathbb {R}}$ with

19 $$\begin{aligned} {\mathcal {L}}(B, {{\textbf{x}}}, \varvec{\phi }) := \int \limits _{{{\hat{\Omega }}}} \tau (\phi _i) B \, :\, H(x_i) \, \textrm{d} {\varvec{\xi }}, \end{aligned}$$

where we sum over repeated indices. Note that for ${\mathcal {U}}^{{\textbf{F}}, 2}$ to be nonempty, ${\textbf{F}}: \partial {{\hat{\Omega }}}\rightarrow \partial {\Omega }$ needs to be the trace of a pair of $H^2({{\hat{\Omega }}})$ functions. Hence, for the time being, we assume that the data is sufficiently regular for the problem and its linearisations to be well-posed over ${\mathcal {U}}^{{\textbf{F}},2}$ for the test spaces ${\mathcal {U}}_{\text {test}} = H^1_0({{\hat{\Omega }}}, {\mathbb {R}}^2)$ ($\tau = \tau _{\text {ID}}$) and ${\mathcal {U}}_{\text {test}} = {\mathcal {U}}^{{\textbf{0}}, 2}$ ($\tau = \tau _{\text {NS}}$). The linearisations are then modified for compatibility with the $C^0({{\hat{\Omega }}})$-nature of spline spaces over multipatch topologies by introducing mechanisms that relax the associated operator’s regularity requirements from the set ${\mathcal {U}}^{{\textbf{F}}, 2}$ back to ${\mathcal {U}}^{{\textbf{F}}}$. Meanwhile, discretisations follow readily from replacing vector spaces by their finite-dimensional counterparts.

In what follows, we shall substitute various flavours of $A(\, \cdot \,)$ (cf. Sect. 2.1) scaled by $\gamma (\, \cdot \,)$ (cf. Sect. 2.2) for the *B* argument of ${\mathcal {L}}(B, \, \cdot \,, \, \cdot \,)$ introduced in  (19). Besides being customary in NDF-discretisations, we have noticed the scaling via $\gamma ( \, \cdot \,)$ to have a positive effect on the iterative schemes’ radii of convergence and the required number of iterations. Furthermore, to warrant scaling invariance of the stabilisation / regularisation parameters encountered in the numerical schemes, we assume the coordinate system to gauge the geometry to unit surface area. Should scaling invariance necessitate a coordinate transformation, it may be reversed upon finalisation of the numerical algorithm. This is a reasonable strategy since in the absence of stabilisation / regularisation parameters, the associated operations commute.

#### Fixed-point iteration

The most elementary linearisation is based on a fixed-point iteration which freezes $A( \, \cdot \,)$ of (13) in the previous iterate ${{\textbf{x}}}^k$ and seeks ${{\textbf{x}}}: {{\hat{\Omega }}}\rightarrow {\Omega }$ as the limit $k \rightarrow \infty$ of the recursion

20 $$\begin{aligned} i \in \{1, 2\}: \quad A(\partial _{{\varvec{\xi }}} {{\textbf{x}}}^k) \, :\, H(x^{k+1}_i) = 0, \quad \text {s.t.} \quad {{\textbf{x}}}^{k+1} = {\textbf{F}} \text { on } {{\hat{\Omega }}}. \end{aligned}$$

We note that $A(\partial _{{\varvec{\xi }}} {{\textbf{x}}})$ may equivalently be written in the form

21 $$\begin{aligned} A(\partial _{{\varvec{\xi }}} {{\textbf{x}}}) = C^T C, \quad \text {with} \quad C(\partial _{{\varvec{\xi }}} {{\textbf{x}}}) = \begin{pmatrix} \tfrac{\partial x_2}{\partial \xi _2} &{} -\tfrac{\partial x_2}{\partial \xi _1} \\ - \tfrac{\partial x_1}{\partial \xi _2} &{} \tfrac{\partial x_1}{\partial \xi _1} \end{pmatrix}. \end{aligned}$$

Since $C(\, \cdot \,)$ has the same characteristic polynomial as $J = \partial _{{\varvec{\xi }}} {{\textbf{x}}}$, we conclude that $A(\, \cdot \,) \in \text {SPD}^{2 \times 2}({{\hat{\Omega }}})$ whenever ${{\textbf{x}}}: {{\hat{\Omega }}}\rightarrow {\mathbb {R}}^2$ is UNDG. As such, the uniform ellipticity requirement (16) is violated for degenerate intermediate maps. To circumvent this issue, we introduce the stabilisation $A_\mu (\, \cdot \,):= A( \, \cdot \,) + \mu {\mathcal {I}}^{2 \times 2}$, with $0< \mu < 1$ and base a numerical scheme on the following linearised classical form:

22 $$\begin{aligned} i&\in \{1, 2\}: \quad A_{\mu }(\partial _{{\varvec{\xi }}} {{\textbf{x}}}^k) \, :\, H(x^{k+1}_i) \nonumber \\&\quad - \mu \Delta x^k_i = 0, \quad \text {s.t.} \quad {{\textbf{x}}}^{k+1} = {\textbf{F}} \text { on } {{\hat{\Omega }}}. \end{aligned}$$

With $A_{\mu }^k:= A_{\mu }(\partial _{{\varvec{\xi }}} {{\textbf{x}}}^k)$ and $\gamma _\mu ^k:= \gamma (A^k_\mu )$ (cf. equation (17)), a variational formulation seeks ${{\textbf{x}}}: {{\hat{\Omega }}}\rightarrow {\Omega }$ as the limit $k \rightarrow \infty$ of the recursion

23 $$\begin{aligned} \text {find } {{\textbf{x}}}^{k+1} \in {{\mathcal {U}}^{{\textbf{F}}, 2}} \quad \text {s.t.} \quad {\mathcal {F}}_{\mu }( {{\textbf{x}}}^{k+1}, {{\textbf{x}}}^{k}, \varvec{\phi }) = 0, \quad \forall \varvec{\phi } \in {{\mathcal {U}}^{{\textbf{0}}, 2}}, \end{aligned}$$

where

24 $$\begin{aligned} {\mathcal {F}}_{\mu }({{\textbf{x}}}^{k+1}, {{\textbf{x}}}^k, \varvec{\phi })&:= {\mathcal {L}}(\gamma _\mu ^k \, A_\mu ^k, {{\textbf{x}}}^{k+1}, \varvec{\phi }) \nonumber \\&\quad - \mu {\mathcal {L}}(\gamma _\mu ^k \, {\mathcal {I}}^{2 \times 2}, {{\textbf{x}}}^{k}, \varvec{\phi }). \end{aligned}$$

In practice, we take $\mu = 10^{-4}$. Here, a reasonable stopping criterion terminates the recursion as soon as $\Vert {{\textbf{x}}}^{k+1} - {{\textbf{x}}}^{k} \Vert / \Vert {{\textbf{x}}}^k \Vert < \varepsilon$, where a suitable norm depends on the augmented scheme (with $C^0$-support).

#### Newton approach

As in the fixed-point iteration, a linearisation based on Newton’s method needs to be adjusted for the possibility of encountering iterates ${{\textbf{x}}}^{k}$ with $A(\partial _{{\varvec{\xi }}} {{\textbf{x}}}^{k}) \notin \text {SPD}^{2 \times 2}$. As such, we again employ the eigenspectrum shift $A( \, \cdot \,) \rightarrow A_{\mu }(\, \cdot \,)$ and base a Newton scheme on the residual form ${\mathcal {N}}_{\mu }: {{\mathcal {U}}^{{\textbf{F}}, 2}} \times {{\mathcal {U}}^{{\textbf{0}}, 2}} \rightarrow {\mathbb {R}}$, with

25 $$\begin{aligned} {\mathcal {N}}_{\mu }({{\textbf{x}}}, \varvec{\phi })&:= {\mathcal {L}}(\gamma _{\mu }^{{{\textbf{x}}}} A_{\mu }^{{{\textbf{x}}}}, {{\textbf{x}}}, \varvec{\phi }), \quad \text {where} \quad \nonumber \\ A_{\mu }^{{{\textbf{x}}}}&:= A_{\mu }(\partial _{{\varvec{\xi }}} {{\textbf{x}}}) \quad \text { and } \quad \gamma _{\mu }^{{{\textbf{x}}}} := \gamma (A_\mu ^{{{\textbf{x}}}}). \end{aligned}$$

Given some intermediate iterate ${{\textbf{x}}}^{k} \in {\mathcal {U}}^{{\textbf{F}}, 2}$, the Newton scheme computes the increment $\partial {{\textbf{x}}}^k \in {\mathcal {U}}^{{\textbf{0}}, 2}$ from:

26 $$\begin{aligned}&\text {find } \partial {{\textbf{x}}}^k \in {{\mathcal {U}}^{{\textbf{0}}, 2}}, \quad \text {s.t.} \quad {\mathcal {N}}_{\mu }^{\prime }({{\textbf{x}}}^k, \varvec{\phi }, \partial {{\textbf{x}}}^k) \nonumber \\&\quad = - {\mathcal {N}}_{\mu }({{\textbf{x}}}^k, \varvec{\phi }), \quad \forall \varvec{\phi } \in {{\mathcal {U}}^{{\textbf{0}}, 2}}, \end{aligned}$$

wherein ${\mathcal {N}}_{\mu }^{\prime }(\, \cdot \,, \, \cdot \,, {\textbf{v}})$ denotes the Gateaux derivative of ${\mathcal {N}}_{\mu }(\, \cdot , \, \cdot \,)$ with respect to its first argument in the direction of ${\textbf{v}} \in {{\mathcal {U}}^{{\textbf{0}}, 2}}$. The new iterate becomes ${{\textbf{x}}}^{k+1} = {{\textbf{x}}}^k + \kappa \partial {{\textbf{x}}}^k$, where the optimal value of $\kappa \in (0, 1]$ is estimated using a line search routine.

Contrary to the fixed-point iteration, for $\mu > 0$ the root of ${\mathcal {N}}_{\mu }(\, \cdot , \, \cdot \,)$ generally differs from that of $\mu = 0$. As such, the eigenspectrum shift constitutes a regularisation rather than a stabilisation. Therefore, $\mu$ needs to be taken small and we utilise $\mu = 10^{-5}$ in practice. While in discretisations based on (26), the value of $\mu$ can be reduced to $\mu = 0$ in an outer loop, in practice this is usually not necessary. In fact, schemes based on (26) converge in the vast majority of cases even for $\mu = 0$ and the stabilisation with $\mu > 0$ merely improves convergence behaviour for severely folded initial iterates.

#### Hessian recovery approach

Having discussed the linearisations of the continuous variational formulation of (13), we can proceed to concrete discretisations. Clearly, for bases ${\mathcal {V}}_h \subset H^2({{\hat{\Omega }}})$, discretisations follow from replacing ${\mathcal {U}}^{{\textbf{F}}, 2}$ by its discrete counterpart ${\mathcal {U}}_h^{{\textbf{F}}, 2}$. For compatibility with spaces ${\mathcal {V}}_h \subset H^1({{\hat{\Omega }}})$, in the following, we extend the linearisations from Sects. 3.1.1 and 3.1.2 with the weak Hessian recovery approach proposed in [13]. In what follows, we assume that $\tau (\, \cdot \, ) = \tau _{\text {ID}}(\, \cdot \,)$.

Assuming sufficient regularity of $u: {{\hat{\Omega }}}\rightarrow {\mathbb {R}}$ and $\Phi : {{\hat{\Omega }}}\rightarrow {\mathbb {R}}^{2 \times 2}({{\hat{\Omega }}})$, the Hessian recovery approach is based on the following integration by parts formula:

$$\begin{aligned} \int \limits _{{{\hat{\Omega }}}} H(u) \, :\, \Phi \, \textrm{d} {\varvec{\xi }}= - \int \limits _{{{\hat{\Omega }}}} \nabla u \, \cdot \, \left( \nabla \cdot \Phi \right) \textrm{d} {\varvec{\xi }}+ \int \limits _{\partial {{\hat{\Omega }}}} \nabla u \cdot \left( \Phi {\textbf{n}} \right) \textrm{d} \Gamma , \end{aligned}$$

wherein ${\textbf{n}}: \partial {{\hat{\Omega }}}\rightarrow {\mathbb {R}}^2$ denotes the outward normal vector on $\partial {{\hat{\Omega }}}$, while the divergence $\nabla \cdot (\, \cdot \,)$ applied to $\Phi : {{\hat{\Omega }}}\rightarrow {\mathbb {R}}^{2 \times 2}$ is taken row-wise. This section’s approach allows us to relax the regularity requirements compared to Sect. 3.1. As such, we seek a solution from the set ${\mathcal {U}}^{{\textbf{F}}}$ rather than ${\mathcal {U}}^{{\textbf{F}},2}$. Furthermore, we introduce ${\mathcal {W}}:= H^1({{\hat{\Omega }}}, {\mathbb {R}}^{2 \times 2})$ as well as $X:= ({{\textbf{x}}}, {\widehat{H}}) \in {\mathcal {U}}^{{\textbf{F}}} \times {\mathcal {W}}^2$ and $\Sigma := (\varvec{\phi }, \Phi ) \in {\mathcal {U}}^{{\textbf{0}}} \times {\mathcal {W}}^2$. Analogous to (19), we base a numerical scheme on the form ${\mathcal {L}}^H: \text {SPD}^{2 \times 2} \times \left( {\mathcal {U}}^{{\textbf{F}}} \times {\mathcal {W}}^2 \right) \times \left( {\mathcal {U}}^{{\textbf{0}}} \times {\mathcal {W}}^2 \right)$, with

27 $$\begin{aligned} {\mathcal {L}}^H(B, X, \Sigma )&= \int \limits _{{{\hat{\Omega }}}} \phi _i B \, :\, {\widehat{H}}_i \, \textrm{d} {\varvec{\xi }}\nonumber \\&\quad + \int \limits _{{{\hat{\Omega }}}} \left( {\widehat{H}}_i \, :\, \Phi _i + \nabla x_i \, \cdot \, \left( \nabla \cdot \Phi _i \right) \right) \textrm{d} {\varvec{\xi }}\nonumber \\&\quad - \int \limits _{\partial {{\hat{\Omega }}}} \nabla x_i \cdot (\Phi _i {\textbf{n}}) \, \textrm{d} \Gamma , \end{aligned}$$

wherein we sum over repeated indices. Note that here, elements $Q \in {\mathcal {W}}^2$ are of the form $Q = (Q_1, Q_2) \in {\mathcal {W}} \times {\mathcal {W}}$ (and are therefore indexed in the same way as vectors). Letting, again, $A_\mu ^k:= A(\partial _{{\varvec{\xi }}} {{\textbf{x}}}^k)$, the fixed-point iteration is based on

28 $$\begin{aligned}&\text {find } X^{k+1} \in {\mathcal {U}}^{{\textbf{F}}} \times {\mathcal {W}}^2 \quad \text {s.t.} \quad {\mathcal {F}}^H_{\mu }(X^{k+1}, X^k, \Sigma ) = 0 \quad \nonumber \\&\quad \forall \Sigma \in {\mathcal {U}}^{{\textbf{0}}} \times {\mathcal {W}}^2, \end{aligned}$$

where

29 $$\begin{aligned} {\mathcal {F}}^H_{\mu }(X^{k+1}, X^k, \Sigma )&:= {\mathcal {L}}^H(\gamma ^k_\mu A_\mu ^k, X^{k+1}, \Sigma ) \nonumber \\&\quad - \mu {\mathcal {L}}^H(\gamma ^k_\mu {\mathcal {I}}^{2 \times 2}, X^{k}, \Sigma ). \end{aligned}$$

Following [13], a discretisation replaces ${\mathcal {U}}^{{\textbf{F}}} \rightarrow {\mathcal {U}}_h^{{\textbf{F}}}:= \{{\textbf{v}}_h \in {\mathcal {V}}_h^2 \, \vert \, {\textbf{v}}_h = {\textbf{F}} \text { on } \partial {{\hat{\Omega }}}\}$ and ${\mathcal {W}} \rightarrow {\mathcal {W}}_h:= {\mathcal {V}}_h^{2 \times 2}$, with ${\mathcal {V}}_h \subset H^1({{\hat{\Omega }}})$.

Similarly, the Newton approach is based on

30 $$\begin{aligned} {\mathcal {N}}^H_{\mu }(X, \Sigma ) := {\mathcal {L}}^H(\gamma _{\mu }^{{{\textbf{x}}}} A_{\mu }^{{{\textbf{x}}}}, X, \Sigma ) \end{aligned}$$

and seeks the increment $\partial X^k \in {\mathcal {U}}^{{\textbf{0}}} \times {\mathcal {W}}^2$ as in (26) by taking the Gateaux derivative of ${\mathcal {N}}_{\mu }^H(\, \cdot \,, \, \cdot \,)$ with respect to its first argument. We discretise in the same way as in the fixed-point iteration.

The Hessian recovery approach increases the problem’s cardinality from $\sim 2 {\text {dim}}({\mathcal {V}}_h )$ to $\sim 10 {\text {dim}}({\mathcal {V}}_h)$. However, we note that ${\mathcal {L}}^H( A(\partial _{{\varvec{\xi }}} {{\textbf{x}}}), \, \cdot \,, \, \cdot \, )$ is nonlinear only in the first term on the right hand side of (27). As such, the linearisation’s bilinear form only needs to be reassembled partially and an efficient implementation can, in fact, operate on the Schur complement of the matrix’s constant blocks, making the cardinality increase manageable in practice. For a more in-depth discourse on an efficient implementation, we refer to [13].

#### Rotation-free approach

This approach adopts the formulation proposed by Gallistl et al. in [14]. Here, we restrict ourselves to the choice $\tau (\, \cdot \,) = \tau _{\text {NS}}(\, \cdot \,)$. Furthermore, for reasons that shall become apparent shortly, we focus exclusively on the fixed-point linearisation.

Rather than directly solving for ${{\textbf{x}}}: {{\hat{\Omega }}}\rightarrow {\Omega }$, the rotation-free approach, in simple terms, is based on a formulation which seeks the map’s Jacobian $J:= \partial _{{\varvec{\xi }}} {{\textbf{x}}}$. In order for $J \in H^1({{\hat{\Omega }}}, {\mathbb {R}}^{2 \times 2})$ to be the gradient of a two-component function, it requires the rows of *J* to be rotation-free for which it introduces a suitable Lagrange multiplier. We introduce the space

$$\begin{aligned} {\mathcal {W}}^{{\textbf{F}}}:= \left\{ ({\textbf{v}}_1, {\textbf{v}}_2) \in H^1({{\hat{\Omega }}}, {\mathbb {R}}^2) \times H^1({{\hat{\Omega }}}, {\mathbb {R}}^2) \, \, \vert \, \, \text {the tangential trace of } {\textbf{v}}_i \text { equals } \partial _{{\textbf{t}}} {\textbf{F}}_i \text { on } \partial {{\hat{\Omega }}}\, \right\} , \end{aligned}$$

where $\partial _{{\textbf{t}}} (\, \cdot \,)$ denotes the tangential derivative along $\partial {{\hat{\Omega }}}$. Furthermore, we introduce

$$\begin{aligned} Q:= \left\{ q \in L^2({{\hat{\Omega }}}) \, \vert \, \smallint \limits _{\partial {{\hat{\Omega }}}} q \, \textrm{d} {\varvec{\xi }}= 0 \right\} . \end{aligned}$$

With $Y:= ({\widehat{J}}, {\textbf{p}}) \in W^{{\textbf{F}}} \times Q^2$ and $\Sigma := (\Phi , {\textbf{q}}) \in {\mathcal {W}}^{{\textbf{0}}} \times Q^2$, the continuous formulation is based on the operator ${\mathcal {L}}^{\text {rot}}: \text {SPD}^{2 \times 2} \times ({\mathcal {W}}^{{\textbf{F}}} \times Q^2) \times ({\mathcal {W}}^{{\textbf{0}}} \times Q^2) \rightarrow {\mathbb {R}}$, with

31 $$\begin{aligned} {\mathcal {L}}^{\text {rot}}(B, Y, \Sigma )&= \int \limits _{{{\hat{\Omega }}}} \left( \nabla \cdot \Phi _i \right) \, B \, :\, \partial _{{\varvec{\xi }}} {\widehat{J}}_i \, \textrm{d} {\varvec{\xi }}\nonumber \\&\quad + \int \limits _{{{\hat{\Omega }}}} \left( \nabla \times \Phi _i \right) {\textbf{p}}_i \, \textrm{d} {\varvec{\xi }}\nonumber \\&\quad + \int \limits _{{{\hat{\Omega }}}} \left( \nabla \times {\widehat{J}}_i \right) {\textbf{q}}_i \, \textrm{d} {\varvec{\xi }}\end{aligned}$$

and $$\nabla \times {\textbf{v}}:= \partial _{\xi _2} {\textbf{v}}_1 - \partial _{\xi _1} {\textbf{v}}_2$$.

The fixed-point linearisation reads:

32 $$\begin{aligned}&\text {find } Y^{k+1} \in {\mathcal {W}}^{{\textbf{F}}} \times Q^2 \quad \text {s.t.} \quad {\mathcal {F}}_\mu ^{\text {rot}}(Y^{k+1}, Y^k, \Sigma ) = 0, \quad \nonumber \\&\quad \forall \Sigma \in {\mathcal {W}}^{{\textbf{0}}} \times Q^2, \end{aligned}$$

with

33 $$\begin{aligned} {\mathcal {F}}_\mu ^{\text {rot}}(Y^{k+1}, Y^k, \Sigma )&:= {\mathcal {L}}^{\text {rot}}(\gamma ^k_\mu A^k_\mu , Y^{k+1}, \Sigma ) \nonumber \\&\quad - \mu {\mathcal {L}}^{\text {rot}}(\gamma ^k_\mu {\mathcal {I}}^{2 \times 2}, Y^k, \Sigma ), \end{aligned}$$

where now $A^k_\mu := A(J^k) + \mu {\mathcal {I}}^{2 \times 2}$, with the *i*-th row of $J^k$ given by ${\widehat{J}}_i^k$ and, as before, $\gamma _\mu ^k:= \gamma (A^k_\mu )$.

For the operator utilised in the discretisation, we have to make two adjustments. Firstly, depending on ${\textbf{F}}: \partial {{\hat{\Omega }}}\rightarrow \partial {\Omega }$, the discrete space ${\mathcal {W}}_h^{{\textbf{F}}}$ will generally be empty. As such, we implement the boundary condition *weakly*. Secondly, as the discrete root $Y^{k+1}_h \in {\mathcal {W}}_h^{{\textbf{F}}} \times Q_h^2$ of (32) generally does not satisfy $\nabla \times {\widehat{J}}_{h, i} = 0$ (pointwise) for $i \in \{1, 2\}$, the discrete problem requires stabilisation. Here, we follow the stabilisation proposed in [14]. Defining

$$\begin{aligned} \epsilon (B):= & {} \min \limits _{{{\hat{\Omega }}}} \frac{{\text {tr}}(B)^2}{\left\| B \right\| ^2_F} - 1, \quad \\ \lambda (\, \cdot \,):= & {} \frac{2 + \sqrt{\alpha \epsilon (\, \cdot \,)}}{2} \quad \text {and} \quad \\ \sigma (\, \cdot \,):= & {} \sqrt{1 - \tfrac{\lambda (\, \cdot \,)}{2}}, \end{aligned}$$

with $0< \alpha < 1$, the stabilisation consists of adding

$$\begin{aligned} {\mathcal {K}}_{\text {stab}}(B, {\widehat{J}}, \Phi ):= & {} \sigma (B) \int \limits _{{{\hat{\Omega }}}} \left( \nabla \times \Phi _i \right) \left( \nabla \times {\widehat{J}}_i \right) \quad \\{} & {} \text {(summing over repeated indices)} \end{aligned}$$

to the right-hand-side of (31). Here, we use $\alpha = 0.9$ and in practice, we approximate $\epsilon (B) \approx \epsilon _h(B)$ by taking the minimum over all evaluations in the abscissae of the quadrature scheme used to compute the integrals. Finally, the stabilised operator ${\mathcal {L}}^{\text {rot}, \text {stab}}: \text {SYM}^{2 \times 2} \times {\mathcal {W}} \times Q^2 \rightarrow {\mathbb {R}}$, along with the weak imposition of the boundary data reads:

34 $$\begin{aligned} {\mathcal {L}}_{\eta }^{\text {rot}, \text {stab}}(B, Y, \Sigma )&:= {\mathcal {L}}^{\text {rot}}(B, Y, \Sigma ) + {\mathcal {K}}_{\text {stab}}(B, {\widehat{J}}, \Phi ) \nonumber \\&\quad + \eta \sum \limits _{L_j \in \Gamma ^B} \frac{1}{h_j} \int \limits _{L_j} \left( \partial _{t} {\textbf{F}} - {\widehat{J}} \, \hat{{\textbf{t}}} \right) \cdot \left( \Phi \hat{{\textbf{t}}} \right) \textrm{d} \Gamma , \end{aligned}$$

where ${\mathcal {W}}:= H^1({{\hat{\Omega }}}, {\mathbb {R}}^{2 \times 2})$ while $h_i$ denotes the average diameter of all knot spans on $L_i \subset \partial {{\hat{\Omega }}}$ and $\hat{{\textbf{t}}}$ denotes the unit tangent along $\partial {{\hat{\Omega }}}$. The factor $\eta > 0$ needs to be taken sufficiently large and in practice, we utilise $\eta = 10^3$.

The discrete problem is subject to the same inf-sup condition as the Stokes problem [14]. Here, we utilise the subgrid space pair [38]. If ${\mathcal {W}}_h = {\mathcal {V}}_h^{2 \times 2}$ and $Q_h$ is constructed by a modification of some finite-dimensional ${\mathcal {U}}_h \subset H^1({{\hat{\Omega }}})$ (to incorporate the zero average condition), this implies that ${\mathcal {V}}_h$ is uniformly *h*-refined with respect to ${\mathcal {U}}_h$. In practice, the space ${\mathcal {U}}_h$ results from removing every other knot in the knotvectors utilised to construct the primal space ${\mathcal {V}}_h$. As such, the cardinality of the problem is $\sim 4.5 \times {\text {dim}}({\mathcal {V}}_h)$ and computational efficiency can be greatly improved by only reassembling the nonlinear part of the fixed-point iteration’s global matrix equation. Here, we only consider the fixed-point iteration due to the required stabilisation. While Newton’s method may be stabilised in a way analogous to the fixed-point iteration, this is beyond the scope of this paper.

It remains to be said that the map ${{\textbf{x}}}: {{\hat{\Omega }}}\rightarrow {\Omega }$ can be recovered by solving:

$$\begin{aligned} \text {find } {{\textbf{x}}}\in {\mathcal {U}}^{{\textbf{F}}} \quad \text {s.t.} \quad \int \limits _{{{\hat{\Omega }}}} \left( \partial _{{\varvec{\xi }}} {{\textbf{x}}}- {\widehat{J}} \, \right) \, :\, \partial _{{\varvec{\xi }}} \varvec{\phi } \, \textrm{d} {\varvec{\xi }}= 0, \quad \forall \varvec{\phi } \in {\mathcal {U}}^{{\textbf{0}}}, \end{aligned}$$

and similarly for the discrete counterpart.

#### $C^0$-DG approach

Having presented two approaches in mixed form, we now proceed to an approach based on the $C^0$-DG formulation from [15]. A $C^0$-DG formulation is particularly appealing as it completely avoids auxiliary variables. Furthermore the discrete basis is (by assumption) sufficiently regular in the interior of patches for penalisation to be restricted to the interior patch interfaces $\gamma _{ij} \in \Gamma ^I$. As opposed to mixed formulations, the $C^0$-DG approach employs the patchwise exact Hessian while weakly imposing continuity of the map’s Jacobian across interior interfaces. Here, we restrict ourselves to the choice $\tau ( \, \cdot \,) = \tau _{\text {NS}}(\, \cdot \,)$. The operator from (19) is adjusted as follows: ${\mathcal {L}} \rightarrow {\mathcal {L}}^{\text {DG}}_{\eta }$, with ${\mathcal {L}}_{\eta }^{\text {DG}}: \text {SPD}^{2 \times 2} \times {\mathcal {U}}^{{\textbf{F}}} \times {\mathcal {U}}^{{\textbf{0}}} \rightarrow {\mathbb {R}}$ satisfying

35 $$\begin{aligned} {\mathcal {L}}^{\text {DG}}_\eta (B, {{\textbf{x}}}, \varvec{\phi })&:= \sum \limits _{k=1}^{N_p} \int \limits _{{{\hat{\Omega }}}_k} \Delta \phi _i B \, :\, H(x_i) \, \textrm{d} {\varvec{\xi }}\nonumber \\&\quad + \eta \sum _{\gamma _{jl} \in \Gamma ^I} \frac{1}{h(\gamma _{jl})} \int \limits _{\gamma _{jl}} [\![ \nabla x_i ]\!] \, :\, [\![ \nabla \phi _i ] \! ] \, \textrm{d} \Gamma . \end{aligned}$$

Here, $[ \! [ \, {\textbf{v}} \, ] \! ]$ denotes the (entry-wise) jump term of ${\textbf{v}} \otimes {\textbf{n}} \in L^2(\gamma _{ij}, {\mathbb {R}}^{2 \times 2})$, with ${\textbf{n}}$ the unit outer normal on $\gamma _{ij}$ in arbitrary but fixed direction while $h(\gamma _{ij})$ denotes the average diameter of all knot spans on the facet $\gamma _{ij}$. The penalisation parameter $\eta > 0$ has to be chosen sufficiently large. In practice, facing geometries with characteristic length scales of ${\mathcal {O}}(1)$, we utilise $\eta = 10$.

The fixed-point iteration as well as the Newton approach are adapted to this formulation simply by replacing ${\mathcal {L}} \rightarrow {\mathcal {L}}^{\text {DG}}_\eta$ in ${\mathcal {F}}_{\mu }(\, \cdot \,, \, \cdot \,, \, \cdot \,)$ and ${\mathcal {N}}_{\mu }(\, \cdot \,, \, \cdot \,)$, respectively, which is not repeated here for the sake of brevity.

### Regularised weak form discretisation

This scheme is based on the weak inverse harmonicity requirement from (14). In what follows, we let ${\mathcal {U}}^{{\textbf{F}}}_{\text {bij}}:= \{{\textbf{v}} \in {\mathcal {U}}^{{\textbf{F}}} \, \vert \, {\textbf{v}} \text { is uniformly nondegenerate} \}$. Noting that ${{\textbf{x}}}^{-1}({\varvec{\xi }}) = {\varvec{\xi }}$ in ${{\hat{\Omega }}}$, a pullback of the weak inverse harmonicity requirement leads to:

36 $$\begin{aligned} \text {find } {{\textbf{x}}}\in {\mathcal {U}}^{{\textbf{F}}}_{\text {bij}} \quad \text {s.t.} \quad {\mathcal {L}}^{\text {W}}({{\textbf{x}}}, \varvec{\phi }) = 0, \quad \forall \varvec{\phi } \in {\mathcal {U}}^{{\textbf{0}}}, \end{aligned}$$

with ${\mathcal {L}}^{\text {W}}: {\mathcal {U}}^{{\textbf{F}}}_{\text {bij}} \times {\mathcal {U}}^{{\textbf{0}}} \rightarrow {\mathbb {R}}$ given by

37 $$\begin{aligned} {\mathcal {L}}^{\text {W}}({{\textbf{x}}}, \varvec{\phi }) := \int \limits _{{{\hat{\Omega }}}}\frac{\partial _{{\varvec{\xi }}} \varvec{\phi } \, :\, A(\partial _{{\varvec{\xi }}} {{\textbf{x}}})}{\det J} \textrm{d} {\varvec{\xi }}\end{aligned}$$

and $A(\, \cdot \,)$ as in (21).

The appearance of $\det J$ in the denominator, as in Winslow’s original approach, prohibits the substitution of degenerate maps, hence the requirement to restrict the domain of ${\mathcal {L}}^W(\cdot , \, \varvec{\phi })$ to ${\mathcal {U}}^{{\textbf{F}}}_{\text {bij}}$ instead of ${\mathcal {U}}^{{\textbf{F}}}$. However, this limits the scope of algorithms based on (36) to improving the parametric quality of an already (uniformly) nondegenerate map. In order to attenuate this harsh requirement, we employ the regularisation proposed in [21, 39], whose original purpose was regularising the Winslow function by replacing

38 $$\begin{aligned} \det J \rightarrow {\mathcal {R}}_{\varepsilon }(\det J), \quad \text {where} \quad {\mathcal {R}}_{\varepsilon }(x) := \frac{x + \sqrt{4 \varepsilon ^2 + x^2}}{2}. \end{aligned}$$

We denote the regularised operator by ${\mathcal {L}}_\varepsilon ^{\text {W}}(\cdot , \, \cdot )$, whose domain is restored to ${\mathcal {U}}^{{\textbf{F}}} \times {\mathcal {U}}^{{\textbf{0}}}$. The asymptotic behaviour of (38) reads

$$\begin{aligned} \lim \limits _{x \rightarrow -\infty } {\mathcal {R}}_\varepsilon (x) = 0 \quad \text {and} \quad \lim \limits _{x \rightarrow \infty } {\mathcal {R}}_{\varepsilon }(x) = x, \quad \text {with} \quad {\mathcal {R}}_{\varepsilon }(0) = \varepsilon . \end{aligned}$$

For $\varepsilon >0$, ${\mathcal {R}}_{\varepsilon } \in C^1({\mathbb {R}})$ and ${\mathcal {R}}_\varepsilon (x) > 0 \, \forall x \in {\mathbb {R}}$. As such, the regularisation can be combined with a gradient-based algorithm acting on (36), such as Newton’s method, again replacing ${\mathcal {U}}^{{\textbf{F}}} \rightarrow {\mathcal {U}}^{{\textbf{F}}}_h$ for a discretisation. Heuristically, $\varepsilon = 10^{-4}$ is a reliable choice as it dramatically increases the radius of convergence and even extends it into the set of degenerate initial iterates in practice. The regularisation is a convenient means to urge a globalised Newton-based root finder to decrease the size of the Newton step in case the updated iterate accidentally leaves the set of NDG maps. In the absence of regularisation, the division by zero typically causes a numerical algorithm to diverge, even when initialised with an NDG initial map.

The value of $\varepsilon > 0$ can be reduced to $\varepsilon = 0$ in an outer loop, in which case it is almost guaranteed that the resulting map is nondegenerate since for $\varepsilon \rightarrow 0$, ${\mathcal {R}}_{\varepsilon }^{-1}(\, \cdot \,)$ acts as a barrier term, as in Winslow’s original approach. While the discrete root of (36) substituted into the Winslow functional typically yields a value slightly greater than Winslow’s global minimiser over ${\mathcal {U}}_h^{{\textbf{F}}}$, we have noticed (36) to converge faster and more reliably than Winslow’s original (regularised) formulation. Furthermore, it is plausible to assume that for $\varepsilon \rightarrow 0$, (36) has a unique root, while the discretisation of Winslow’s approach, may produce local minima.

Compared to the NDF discretisations (cf. Sect. 3.1), the radius of convergence is small. As such, the method is best initialised with one of the NDF discretisations’ solutions, for which it typically converges in no more than 5 Newton iterations. Furthermore, it provides a convenient way of untangling a degenerate map produced by an NDF discretisation without the need to recompute the map over a refined space. Convergence failure of (36) may furthermore indicate that the set ${\mathcal {U}}_{h, \text {bij}}^{{\textbf{F}}}$ is empty, thus making refinement mandatory.

### Boundary correspondence requirements

Without aspirations to provide formal proofs, this section discusses the requirements that ${{\hat{\Omega }}}$, ${\Omega }$ and the boundary correspondence ${\textbf{F}}: \partial {{\hat{\Omega }}}\rightarrow \partial {\Omega }$ have to satisfy in order for the variational formulations of Sect. 3 to be well-posed.

As $A(\partial _{{\varvec{\xi }}} {{\textbf{x}}})$, as defined in (13), has the same characteristic polynomial as the map’s metric tensor $G_{ij} = \partial _{{\varvec{\xi }}_i} {{\textbf{x}}}\cdot \partial _{{\varvec{\xi }}_j} {{\textbf{x}}}$, it is plausible to assume that a necessary condition for well-posedness of the NDF discretisations is that the there are constants $0< C_1 \le C_2 < \infty$ such that the harmonic map ${{\textbf{x}}}^{-1}: {\Omega }\rightarrow {{\hat{\Omega }}}$ satisfies

39 $$\begin{aligned} {C_1 \le \det J({{\textbf{x}}}^{-1}) \le C_2, \quad \text {a.e. in } \Omega .} \end{aligned}$$

While Theorem 1 guarantees that ${{\textbf{x}}}^{-1}: {\Omega }\rightarrow {{\hat{\Omega }}}$ is diffeomorphic in ${\Omega }$, it provides no guarantee that the map is differentiable on the closure ${\overline{{\Omega }}}$ of ${\Omega }$. Failure to satisfy (39) will cause $A(\, \cdot \,)$ to no longer be uniformly elliptic in the exact solution, which may render the problem ill-posed in this case. While this section’s algorithms may nevertheless succeed in finding the discrete problem’s root, we may expect to encounter conditioning issues in the linearisation’s bilinear forms in a refinement study. For $\det J({{\textbf{x}}}^{-1})$ to stay uniformly bounded on the closure, we require that ${\textbf{F}}^{-1}: \partial {\Omega }\rightarrow \partial {{\hat{\Omega }}}$ maps the convex corners of $\partial {\Omega }$ onto the convex corners of $\partial {{\hat{\Omega }}}$ while the smooth segments of $\partial {\Omega }$ are mapped onto straight line segments of $\partial {{\hat{\Omega }}}$ where we furthermore require that $\partial _t {\textbf{F}}^{-1}$ be continuous in the vertices that are mapped onto vertices ${\textbf{v}}_{ij}\in \partial {{\hat{\Omega }}}$ connecting two sides $L_i$ and $L_j$ of $\partial {{\hat{\Omega }}}$ without creating a corner (and similarly for ${\textbf{F}}: \partial {{\hat{\Omega }}}\rightarrow \partial {\Omega }$).

**Fig. 1:**
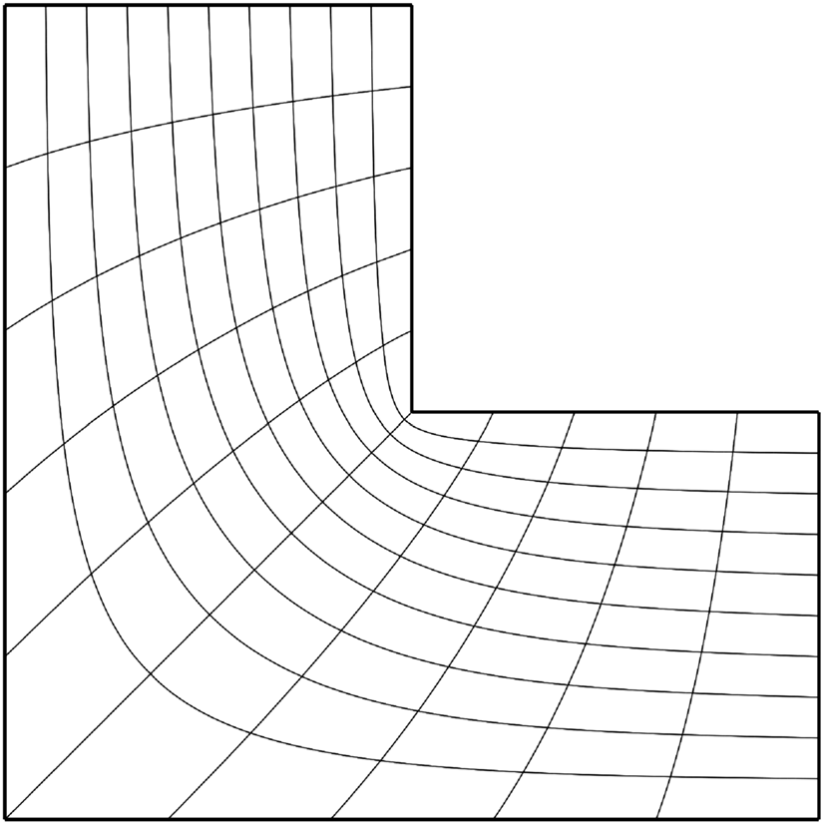
The L-bend geometry computed from a two-patch covering of the unit interval ${{\hat{\Omega }}}= (0, 1)^2$. This geometry provides an example for singularities created by concave corners. The eigenvalue of the map’s metric tensor that corresponds to the direction transversal to the concave corner is given for various refinement levels *h* : 0.0118, *h*/2 : 0.00729, *h*/4 : 0.00453 *h*/8 : 0.00284. Each map has been computed by minimising the Winslow function (9)

Clearly, mapping a straight line segment of ${\overline{L}}_i \cup {\overline{L}}_j \subset \partial {{\hat{\Omega }}}$ onto two sides ${\overline{C}}_i \cup {\overline{C}}_j \subset \partial {\Omega }$ with a convex corner in the shared vertex will cause $\det J({{\textbf{x}}}) \rightarrow \infty$ in the vertex connecting $L_i$ and $L_j$. Similarly, mapping the same vertex onto a vertex of $\partial {\Omega }$ that creates a concave corner will cause a singularity, i.e., $\det J({{\textbf{x}}}) \rightarrow 0$. While discrete approximations typically remain UNDG, this behaviour will be observable in a refinement study, see Fig. 1. Since the weak-form operator ${\mathcal {L}}^W_{\varepsilon }: {\mathcal {U}}^{{\textbf{F}}} \times {\mathcal {U}}^{{\textbf{0}}} \rightarrow {\mathbb {R}}$ may map into ${\mathbb {R}}$ despite division by zero on $\partial {{\hat{\Omega }}}$, even for $\varepsilon \rightarrow 0$, it may allow for boundary correspondences that exhibit less regularity. Notwithstanding the well-posedness of the formulation, we regard discrete maps ${{\textbf{x}}}_h: {{\hat{\Omega }}}\rightarrow {\Omega }$ that approximate a map ${{\textbf{x}}}: {{\hat{\Omega }}}\rightarrow {\Omega }$ with a singularity on $\partial {{\hat{\Omega }}}$ as undesirable from a numerical perspective.

In what follows, we refer to a boundary correspondence ${\textbf{F}}: \partial {{\hat{\Omega }}}\rightarrow \partial {\Omega }$ that satisfies above requirements as a *diffeomorphic boundary correspondence*. Section 4 augments this section’s formulations with a mechanism that allows for the creation of diffeomorphic boundary correspondences even when $\partial {\Omega }$ has no corners.

### Computational results

In this section, we apply the algorithms developed in Sects. 3.1.3–3.1.5 and 3.2 to a number of example geometries. In all cases, we utilise a combination of one of the NDF discretisations and the regularised weak form discretisation to approximate an inversely harmonic map. The NDF discretisations are initialised with a map that approximates a pair of harmonic functions in ${{\hat{\Omega }}}$ (with the prescribed boundary correspondence). The weak form discretisation is then initialised with the NDF discretisation’s solution. Convergence of the NDF scheme is typically achieved within  5 Newton or  12 fixed-point iterations while the weak form discretisation requires a further $3-5$ Newton iterations. We emphasise that the choice of ${{\hat{\Omega }}}$, along with its multipatch covering, remain a manual process. All schemes, as well as all control mechanisms from Sect. 4 have been implemented in the open-source finite element library *Nutils* [40].

**Fig. 2:**
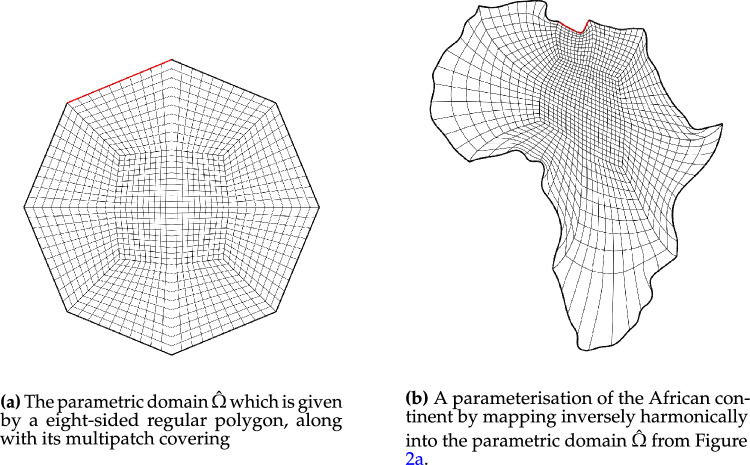
The parametric domain and the approximation of a harmonic map of the African continent. The two domain’s sides are paired in counterclockwise order, starting from the edge marked in red (Color figure online)

**Fig. 3:**
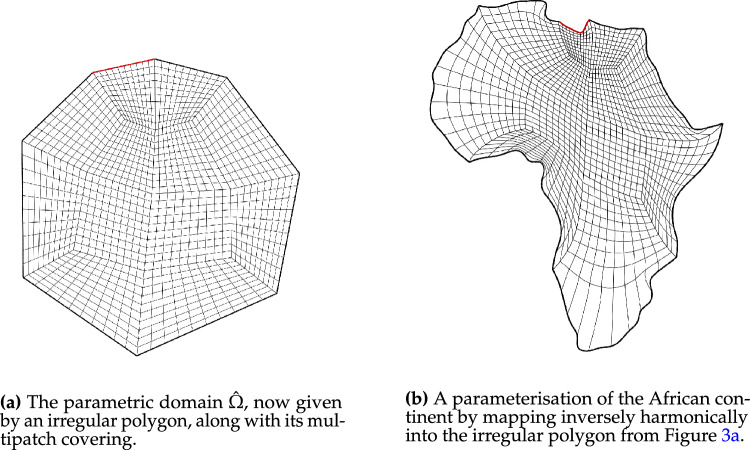
The parametric domain and the approximation of a harmonic map of the African continent using a irregular polygonal domain ${{\hat{\Omega }}}$ with a bilinear multipatch covering that results from mapping the covering from Fig. 2a approximately harmonically into ${{\hat{\Omega }}}$

Fig. 2 shows a parameterisation of the African continent (Fig. 2b) from a regular eight-sided polygonal parametric domain whose multipatch covering is shown in Fig. 2a. The operator ${{\textbf{x}}}: {{\hat{\Omega }}}\rightarrow {\Omega }$ maps the eight boundary edges $L_k \subset \partial {{\hat{\Omega }}}$ onto the eight sides $C_k \subset \partial {\Omega }$ in counterclockwise orientation, starting from the highlighted edge pair. In Fig. 3, the regular polygonal parametric domain is substituted with an irregular polygon. This transition involves a multipatch covering derived by an approximately harmonic mapping of the interior vertices from the covering depicted in Fig. 2a into the irregular polygon shown in Fig. 3a, achieved through the application of a bilinear nodal basis on the patch vertices. Meanwhile, the irregular polygon’s boundary vertices are positioned on the unit circle so that each boundary edge’s length $\vert L_k \vert$, relative to the total perimeter $\vert \partial {{\hat{\Omega }}}\vert$, mirrors the corresponding side’s length $\vert C_k \vert$ in proportion to $\partial {\Omega }$’s total length.

The outcome is a notably enhanced patch surface area homogeneity under ${{\textbf{x}}}: {{\hat{\Omega }}}\rightarrow {\Omega }$, as illustrated in Fig. 3, compared to Fig. 2. Nonetheless, in both instances, the mapping generates larger elements near the geometry’s significantly protruding lower section-a recognized pathology inherent to harmonic maps. This paper addresses this pathology and proposes a solution in Sect. 4.3. The example illustrates how the quality of the parameterization significantly depends on the selection of the parametric domain and its covering. This observation, along with efforts to systematically choose the parametric domain, will be further explored in an upcoming publication.

**Fig. 4:**
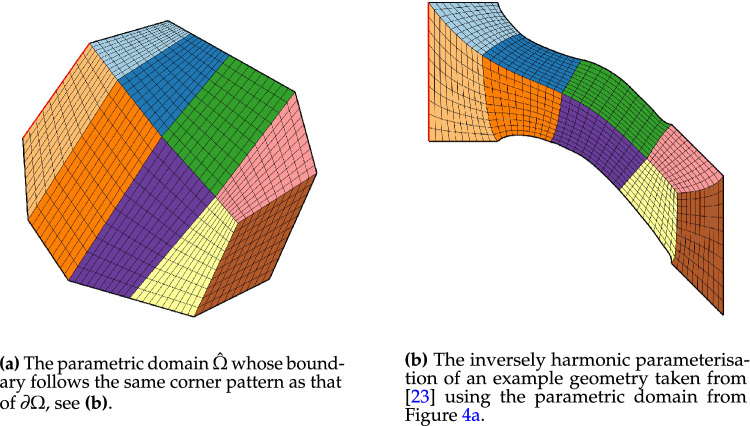
An inversely harmonic map from the parametric domain depicted in **a** onto the physical domain from **b**. Individual patches are highlighted in different colors (Color figure online)

As a second example, we are considering the geometry along with its parametric domain from Fig. 4, which is an example taken from [23]. The parametric domain’s outlines have been generated using the same methodology as in Fig. 3 under the additional requirement that no corners be created between two neighboring sides $\{L_k, L_{k+1}\}$ of $\partial {{\hat{\Omega }}}$ if the associated curves $\{C_k, C_{k+1} \}$ create no corner on $\partial {\Omega }$. We are utilising the same multipatch layout as in [23] and the harmonic map is similar to a result produced using nonlinear optimisation (for details, see [23]).

In contrast to nonlinear optimization, which requires parameter tuning and faces the risk of entrapment in local minima or folding of the minimiser, the harmonic map-based methodology necessitates no more than the careful positioning of the quadrangulation’s four interior vertices within the convex domain ${{\hat{\Omega }}}$.

### Numerical experiments

In this section, we apply the algorithms from Sect. 3 to a number of benchmark test cases to experimentally determine the scheme’s convergence rates.

**Fig. 5:**
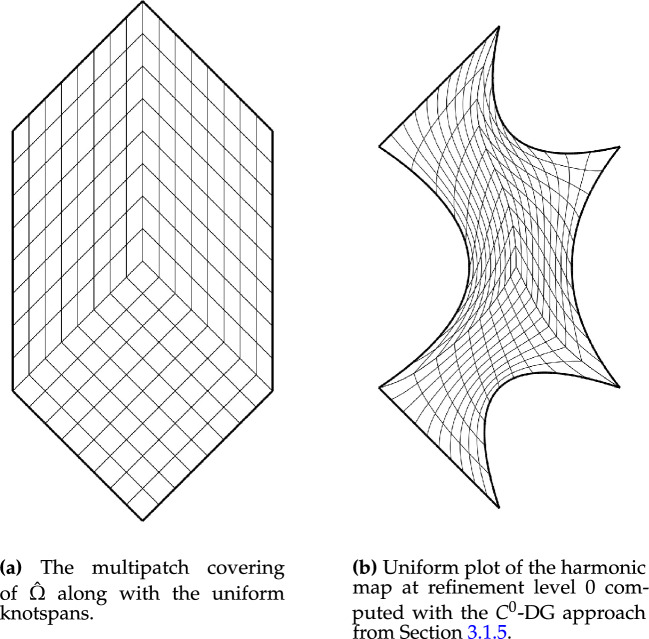
The parametric domain and the approximation of a harmonic map for a bat-shaped geometry

As a first experiment we are considering the bat shaped geometry from Fig. 5b along with the parametric domain from Fig. 5a. The geometry is a piecewise $C^\infty$ curvilinear polygon whose sides $C_i$ are quadratic polynomials. We therefore expect the harmonic map ${{\textbf{x}}}^{-1}: {\Omega }\rightarrow {{\hat{\Omega }}}$ to satisfy ${{\textbf{x}}}^{-1} \in H^s({\Omega }, {\mathbb {R}}^2)$ with $2 \le s \le 3$. The boundary correspondence ${\textbf{F}}: \partial {{\hat{\Omega }}}\rightarrow \partial {\Omega }$ (suitably extended into the interior) can be expressed exactly in any finite-dimensional space ${\mathcal {V}}_h$ with polynomial degree $p \ge 2$, thanks to its piecewise quadratic nature. We are estimating the convergence rate for all three NDF discretisations (cf. Sect. 3.1) as well as the weak form discretisation from Sect. 3.2 and Winslow’s original approach (cf. Sect. 2.1). As before, the NDF discretisations are initialised with a map that approximates a pair of harmonic functions in ${{\hat{\Omega }}}$ (with the prescribed boundary correspondence) while the weak form discretisation is initialised with the $C^0$-DG approach’s solution and Winslow’s minimisation with the weak form discretisation’s solution. This is repeated for several levels of *h*-refinement, each splitting each univariate knotspan in half (without changing the boundary correspondence). For the rotation-free approach, we perform a fixed-point iteration while all other linearisations are based on Newton’s method. The convergence rate is estimated in the $H^1({{\hat{\Omega }}})$-norm. Denoting three consecutive solutions by ${{\textbf{x}}}_h$, ${{\textbf{x}}}_{h/2}$ and ${{\textbf{x}}}_{h/4}$, respectively, the convergence rate is estimated as

$$\begin{aligned} \kappa \approx \log _2 \left( \frac{\left\| {{\textbf{x}}}_h - {{\textbf{x}}}_{h/2} \right\| _{H^1({{\hat{\Omega }}})}}{\left\| {{\textbf{x}}}_{h/2} - {{\textbf{x}}}_{h/4} \right\| _{H^1({{\hat{\Omega }}})}} \right) , \end{aligned}$$

and we utilise the last three levels of refinement to estimate $\kappa$ in the above. We perform a refinement study assigning a uniform knotvector without internal knot repetitions to each interior and boundary facet whereby the coarsest knotvector contains three interior knots.

**Fig. 6:**
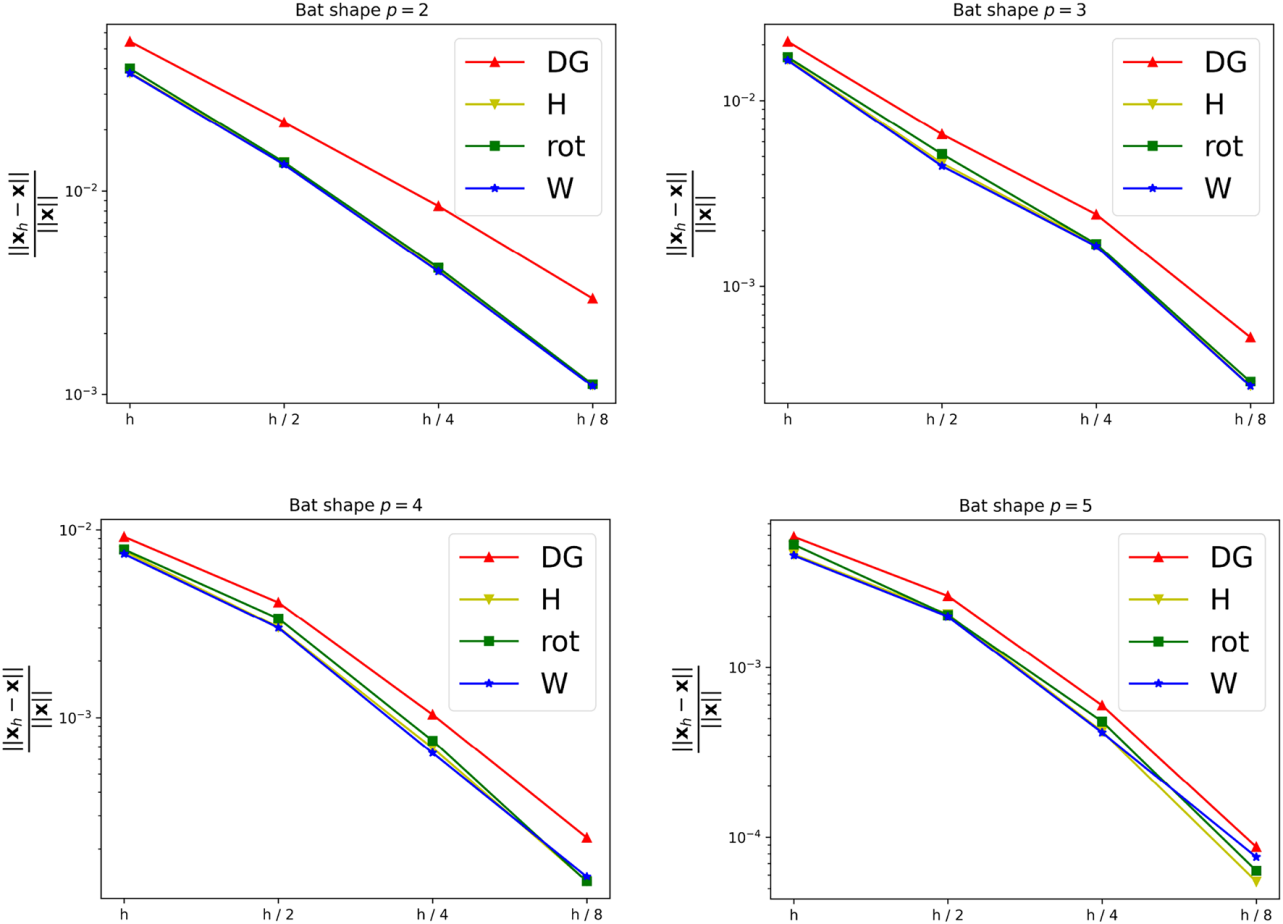
Convergence behaviour of the various discretisations applied to the bat shaped geometry from Fig. 5 for different values of the polynomial degree *p*. Here the labels, in the order of appearance, refer to the $C^0$-DG (Sect. 3.1.5), Hessian-recovery (Sect. 3.1.3) and the rotation-free approach (Sect. 3.1.4) as well as the regularised weak-form discretisation (Sect. 3.2)

**Table 1:** Approximate convergence rates for the bat shape geometry for various values of the polynomial degree *p*

*p*	2	3	4	5
$\kappa \left( {\mathcal {L}}^{\text {DG}}\right)$	1.37	1.36	2.08	2.04
$\kappa \left( {\mathcal {L}}^H \right)$	1.68	1.32	2.09	2.22
$\kappa \left( {\mathcal {L}}^{\text {rot}} \right)$	1.66	1.61	2.13	2.01
$\kappa \left( {\mathcal {L}}^{\text {W}} \right)$	1.68	1.31	2.19	2.22
$\kappa \left( \text {Winslow} \right)$	1.75	1.32	2.16	2.31

**Fig. 7:**
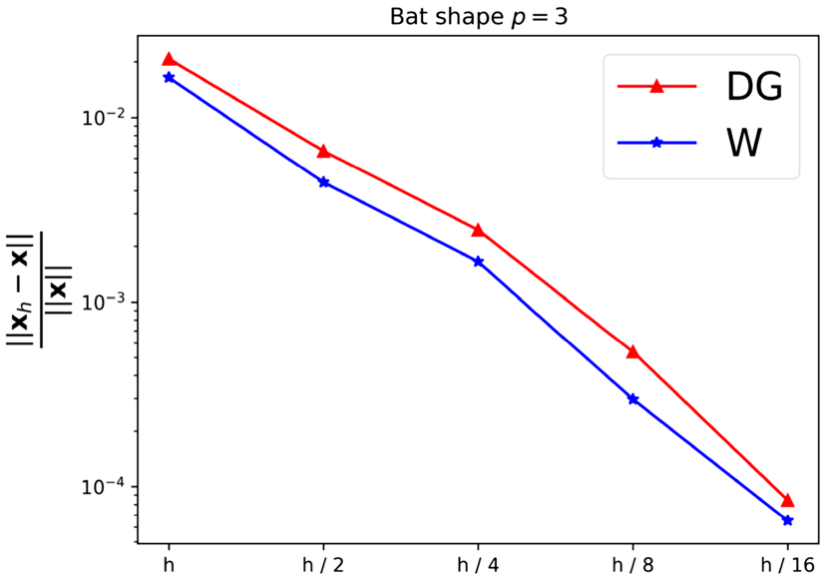
Convergence behaviour of the $C^0$-DG and the weak form discretisation with one additional level of refinement compared to Fig. 6

Table 1 contains the approximate convergence rates of this section’s discretisations for various values of the polynomial degree $p \ge 2$ while Fig. 6 plots the relative $H^1$-norm discrepancy between the various refinement level’s solutions and the exact solution which is approximated by minimising the Winslow function over a spline space that is one level of refinement ahead of the maximum refinement level in the plots (here: *h*/16). The table and plots suggest that all discretisations perform similarly well, a notable exception being the $C^0$-DG approach from Sect. 3.1.5 which consistently produces the largest relative $H^1$-norm discrepancy. We note however that the $C^0$-DG approach is the computationally least expensive method that can be initialised with a degenerate map. The approximate convergence rates improve for larger values of $p \ge 2$, eventually reaching saturation for $p = 5$. This is not surprising given that it is plausible to assume that the largest attainable $H^1({{\hat{\Omega }}}, {\mathbb {R}}^2)$ convergence rate is bounded by the value of $2 \le s \le 3$ associated with the harmonic map ${{\textbf{x}}}^{-1} \in H^s({\Omega }, {\mathbb {R}}^2)$. A notable exception is the outcome for $p = 3$, which consistently ranks below all other choices. Figure 7 depicts the convergence behaviour of the $C^0$-DG and weak form discretisations with one additional level of refinement. Applying the convergence rate estimator to the last three consecutive levels of refinement yields $\kappa ({\mathcal {L}}^{\text {DG}}) \approx 2.18$, $\kappa ({\mathcal {L}}^{\text {W}}) \approx 2.43$ and $\kappa (\text {Winslow}) \approx 2.48$. This suggests that the results of Table 1, which correspond to refinement levels that are of practical interest, are in the non-asymptotic convergence regime while the asymptotic convergence rate (which corresponds to a practically undesirably large number of DOFs) is slightly better than the table suggests. However, given that the choice $p=2$ produces a convergence log-plot comprised of nearly straight lines, the results of Table 1 suggest that the convergence rate is slightly below the for $p=2$ maximally attainable rate of $\kappa (\, \cdot \,) = 2$ in this case, most likely as a result of the nonlinearity. Overall, the results suggest that the two NDF discretisations in mixed form perform similarly well while slightly outperforming the $C^0$-DG approach at the expense of higher computational costs. Of all the discretisations presented in Sect. 3, the regularised weak form discretisation consistently produces the best results. In fact, it performs only marginally worse than Winslow’s original approach, yet converging significantly faster and more reliably while furthermore avoiding local minima in practice. In our practical experience the $C^0$-DG approach, despite being outperformed by the discretisations in mixed form, suffices for the purpose of finding a nondegenerate or nearly nondegenerate initial iterate for initialising the regularised weak form discretisation in the vast majority of cases. As such, a combination of the two methods constitutes the best trade-off between robustness, solution quality and computational costs.

All NDF discretisations converge very reliably in typically  5 iterations when initialised with a map comprised of a pair of harmonic functions in ${{\hat{\Omega }}}$, making them suitable for the use in autonomously operating workflows. In autonomous workflows, combining an NDF discretisation with a posteriori refinement in case nondegeneracy does not carry over to the numerical approximation constitutes the most robust choice. Here, a mixed-form discretisation becomes a viable choice thanks to the better convergence rate. While the computational costs are higher, they remain manageable when operating on the Schur complement of the bilinear form’s constant blocks. Overall, the Hessian recovery approach tends to be the better choice in this case, despite the problem’s larger cardinality compared the the rotation-free approach, since it can be combined with Newton’s method. Accelerating the convergence of the rotation-free approach constitutes a topic for future research. For this, it may be possible to adopt a multipatch generalisation of the preconditioned Anderson acceleration approach from [41] which is highly effective in the singlepatch case.

The ad hoc formulation from [11], is based on a gradient recovery approach which requires introducing a total of four auxiliary variables taken from a space which is uniformly refined in either *p* or *h* with respect to the primal space ${\mathcal {V}}_h$. As such, this formulation increases the problem’s cardinality from $\sim 2 \dim ({\mathcal {V}}_h)$ to $\sim 18 \dim ({\mathcal {V}}_h)$. We conclude that all three NDF formulations (besides enhanced mathematical rigour) provide a drastic cardinality reduction over [11], in particular the $C^0-DG$ approach which differs from a conforming discretisation by no more than a number of interior penalty terms over the quadrangulation’s interfaces. The slowdown caused by the Hessian recovery approach’s DOF proliferation from $\sim 2 \dim ({\mathcal {V}}_h)$ to $\sim 10 \dim ({\mathcal {V}}_h)$ may be drastically reduced by adopting the formulation from [16], which allows for a local reconstruction of the Hessian from a patchwise continuous but globally discontinuous auxiliary basis (thus introducing computationally advantageous diagonal blocks in the problem’s nonlinear Schur complement).

**Fig. 8:**
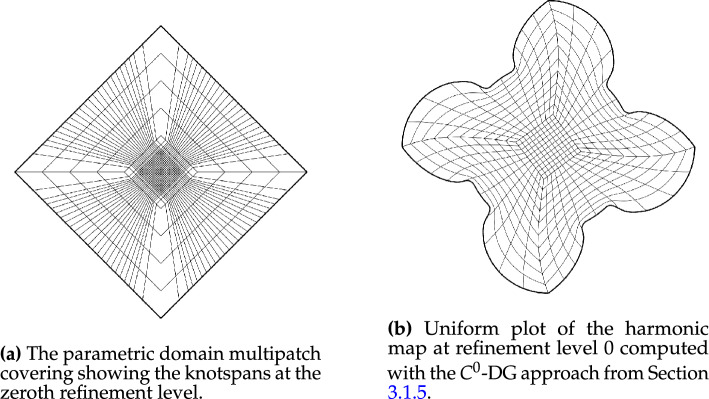
The parametric domain and the approximation of a harmonic map for a screw geometry

**Fig. 9:**
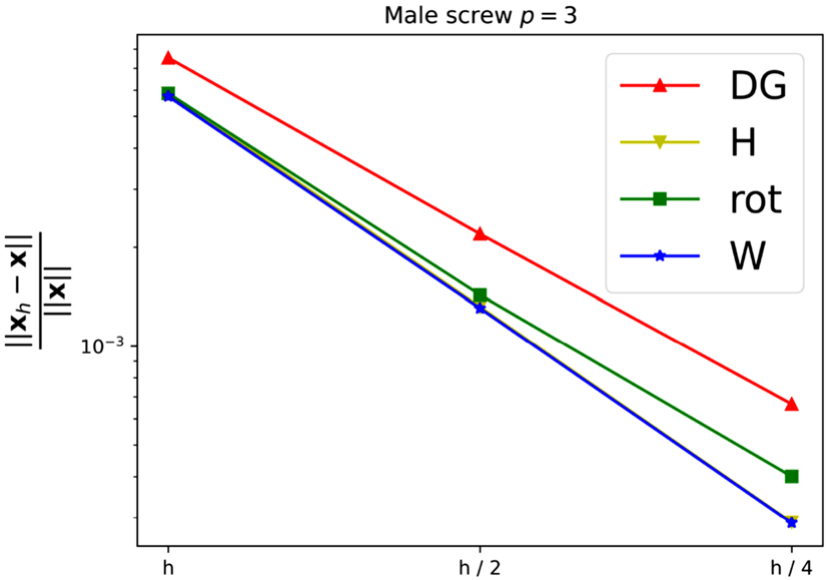
Convergence plot of the various methods applied to the male screw geometry from Fig. 8 with $p=3$

As a final example, we are considering the screw geometry from Fig. 8b which is mapped inversely harmonically into the parametetric domain from Fig. 8a, which also shows the knotspans of the bicubic knotvectors with maximum regularity on each individual patch. Since the boundary correspondence is itself a piecewise bicubic spline with maximum regularity, we are only considering the choice $p=3$ here. The convex corners of ${{\hat{\Omega }}}$ are mapped onto the convex corners of ${\Omega }$ in the counterclockwise direction. Table 2 contains the approximate convergence rates for the various numerical schemes while Fig. 9 shows the approximate $H^1$-norm distance to the exact solution, as before.

**Table 2:** Approximate convergence rates for the male screw geometry using the various numerical schemes

$p = 3$	$\kappa \left( {\mathcal {L}}^H \right)$	$\kappa \left( {\mathcal {L}}^{\text {rot}} \right)$	$\kappa \left( {\mathcal {L}}^{\text {DG}}\right)$	$\kappa \left( {\mathcal {L}}^{\text {W}}\right)$	$\kappa \left( \text {Winslow} \right)$
	2.12	1.97	1.78	2.13	2.12

From the table and plot we may largely draw the same conclusions as in the previous example with the $C^0$-DG approach being outperformed by the other methods while the weak form discretisation fares the best, even slightly outperforming Winslow’s method in this example.

## Control mechanisms

### Techniques for parametric control

The parameterisations generated by Winslow’s original approach (9) or its PDE-based counterparts perform well on a wide range of benchmark problems [5]. However, as individual applications may require parameterisations with specific features, such as boundary layers in flow problems or homogeneous cell-sizes in problems subject to a CFL-condition, the techniques from Sect. 3 may be too rigid. Clearly, choosing the multipatch covering ${\mathcal {Q}}$ based on the application’s specific needs may provide relief. However, in practice, this may prove too restrictive since the covering remains bilinear, which does not, for instance, allow for the creation of boundary layers.

This section introduces a framework that enables controlling the parametric properties of the map produced by one of the algorithms from Sect. 3. Parametric control can be achieved in two main ways:

1. Augmenting the standard (inverse) Laplace problem with a nonhomogeneous diffusivity.
2. Mapping inversely harmonically into a parametric domain with a curvilinear rather than a Cartesian coordinate system.

Let $$\varvec{\phi }: {\Omega }_1 \rightarrow {\mathbb {R}}^2$$ satisfy:

40 $$\begin{aligned} i \in \{1, 2\}: \quad \nabla \cdot \left( D \nabla \varvec{\phi }_i \right) = 0 \quad \text {in } {\Omega }_1, \quad \text {s.t. } \varvec{\phi } = {\textbf{F}} \text { on } \partial {\Omega }_1. \end{aligned}$$

For point 1., we state the following theorem [42]:

#### Theorem 2

(Divergence-form equations) Let $D \in \text {SPD}^{2 \times 2}({\Omega }_1)$ be uniformly elliptic and let $\varvec{\phi } \in H^1({\Omega }_1, {\mathbb {R}}^{2}) \cap C^0({\overline{{\Omega }}}_1, {\mathbb {R}}^2)$ be the weak solution of (40). If ${\textbf{F}}$ is diffeomorphic between $\partial {\Omega }_1$ and $\partial {\Omega }_2$ and ${\Omega }_2$ is convex, then $\varvec{\phi }: {\Omega }_1 \rightarrow {\Omega }_2$ satisfies $\det \partial _{{{\textbf{x}}}} \varvec{\phi } \ge 0$ a.e. in ${\Omega }_1$.

Under stronger regularity requirements on *D*, ${\Omega }_1$ and ${\Omega }_2$, Theorem 2 can be extended to uniform nondegeneracy $\det \partial _{{{\textbf{x}}}} \varvec{\phi } > 0$ (a.e. in ${\Omega }_1$). For details we refer to [42]. This means in particular that for merely essentially bounded $D \in \text {SPD}^{2 \times 2}({\Omega })$, we need to account for the possibility of $\det \partial _{{\varvec{\xi }}} {{\textbf{x}}}\rightarrow 0$ or $\det \partial _{{\varvec{\xi }}} {{\textbf{x}}}\rightarrow \infty$ in the interior of ${{\hat{\Omega }}}$, which may require stabilisation. Here, the need for stabilisation has to be assessed on a case-to-case basis.

Taking ${\Omega }_1 = {\Omega }$ and ${\Omega }_2 = {{\hat{\Omega }}}$, it is reasonable to assume that Theorem 2 also applies to, for instance, the weak-form approach from Sect. 3.2, even though it exchanges the dependencies, i.e., ${\varvec{\xi }}({{\textbf{x}}}) \rightarrow {{\textbf{x}}}({\varvec{\xi }})$. A limitation of introducing a nonhomogeneous diffusivity is that it is currently unknown whether the inverted problem can be cast into a form that does not contain the Jacobian determinant in the denominator, as in the NDF-discretisation from Sect. 3.1. However, the NDF-discretisations remain highly practical since they can compute a nondegenerate reference solution to initialise an iterative scheme with $D \ne {\mathcal {I}}^{2 \times 2}$ based on the weak-form discretisation.

For point 2., a coordinate transformation is conveniently accomplished by introducing a controlmap ${{\textbf{r}}}: {{\hat{\Omega }}}\rightarrow {{\hat{\Omega }}^{{\textbf{r}}}}$. As such, we now allow the target domain of $\left( {{\textbf{x}}}^{{{\textbf{r}}}} \right) ^{-1}: {\Omega }\rightarrow {{\hat{\Omega }}^{{\textbf{r}}}}$ to be a parametric surface, too. In what follows, differential operators receive a subscript to indicate differentiation w.r.t. various coordinate systems. For instance, $\nabla \rightarrow \nabla _{{{\textbf{r}}}}$ to indicate differentiation w.r.t. the entries of ${{\textbf{r}}}: {{\hat{\Omega }}}\rightarrow {{\hat{\Omega }}^{{\textbf{r}}}}$.

The introduction of ${{\textbf{r}}}: {{\hat{\Omega }}}\rightarrow {{\hat{\Omega }}^{{\textbf{r}}}}$ furthermore enables creating boundary correspondences ${\textbf{F}}^{{{\textbf{r}}}\rightarrow {{\textbf{x}}}}: \partial {{\hat{\Omega }}^{{\textbf{r}}}}\rightarrow \partial {\Omega }$ that are diffeomorphic between ${{\hat{\Omega }}^{{\textbf{r}}}}$ and ${\Omega }$ when ${\Omega }$ has no corners by, for instance, choosing ${{\hat{\Omega }}^{{\textbf{r}}}}$ to be the unit disc. For Theorem 1 to apply to the pair $({{\hat{\Omega }}^{{\textbf{r}}}}, {\Omega })$, we require ${{\hat{\Omega }}^{{\textbf{r}}}}$ to be convex. We denote the map that maps inversely harmonically into the domain ${\Omega }^{{{\textbf{r}}}} = {{\textbf{r}}}({{\hat{\Omega }}})$ by ${{\textbf{x}}}^{{{\textbf{r}}}}({{\textbf{r}}}): {{\hat{\Omega }}^{{\textbf{r}}}}\rightarrow {\Omega }$. The same map can be converted to the original coordinate system via a pullback. We employ the abuse of notation ${{\textbf{x}}}^{{{\textbf{r}}}}({{\textbf{r}}})$ instead of ${{\textbf{x}}}({{\textbf{r}}}({\varvec{\xi }}))$ to indicate a change of coordinate system and assume that the reader is aware of the compositions involved.

**Fig. 10:**
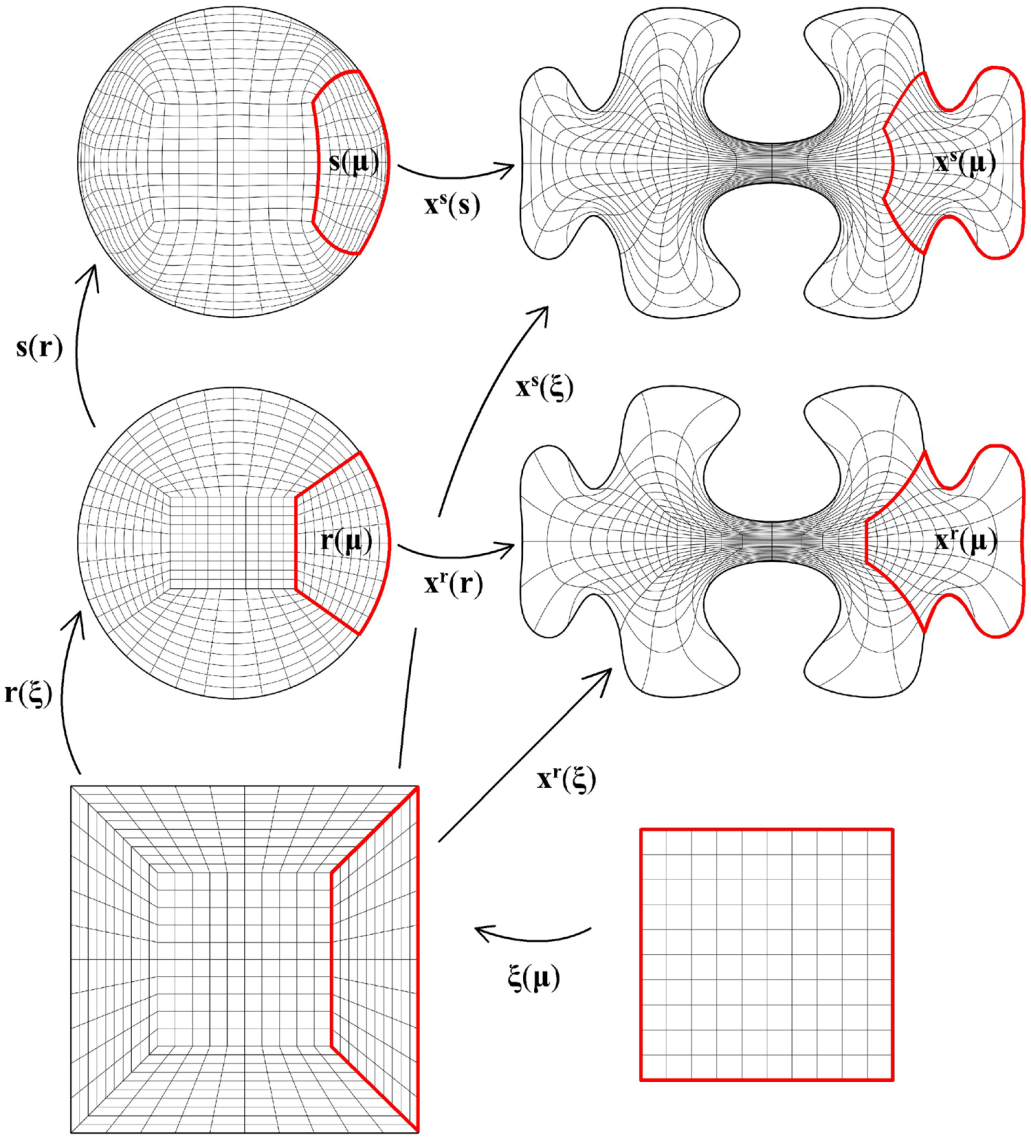
Figure summarising the dependencies between ${{\textbf{x}}}, {{\textbf{s}}}, {{\textbf{r}}}, {\varvec{\xi }}$ and ${\varvec{\mu }}$

In the IGA-setting, parametric control via ${{\textbf{r}}}: {{\hat{\Omega }}}\rightarrow {{\hat{\Omega }}^{{\textbf{r}}}}$ is conveniently achieved by reinterpreting the PDE-based formulations over the Cartesian coordinate system ${\varvec{\xi }}= (\xi _1, \xi _2)^T$ as problems posed over ${\hat{{\Omega }}}^{{{\textbf{r}}}}$, with the curvilinear coordinate system induced by ${{\textbf{r}}}({\varvec{\xi }}) = (r_1(\xi _1, \xi _2), r_2(\xi _1, \xi _2))^T$. We may then use basic differential geometry identities to express the associated integrals in the original coordinate system via a pullback. As such, the operators from Sect. 3 now receive an additional ${{\textbf{r}}}$-dependence. However, this does not change the nature of the equations as long as the ${{\textbf{r}}}\circ {{\textbf{m}}}^i$ remain diffeomorphisms.

Given a reference controlmap ${{\textbf{r}}}: {{\hat{\Omega }}}\rightarrow {{\hat{\Omega }}^{{\textbf{r}}}}$, we may find a new controlmap ${{\textbf{s}}}({{\textbf{r}}}): {{\hat{\Omega }}^{{\textbf{r}}}}\rightarrow {{\hat{\Omega }}^{{\textbf{r}}}}$ that induces a coordinate transformation in ${{\hat{\Omega }}}^{{{\textbf{r}}}}$ and builds desired features into the recomputed map ${{\textbf{x}}}^{{{\textbf{r}}}}({\varvec{\xi }}): {{\hat{\Omega }}}\rightarrow {\Omega }$. A major advantage of operating in ${{\hat{\Omega }}}^{{{\textbf{r}}}}$ rather than on ${\Omega }$ directly is that the former is convex. As such, we have a larger arsenal of methods that reliably produce a UNDG controlmap ${{\textbf{s}}}: {{\hat{\Omega }}}^{{{\textbf{r}}}} \rightarrow {{\hat{\Omega }}}^{{{\textbf{r}}}}$ at our disposal. Computationally inexpensive algebraic methods, for instance, tend to be very robust on convex domains, but not on nonconvex ones. This section provides a glimpse of what can be accomplished via the controlmap approach and serves the purpose of demonstrating the true potential of the algorithms from Sect. 3 in practical applications. In this paper, we present a handful of techniques based on the controlmap concept. More advanced techniques will be the topic of a follow-up publication.

As in point 1., a broad class of reparameterisation methods follows from seeking the controlmap as the solution of (40). More precisely, given a reference controlmap ${{\textbf{r}}}: {{\hat{\Omega }}}\rightarrow {{\hat{\Omega }}^{{\textbf{r}}}}$, we may seek ${{\textbf{s}}}({{\textbf{r}}}): {{\hat{\Omega }}^{{\textbf{r}}}}\rightarrow {{\hat{\Omega }}^{{\textbf{r}}}}$ as the solution of (40) with ${\Omega }_1 = {\Omega }_2 = {{\hat{\Omega }}^{{\textbf{r}}}}$ while selecting a diffusivity *D* that builds desired features into the solution. The map ${{\textbf{s}}}({\varvec{\xi }}): {{\hat{\Omega }}}\rightarrow {\Omega }^{{{\textbf{r}}}}$ then follows from a pullback and a diffeomorphic boundary correspondence ${\textbf{F}}^{{{\textbf{r}}}\rightarrow {{\textbf{s}}}}: \partial {{\hat{\Omega }}^{{\textbf{r}}}}\rightarrow {{\hat{\Omega }}^{{\textbf{r}}}}$ is given by the identity.

If ${{\textbf{s}}}({{\textbf{r}}}): {{\hat{\Omega }}^{{\textbf{r}}}}\rightarrow {{\hat{\Omega }}^{{\textbf{r}}}}$ is the identity on $\partial {{\hat{\Omega }}^{{\textbf{r}}}}$, the effect of the coordinate transformation, induced by ${{\textbf{s}}}({\varvec{\xi }}): {{\hat{\Omega }}}\rightarrow {{\hat{\Omega }}^{{\textbf{r}}}}$, on ${{\textbf{x}}}^{{{\textbf{s}}}}({\varvec{\xi }}): {{\hat{\Omega }}}\rightarrow {\Omega }$ can be predicted by noting that ${{\textbf{x}}}^{{{\textbf{s}}}}({\varvec{\xi }}) = {{\textbf{x}}}^{{{\textbf{r}}}} \circ {{\textbf{s}}}({\varvec{\xi }})$. All the dependencies between ${{\textbf{x}}}, {{\textbf{s}}}, {{\textbf{r}}}, {\varvec{\xi }}$ and the local coordinate system ${\varvec{\mu }}$ in $\Omega ^{\square }$ are summarised in Fig. 10.

As before, depending on the regularity of the diffusivity, the solutions may contain singularities $\det J \rightarrow 0$ or unbounded growth, i.e., $\det J \rightarrow \infty$. While the vanishing or diverging of $\det J$ is typically avoided by discrete approximations, this behaviour will be observable in a refinement study. For the ${{\textbf{s}}}({\varvec{\xi }}) \circ {{\textbf{m}}}^i$ to be diffeomorphisms, this means that for ${{\textbf{s}}}\in H^1({{\hat{\Omega }}^{{\textbf{r}}}}, {\mathbb {R}}^{2})$, jumps in the Jacobian $\partial _{{{\textbf{r}}}} {{\textbf{s}}}({{\textbf{r}}})$ may only occur on the ${{\textbf{r}}}(\partial {{\hat{\Omega }}}_i)$. As such, we require the diffusivity to be patchwise continuous.

Given a reference controlmap ${{\textbf{r}}}: {{\hat{\Omega }}}\rightarrow {{\hat{\Omega }}^{{\textbf{r}}}}$, the most general approach combines methods 1. and 2., leading to the coupled system

41 $$\begin{aligned}&\text {find } ({{\textbf{x}}}({{\textbf{r}}}), {{\textbf{s}}}({{\textbf{r}}})) \text { s.t. for } i \in \{1, 2 \}: \nonumber \\&\quad \left\{ \begin{array}{c} \nabla _{{{\textbf{x}}}} \cdot \left( D^{{{\textbf{x}}}} \nabla _{{{\textbf{x}}}} {{\textbf{s}}}_i \right) = 0 \\ \nabla _{{{\textbf{r}}}} \cdot \left( D^{{{\textbf{s}}}} \nabla _{{{\textbf{r}}}} {{\textbf{s}}}_i \right) = 0 \end{array} \right. \text { in } {{\hat{\Omega }}^{{\textbf{r}}}}\quad \text {and} \quad \left\{ \begin{array}{l} {{\textbf{x}}}= {\textbf{F}}^{{{\textbf{r}}}\rightarrow {{\textbf{x}}}} \\ {{\textbf{s}}}= {\textbf{F}}^{{{\textbf{r}}}\rightarrow {{\textbf{s}}}} \end{array} \right. \text { on } \partial {{\hat{\Omega }}^{{\textbf{r}}}}, \end{aligned}$$

where, typically, ${\textbf{F}}^{{{\textbf{r}}}\rightarrow {{\textbf{s}}}}({{\textbf{r}}}) = {{\textbf{r}}}$. Here, the practically useful dependencies are $D^{{{\textbf{x}}}} = D^{{{\textbf{x}}}}({{\textbf{s}}}, {{\textbf{x}}})$ and $D^{{{\textbf{s}}}} = D^{{{\textbf{s}}}}({{\textbf{r}}}, {{\textbf{x}}})$. Note that the first equation is inverted and the unknown becomes the differential operator $\nabla _{{{\textbf{x}}}}$ as a function of ${{\textbf{x}}}({{\textbf{r}}}): {{\hat{\Omega }}^{{\textbf{r}}}}\rightarrow {\Omega }$. The system is associated with a global operator comprised of two separate operators, one for each equation

42 $$\begin{aligned} {\mathcal {L}}^{{{\textbf{x}}}, {{\textbf{s}}}}({{\textbf{x}}}, {{\textbf{s}}}, \varvec{\phi }_1, \varvec{\phi }_2, D^{{{\textbf{x}}}}, D^{{{\textbf{s}}}})&= {\mathcal {L}}^{{{\textbf{x}}}}({{\textbf{x}}}, \varvec{\phi }_1, D^{{{\textbf{x}}}}, {{\textbf{s}}}) \nonumber \\&\quad + {\mathcal {L}}^{{{\textbf{s}}}}({{\textbf{s}}}, \varvec{\phi }_2, D^{{{\textbf{s}}}}, {{\textbf{x}}}). \end{aligned}$$

For $D^{{{\textbf{x}}}} = {\mathcal {I}}^{2 \times 2}$, the operator ${\mathcal {L}}^{{{\textbf{x}}}}(\cdot , \, \cdot , \, \cdot , \, \cdot )$ can be based on any of the operators from Sect. 3. For $D^{{\textbf{x}}} \ne {\mathcal {I}}^{2 \times 2}$ we restrict ourselves to the weak-form discretisation. With ${{\textbf{s}}}= {{\textbf{s}}}({{\textbf{r}}})$, the regularised weak-form operator becomes (c.f. equation (37)):

43 $$\begin{aligned}&{\mathcal {L}}^{\text {W}}_{\varepsilon }({{\textbf{x}}}, \varvec{\phi }, D^{{{\textbf{x}}}}, {{\textbf{s}}}) \nonumber \\&\quad = \int \limits _{{{\hat{\Omega }}^{{\textbf{r}}}}}\frac{\left( C(\partial _{{{\textbf{r}}}} {{\textbf{s}}}) \nabla _{{{\textbf{r}}}} \varvec{\phi } \right) \, :\left( Q^T(\partial _{{{\textbf{r}}}} {{\textbf{x}}}, \partial _{{{\textbf{r}}}} {{\textbf{s}}}) D^{{{\textbf{x}}}}({{\textbf{s}}}, {{\textbf{x}}}) \, Q(\partial _{{{\textbf{r}}}} {{\textbf{x}}}, \partial _{{{\textbf{r}}}} {{\textbf{s}}}) \right) }{{\mathcal {R}}_{\varepsilon } \left( \det Q(\partial _{{{\textbf{r}}}} {{\textbf{x}}}, \partial _{{{\textbf{r}}}} {{\textbf{s}}}) \right) } \, \textrm{d} {{\textbf{r}}}, \end{aligned}$$

with

$$\begin{aligned} Q(\partial _{{{\textbf{r}}}} {{\textbf{x}}}, \partial _{{{\textbf{r}}}} {{\textbf{s}}}):= C(\partial _{{{\textbf{r}}}} {{\textbf{x}}}) \left( \nabla _{{{\textbf{r}}}} {{\textbf{s}}}\right) \quad \text {and } C(\, \cdot \,) \text { as in }(21). \end{aligned}$$

The operator corresponding to the second part $\nabla _{{{\textbf{r}}}} \cdot \left( D^{{{\textbf{s}}}} \nabla _{{{\textbf{r}}}} {{\textbf{s}}}\right) = 0$ reads

44 $$\begin{aligned} {\mathcal {L}}^{{{\textbf{s}}}}({{\textbf{s}}}, \varvec{\phi }, D^{{{\textbf{s}}}}, {{\textbf{x}}}) = \int \limits _{{{\hat{\Omega }}^{{\textbf{r}}}}} \partial _{{{\textbf{r}}}} \varvec{\phi } \, :\, ( \partial _{{{\textbf{r}}}} {{\textbf{s}}}\, D^{{{\textbf{s}}}}({{\textbf{r}}}, {{\textbf{x}}})) \, \textrm{d} {{\textbf{r}}}. \end{aligned}$$

We note that the coordinate transformation $\partial _{{\varvec{\xi }}} \rightarrow \partial _{{{\textbf{s}}}({\varvec{\xi }})}$ in the NDF operators from Sect. 3.1 reintroduces the Jacobian determinant $\det \partial _{{\varvec{\xi }}} {{\textbf{s}}}$ in the denominator upon pullback of the equations from ${{\hat{\Omega }}^{{\textbf{r}}}}$ into ${{\hat{\Omega }}}$. As such, an iterative algorithm has to be initialised with a nondegenerate controlmap ${{\textbf{s}}}^0: {{\hat{\Omega }}^{{\textbf{r}}}}\rightarrow {{\hat{\Omega }}^{{\textbf{r}}}}$ when ${{\textbf{x}}}^{{{\textbf{s}}}}: {{\hat{\Omega }}}\rightarrow {\Omega }$ and ${{\textbf{s}}}: {{\hat{\Omega }}^{{\textbf{r}}}}\rightarrow {{\hat{\Omega }}^{{\textbf{r}}}}$ are coupled via $D^{{{\textbf{x}}}}$ or $D^{{{\textbf{s}}}}$. In this case, we recommend basing the scheme on (43) instead. In the classical literature, parametric control via ${{\textbf{s}}}$, rather than through a pullback, is accomplished by introducing additional terms in (13) [34, Chapter 4]. While this formulation enables removing $\partial _{{\varvec{\xi }}} {{\textbf{s}}}$ from the denominator, it is not applicable if ${{\textbf{s}}}\notin H^2({{\hat{\Omega }}}, {\mathbb {R}}^2)$ since it requires second-order derivative information of ${{\textbf{s}}}({\varvec{\xi }})$, making it unsuited for this paper’s use-cases. While it may be possible to reduce the regularity requirements of ${{\textbf{s}}}: {{\hat{\Omega }}^{{\textbf{r}}}}\rightarrow {{\hat{\Omega }}^{{\textbf{r}}}}$ in a way anologous to Sect. 3.1, this is beyond the scope of this paper.

Given a reference controlmap ${{\textbf{r}}}: {{\hat{\Omega }}}\rightarrow {{\hat{\Omega }}^{{\textbf{r}}}}$, the controlmap ${{\textbf{s}}}: {{\hat{\Omega }}^{{\textbf{r}}}}\rightarrow {{\hat{\Omega }}^{{\textbf{r}}}}$ is conveniently built from a push-forward of the same finite-dimensional space ${\mathcal {V}}_h$ used to represent the map ${{\textbf{x}}}_h: {{\hat{\Omega }}}\rightarrow {\Omega }$. As mentioned before, substituting a degenerate intermediate controlmap ${{\textbf{s}}}: {{\hat{\Omega }}^{{\textbf{r}}}}\rightarrow {{\hat{\Omega }}^{{\textbf{r}}}}$, produced by an iterative root-finding algorithm applied to the coupled system, may cause problems due to division by zero. In practice this is avoided by initialising the scheme by the tuple $({{\textbf{x}}}^{{{\textbf{r}}}}, {{\textbf{r}}})$ (i.e., the solution for $D^{{{\textbf{x}}}} = D^{{{\textbf{s}}}} = {\mathcal {I}}^{2 \times 2}$ over the reference controlmap ${{\textbf{r}}}: {{\hat{\Omega }}}\rightarrow {{\hat{\Omega }}^{{\textbf{r}}}}$) which is computed using one of the NDF-discretisation from Sect. 3.1. The barrier term in (43) then prevents intermediate iterates $({{\textbf{x}}}^{{{\textbf{s}}}}, {{\textbf{s}}})^i$ from leaving the set of nondegenerate maps. As before, a discretisation takes the test functions $(\varvec{\phi }_1, \varvec{\phi }_2)$ from the finite-dimensional space ${\mathcal {U}}_h^{{\textbf{0}}} \times {\mathcal {U}}_h^{{\textbf{0}}}$ and finds the root using Newton’s method. In practice, the coupled scheme converges reliably for a wide range of diffusivities $D^{{\textbf{x}}}, D^{{{\textbf{s}}}}$ when initialised with $({{\textbf{x}}}^{{{\textbf{r}}}}, {{\textbf{r}}})$.

Depending on the choice of $D^{{{\textbf{x}}}}$ and $D^{{{\textbf{s}}}}$, the solution of the coupled system may no longer be uniformly nondegenerate (even for boundary correspondences that lead to UNDG maps for $D = {\mathcal {I}}^{2\times 2}$). To avoid singularities, we introduce a general stabilisation on the patch vertices. As such, let $\Gamma ^v = \{ {\textbf{v}}^1, \ldots , {\textbf{v}}^{N_v} \} \subset {\overline{{{\hat{\Omega }}}}}$ be the set of patch vertices shared by at least two patches.

The singularities are avoided by introducing an appropriate regularisation. For this purpose, we introduce the Gaussian blending functions

45 $$\begin{aligned} g_i^{\kappa }({{\textbf{r}}})&:= A_i \exp {\left( -\left( \frac{\kappa }{d_i^{\text {min}}} \Vert {{\textbf{r}}}- {{\textbf{r}}}({\textbf{v}}^i) \Vert \right) ^2 \right) }, \quad \text {with} \quad \nonumber \\ d_i^{\min }&:= \min \left\{ \Vert {\textbf{v}}^i - {\textbf{v}}^j \Vert \, \, \big | \, \, j \in \{1, \ldots , N_p\} {\setminus } \{i\} \right\} \end{aligned}$$

and $\kappa > 0$. Here, the $A_i$ are chosen such that

$$\begin{aligned} \forall {\textbf{v}}^i \in \Gamma ^v: \quad \sum _{i=1}^{N_v} g_i^{\kappa }({{\textbf{r}}}({\textbf{v}}^i)) = 1. \end{aligned}$$

Let *D* be the diffusivity in question and let ${\mathcal {D}}_i = \{D_i^1, \ldots , D_i^q \}$ be the set containing the limits

$$\begin{aligned} D_i^j:= \lim \limits _{{\varvec{\xi }}\rightarrow {\textbf{v}}^i} D({{\textbf{r}}}({\varvec{\xi }})) \quad \text { s.t. } {\varvec{\xi }}\in {{\hat{\Omega }}}_j \quad \text {for each patch } {{\hat{\Omega }}}_j \text { with } {\overline{{{\hat{\Omega }}}}}_j \cap \{{\textbf{v}}^i\} = \{ {\textbf{v}}^i \}. \end{aligned}$$

We define ${\overline{D}}_i$ as the average of the $D_i^j \in {\mathcal {D}}_i$, i.e.,

46 $$\begin{aligned} {\overline{D}}_i := \sum \limits _{D_i^j \in {\mathcal {D}}_i} D_i^j. \end{aligned}$$

The regularisation ensures that the regularised ${\overline{D}}^{\kappa }(D) \in \text {SPD}^{2 \times 2}({{\hat{\Omega }}^{{\textbf{r}}}})$ is single-valued in the ${{\textbf{r}}}({\textbf{v}}^i)$ by replacing

47 $$\begin{aligned} D \rightarrow {\overline{D}}^{\kappa }(D) := \left( 1 - \sum \limits _{i=1}^{N_v} g_i^{\kappa } \right) D + \sum _{i=1}^{N_v} g_i^{\kappa } {\overline{D}}_i. \end{aligned}$$

The decay rate $\kappa > 0$ in (45) tunes the degree of regularisation and is relatively insensitive to the characteristic length-scale of $\Omega ^{{{\textbf{r}}}}$ thanks to the scaling by $d_i^{\min }$. It should be noted that other regularisations exist besides (46).

### Patch interface removal

The images of local (in ${\Omega }^{\square }$) isolines under the ${{\textbf{x}}}^{{{\textbf{r}}}}({\varvec{\xi }}) \circ {{\textbf{m}}}^i$ will generally form a (possibly steep) angle across patch interfaces when joined together on ${\Omega }$. In certain applications, it may be desirable to decrease or largely remove the steep interface angles. As ${{\textbf{x}}}: {{\hat{\Omega }}^{{\textbf{r}}}}\rightarrow {\Omega }$ is diffeomorphic in ${{\hat{\Omega }}^{{\textbf{r}}}}$, a controlmap ${{\textbf{s}}}: {{\hat{\Omega }}^{{\textbf{r}}}}\rightarrow {{\hat{\Omega }}^{{\textbf{r}}}}$ that removes steep angles will, by extension, remove them in the recomputed map ${{\textbf{x}}}^{{{\textbf{s}}}}: {{\hat{\Omega }}}\rightarrow {\Omega }$. As such, interface removal can be regarded as an *a priori* step since it requires no prior knowledge of ${{\textbf{x}}}: {{\hat{\Omega }}}\rightarrow {\Omega }$.

We would like to accomplish

48 $$\begin{aligned} \forall \gamma _{jk} \in \Gamma ^I: \quad [\![ (\partial _{{\varvec{\mu }}^{\perp }} {{\textbf{s}}}({{\textbf{r}}}))]\!] = 0 \quad \text {on } {{\textbf{r}}}(\gamma _{jk}), \end{aligned}$$

wherein $[\![ \, \cdot \, ]\!]$ now denotes the ordinary entry-wise jump term while $\partial _{{\varvec{\mu }}^{\perp }}$ denotes the directional derivative transversal to ${{\textbf{r}}}(\gamma _{ij})$ (i.e., either $\partial _{\mu _1}$ or $\partial _{\mu _2}$ on ${{\textbf{r}}}(\gamma _{jk}^+)$ and ${{\textbf{r}}}(\gamma _{jk}^-)$). Requirement (48) can be weakly enforced by utilising in (41) the diffusivity

49 $$\begin{aligned} D^{{{\textbf{s}}}}({{\textbf{r}}}, {{\textbf{x}}}) = D^{{{\textbf{s}}}}_{\Gamma ^I}({{\textbf{r}}}) := \partial _{\mu _1} {{\textbf{r}}}\otimes \partial _{\mu _1} {{\textbf{r}}}+ \partial _{\mu _2} {{\textbf{r}}}\otimes \partial _{\mu _2} {{\textbf{r}}}\quad \text {on} \quad {{\textbf{r}}}({\hat{\Omega }}_i). \end{aligned}$$

Meanwhile, if $D^{{{\textbf{x}}}} = {\mathcal {I}}^{2 \times 2}$, (41) is decoupled and the map ${{\textbf{x}}}({\varvec{\xi }}): {{\hat{\Omega }}}\rightarrow {\Omega }$ can be computed from a degenerate initial guess using an NDF-discretisation. The diffusivity from (49) urges ${{\textbf{s}}}: {{\hat{\Omega }}^{{\textbf{r}}}}\rightarrow {{\hat{\Omega }}^{{\textbf{r}}}}$ to map the patchwise isolines ${{\textbf{s}}}({\varvec{\mu }})$ smoothly across patch interfaces. As the magnitude of $\partial _{\mu _i} {{\textbf{r}}}$ depends on ${{\textbf{r}}}\circ {{\textbf{m}}}^i: {\Omega }^{\square } \rightarrow {{\textbf{r}}}({{\hat{\Omega }}}_i)$ (and may therefore be subject to considerable changes between patches), the best results are obtained by a normalisation, i.e.,

50 $$\begin{aligned} D^{{{\textbf{s}}}}({{\textbf{r}}}, {{\textbf{x}}})&= {\widehat{D}}^{{{\textbf{s}}}}_{\Gamma ^I}({{\textbf{r}}}) := {\widehat{\partial }}_{\mu _1} {{\textbf{r}}}\otimes {\widehat{\partial }}_{\mu _1} {{\textbf{r}}}\nonumber \\&\quad + {\widehat{\partial }}_{\mu _2} {{\textbf{r}}}\otimes {\widehat{\partial }}_{\mu _2} {{\textbf{r}}}\quad \text {on} \quad {{\textbf{r}}}({\hat{\Omega }}_i), \quad \text {with } {\widehat{\partial }}_{\mu _i} {{\textbf{r}}}:= \frac{1}{\left\| \partial _{\varvec{\mu _i}} {{\textbf{r}}}\right\| } \partial _{\varvec{\mu _i}} {{\textbf{r}}}. \end{aligned}$$

Note that

$$\begin{aligned} {\text {tr}}({\mathcal {I}}^{2 \times 2}) = {\text {tr}}\left( {\widehat{D}}^{{{\textbf{s}}}}_{\Gamma ^I}({{\textbf{r}}}) \right) = 2. \end{aligned}$$

**Fig. 11:**
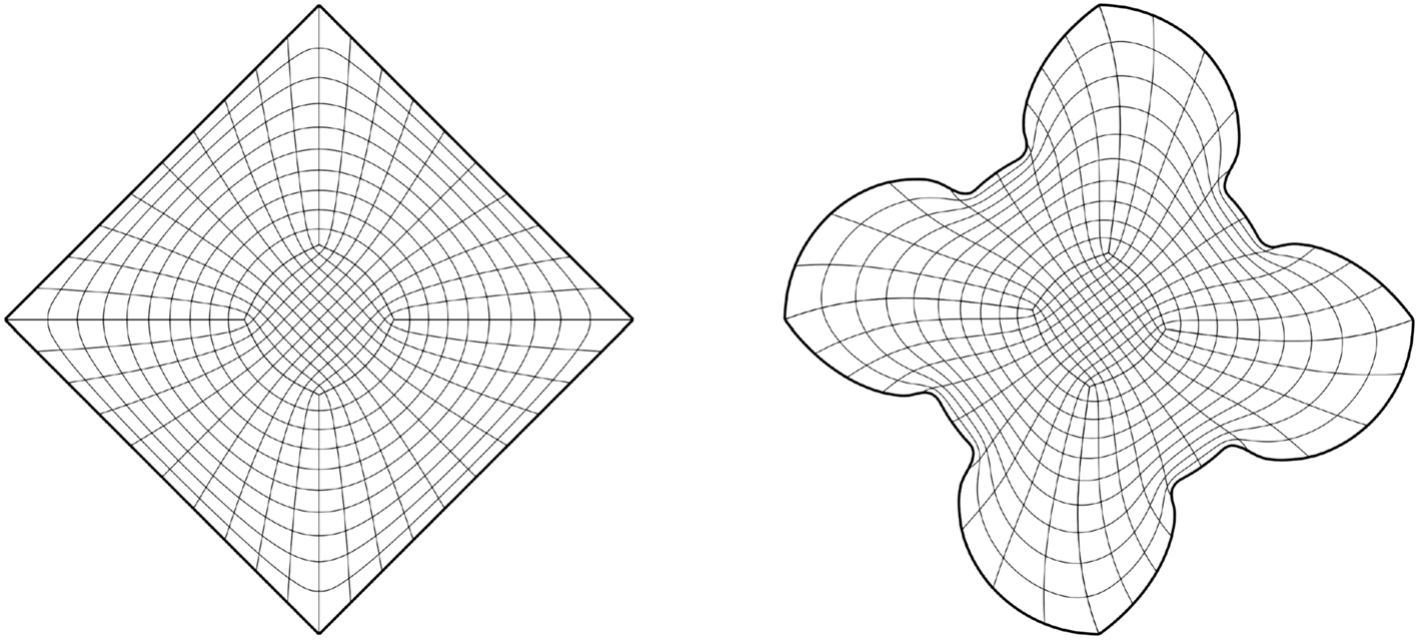
The screw geometry after suppression of local transverse gradient jumps

The normalisation has a similar effect as minimising (48) while now suppressing jumps in the normalised transversal component of the ${{\textbf{r}}}(\gamma _{jk})$ with $\gamma _{jk} \in \Gamma ^I$, i.e., we are penalising jumps in the transverse direction but not the direction’s magnitudes. We are considering the screw geometry from Fig. 8b and perform interface removal with normalisation. Figure 11 shows the resulting reparameterisation. As a measure of the degree of interface removal, we utilise the following value

51 $$\begin{aligned} L_{\Gamma }^2({{\textbf{x}}}) = \sum \limits _{\gamma _{jk} \in \Gamma ^I} \int \limits _{{{\textbf{r}}}(\gamma _{jk})} \left\| [\![ {\widehat{\partial }}_{{\varvec{\mu }}^{\perp }} {{\textbf{x}}}({{\textbf{r}}})]\!] \right\| ^2 \textrm{d} \Gamma , \end{aligned}$$

where ${\widehat{\partial }}_{{\varvec{\mu }}^{\perp }}$ denotes the normalised directional derivative transverse to ${{\textbf{r}}}(\gamma _{jk})$. With

$$\begin{aligned} \frac{L_{\Gamma }({{\textbf{x}}}_h^{{{\textbf{s}}}})}{L_{\Gamma }({{\textbf{x}}}_h)} \approx 0.0998 \end{aligned}$$

the technique is highly effective.

As stated in Theorem 2, methods based on (41) do not exclude singularities in ${{\textbf{s}}}: {{\hat{\Omega }}^{{\textbf{r}}}}\rightarrow {{\hat{\Omega }}}^{{{\textbf{s}}}}$. In [43] it is proved that parametric smoothness across interfaces necessarily creates singularities on vertices ${\textbf{v}} \in {{\hat{\Omega }}^{{\textbf{r}}}}$ with ${\text {valence}}({\textbf{v}}) \ne 4$. While singularities are in practice avoided by discrete approximations (and can therefore often be ignored in practice), for merely essentially bounded $D^{{{\textbf{s}}}} \in \text {SPD}^{2 \times 2}({{\hat{\Omega }}^{{\textbf{r}}}})$, we may expect

$$\begin{aligned} \inf \limits _{{{\hat{\Omega }}^{{\textbf{r}}}}} \det \partial _{{{\textbf{r}}}} {{\textbf{s}}}\rightarrow 0 \quad \text {or} \quad \sup \limits _{{{\hat{\Omega }}^{{\textbf{r}}}}} \det \partial _{{{\textbf{r}}}} {{\textbf{s}}}\rightarrow \infty \end{aligned}$$

in a refinement study. Clearly, one way of handling singularities is seeking the controlmap as the solution of an associated time-stepping scheme and terminating the recursion before it reaches the steady-state solution. A termination criterion could then be based on the value of $\det \partial _{{{\textbf{r}}}} {{\textbf{s}}}$ in the vertices.

**Fig. 12:**
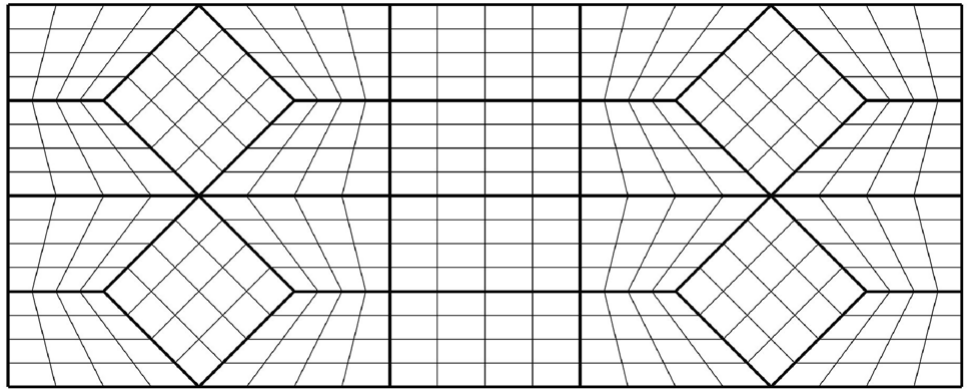
Bilinear multipatch covering of a rectangular domain comprised of 24 patches

**Fig. 13:**
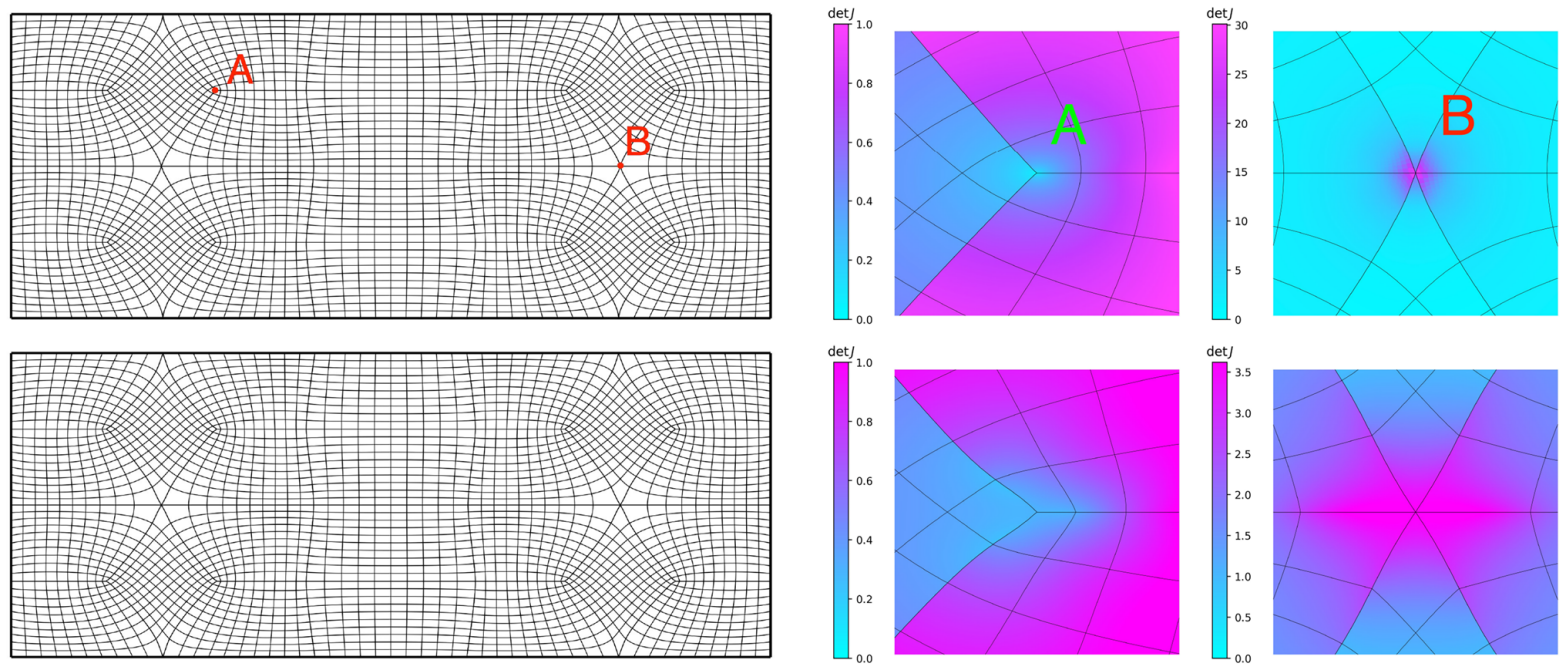
Plot of the controlmaps ${{\textbf{s}}}: {{\hat{\Omega }}}\rightarrow {{\hat{\Omega }}}$ (top) and ${{\textbf{s}}}^{\text {reg}}: {{\hat{\Omega }}}\rightarrow {{\hat{\Omega }}}$ (bottom) along with a zoom-in onto vertices in which the Jacobian determinant grows/shrinks unboundedly in the absence of regularisation

Another possibility is employing the stabilisation from (47). We are considering the rectangular parametric domain ${{\hat{\Omega }}}= {{\hat{\Omega }}^{{\textbf{r}}}}$ along with an irregular multipatch covering depicted in Fig. 12. We perform a refinement study of (normalised) interface removal with and without regularisation, initially assigning a uniform cubic knotvector with three internal knots to each side $L_i \in \Gamma ^B$ and facet $\gamma _{ij} \in \Gamma ^I$. Each refinement $h \rightarrow h/2$ halves the knotvector’s knotspans. We are monitoring the value of $\nu _{\Gamma }( \, \cdot \,):= L_{\Gamma }(\, \cdot \,) / L_{\Gamma }({{\textbf{r}}})$ for the original ${{\textbf{s}}}: {{\hat{\Omega }}^{{\textbf{r}}}}\rightarrow {{\hat{\Omega }}^{{\textbf{r}}}}$ and its regularised counterpart ${{\textbf{s}}}^{\text {reg}}: {{\hat{\Omega }}^{{\textbf{r}}}}\rightarrow {{\hat{\Omega }}^{{\textbf{r}}}}$, as well as the values $\min \det J_{{\textbf{v}}}(\, \cdot \,)$ and $\max \det J_{{\textbf{v}}}(\, \cdot \,)$ which are the minimum and maximum values of $\det \partial _{{{\textbf{r}}}}( \, \cdot \, )$ over all patch vertices ${{\textbf{r}}}({\textbf{v}}^i)$. As $\det \partial _{{{\textbf{r}}}}(\, \cdot \,)$ is not single-valued in the ${\textbf{v}}^i \in \Gamma ^v$, we define this value as the minimum / maximum of taking the limit on each adjacent patch. Table 3 contains the reference values in the absence of regularisation while Table 4 contains the corresponding values for the regularisation $D^{{{\textbf{s}}}} \rightarrow {\overline{D}}^{\kappa }(D^{{{\textbf{s}}}})$ with $\kappa = 9$. Furthermore, Fig. 13 shows the controlmap with and without regularisation after the last refinement level. Table 3 clearly demonstrates that $\min \det J_{{\textbf{v}}}(\, \cdot \,)$ and $\max \det J_{{\textbf{v}}}(\, \cdot \,)$ shrink / grow unboundedly in the absence of regularisation while Table 4 demonstrates that regularisation prevents further shrinkage / growth under refinement.

**Table 3:** Reference values of performing interface removal on the quadrangulation from Fig. 12 in the absence of regularisation

	h	h/2	h/4	h/8	h/16
$\nu _{\Gamma }({{\textbf{s}}})$	0.273	0.197	0.162	0.140	0.0991
$\min \det J_{{\textbf{v}}}({{\textbf{s}}})$	0.0679	0.0466	0.0357	0.0294	0.0184
$\max \det J_{{\textbf{v}}}({{\textbf{s}}})$	7.46	11.9	15.6	18.9	30.1

**Table 4:** The outcomes of performing regularised interface removal with $\kappa = 9$

$\kappa =9$	h	h/2	h/4	h/8	h/16
$\nu _{\Gamma }({{\textbf{s}}}^{\text {reg}})$	0.426	0.413	0.407	0.406	0.406
$\min \det J_{{\textbf{v}}}({{\textbf{s}}}^{\text {reg}})$	0.223	0.262	0.251	0.249	0.248
$\max \det J_{{\textbf{v}}}({{\textbf{s}}}^{\text {reg}})$	4.44	3.93	3.84	3.86	3.84

As expected, the regularisation also prevents monotone decrease of $\nu _{\Gamma }({{\textbf{s}}}^{\text {reg}})$, eventually settling for a value of $\sim 0.4 L_{\Gamma }({{\textbf{r}}})$ relative to the reference value of Fig. 12. Meanwhile, the corresponding value shrinks unboundedly in the absence of regularisation.

For larger values of $\kappa$, we expect the discretisation to settle for a lower value of $\nu _{\Gamma }({{\textbf{s}}}^{\text {reg}})$ at the expense of reducing / increasing the values of $\min \det J_{{\textbf{v}}}({{\textbf{s}}}^{\text {reg}})$ and $\max \det J_{{\textbf{v}}}({{\textbf{s}}}^{\text {reg}})$. Table 5 contains the outcomes for regularisation with $\kappa = 18$.

**Table 5:** The outcomes of performing regularised interface removal with $\kappa = 18$

$\kappa =18$	h	h/2	h/4	h/8	h/16
$\nu _{\Gamma }({{\textbf{s}}}^{\text {reg}})$	0.315	0.314	0.309	0.303	0.299
$\min \det J_{{\textbf{v}}}({{\textbf{s}}}^{\text {reg}})$	0.0892	0.143	0.179	0.174	0.167
$\max \det J_{{\textbf{v}}}({{\textbf{s}}}^{\text {reg}})$	6.36	6.98	6.33	6.16	6.08

Indeed, the table confirms this expectation, settling for a value of $\sim 0.3 L_{\Gamma }({{\textbf{r}}})$ while roughly doubling the shrinkage / growth of $\det \partial _{{{\textbf{r}}}}({{\textbf{s}}}^{\text {reg}})$ compared to $\kappa = 9$.

We conclude that the proposed regularisation is an effective means to tune the degree of interface removal at the expense of cell size homogeneity reduction.

### Cell size homogenisation

Unless a PDE problem is known to benefit from a parameterisation with a tighter clustering of grid lines in specific regions (such as boundary layers in flow problems), parameterisation quality criteria are often based on some measure of the cell size homogeneity. For this, a popular measure is the *Area* functional

52 $$\begin{aligned} L_{\text {Area}}({{\textbf{x}}}) = \int \limits _{{{\hat{\Omega }}}} \left( \det J({{\textbf{x}}}) \right) ^2 \textrm{d} {\varvec{\xi }}, \end{aligned}$$

which measures the variance of $\det J({{\textbf{x}}})$ over ${{\hat{\Omega }}}$, with smaller values indicating better homogeneity. In the multipatch setting, it is more natural to measure the homogeneity on each individual patch and summing over all patches

53 $$\begin{aligned} L_{\text {Area}}({{\textbf{x}}}) := \sum _{i=1}^{N_p} \int \limits _{{\Omega }^{\square }} \left( \det \partial _{{\varvec{\mu }}} ({{\textbf{x}}}({\varvec{\xi }}) \circ {{\textbf{m}}}^i) ({\varvec{\mu }}) \right) ^2 \textrm{d} {\varvec{\mu }}, \end{aligned}$$

wherein ${\varvec{\mu }}= (\mu _1, \mu _2)^T$ denotes the free coordinate functions in ${\Omega }^{\square }$. Direct minimisation of (53) over the map’s inner controlpoints leads to a nonconvex problem which is furthermore prone to yielding degenerate maps.

In the context of the coupled system (41), there are two main ways to achieve homogenisation without having to resort to nonconvex optimisation:

1. Designing a diffusivity $D^{{{\textbf{s}}}}({{\textbf{r}}}, {{\textbf{x}}}^{{{\textbf{s}}}})$ that contracts / expands the cell sizes of ${{\textbf{s}}}: {{\hat{\Omega }}}^{{{\textbf{r}}}} \rightarrow {{\hat{\Omega }}}^{{{\textbf{r}}}}$ wherever the cell sizes of ${{\textbf{x}}}^{{{\textbf{s}}}}: {{\hat{\Omega }}}^{{{\textbf{r}}}} \rightarrow {\Omega }$ are large / small, while taking $D^{{{\textbf{x}}}}({{\textbf{s}}}, {{\textbf{x}}}^{{{\textbf{s}}}}) = {\mathcal {I}}^{2 \times 2}$.
2. Picking $D^{{{\textbf{s}}}} = D^{{{\textbf{s}}}}({{\textbf{r}}})$ (i.e., $D^{{{\textbf{s}}}}$ has no dependency on ${{\textbf{x}}}^{{{\textbf{s}}}}$) while designing a diffusivity $D^{{{\textbf{x}}}} = D^{{{\textbf{x}}}}({{\textbf{x}}}^{{{\textbf{s}}}})$ that encourages cell size homogenisation.

As for method 1., we notice that for $D^{{{\textbf{x}}}} = {\mathcal {I}}^{2 \times 2}$, the solution of the inverse Laplace problem is merely a property of the shapes ${{\hat{\Omega }}^{{\textbf{r}}}}$ and ${\Omega }$ as well as the diffeomorphic boundary correspondence ${\textbf{F}}^{{{\textbf{r}}}\rightarrow {{\textbf{x}}}}: \partial {{\hat{\Omega }}^{{\textbf{r}}}}\rightarrow \partial {\Omega }$. As such, a controlmap ${{\textbf{s}}}: {{\hat{\Omega }}^{{\textbf{r}}}}\rightarrow {{\hat{\Omega }}^{{\textbf{r}}}}$ that is the identity on $\partial {{\hat{\Omega }}^{{\textbf{r}}}}$ computes the composition ${{\textbf{x}}}^{{{\textbf{s}}}}({{\textbf{r}}}) = {{\textbf{x}}}^{{{\textbf{r}}}} \circ {{\textbf{s}}}({{\textbf{r}}})$. Therefore, we may require ${{\textbf{s}}}({{\textbf{r}}})$ to contract cells in ${{\textbf{r}}}({{\hat{\Omega }}}_i)$ wherever $\det \partial _{{\varvec{\mu }}} {{\textbf{x}}}^{{{\textbf{s}}}}({{\textbf{r}}})$ is large and vice versa. We may also choose to penalise based on $\partial _{{\varvec{\xi }}} {{\textbf{x}}}^{{{\textbf{s}}}}({{\textbf{r}}})$ or $\partial _{{{\textbf{r}}}} {{\textbf{x}}}^{{{\textbf{s}}}}({{\textbf{r}}})$ to reduce the Area functional in a different coordinate system if desired.

To cast this problem into the form of (42), we contract the cells of ${{\textbf{s}}}({{\textbf{r}}})$ by penalising the value of ${\text {tr}}(G^{{{\textbf{r}}}\rightarrow {{\textbf{s}}}})$, where $G^{{{\textbf{r}}}\rightarrow {{\textbf{s}}}}$ denotes the metric between the coordinate systems induced by ${{\textbf{r}}}: {{\hat{\Omega }}}\rightarrow {{\hat{\Omega }}}^{{{\textbf{r}}}}$ and ${{\textbf{s}}}: {{\hat{\Omega }}}^{{{\textbf{r}}}} \rightarrow {{\hat{\Omega }}}^{{{\textbf{r}}}}$. This is accomplished by introducing a monitor function $\sigma ({{\textbf{x}}}^{{{\textbf{s}}}})$ that assumes large values in regions where contraction is desired and vice-versa. For instance

$$\begin{aligned} \sigma ^k({{\textbf{x}}}^{{{\textbf{s}}}})({{\textbf{r}}}) = (\det \partial _{{\varvec{\mu }}} {{\textbf{x}}}^{{{\textbf{s}}}}({{\textbf{r}}}))^k \quad \text {on} \quad {{\textbf{r}}}({{\hat{\Omega }}}_i). \end{aligned}$$

The controlmap is then sought as the minimizer of the following quadratic Dirichlet energy term:

54 $$\begin{aligned} \min \limits _{{{\textbf{s}}}: {{\hat{\Omega }}}^{{{\textbf{r}}}} \rightarrow {{\hat{\Omega }}}^{{{\textbf{r}}}}} \int \limits _{{{\hat{\Omega }}}^{{{\textbf{r}}}}} \sigma ^k \left( {{\textbf{x}}}^{{{\textbf{s}}}} \right) {\text {tr}}\left( G^{{{\textbf{r}}}\rightarrow {{\textbf{s}}}} \right) \textrm{d} {{\textbf{r}}}, \quad \text {s.t.} \quad {{\textbf{s}}}({{\textbf{r}}}) = {{\textbf{r}}}\text { on } \partial {{\hat{\Omega }}}^{{{\textbf{r}}}}, \end{aligned}$$

where larger values of $k > 0$ lead to a more drastic homogenisation. From the Euler-Lagrange equations of (54), it follows that we are solving the coupled system (42) with

55 $$\begin{aligned} D^{{{\textbf{x}}}} = {\mathcal {I}}^{2 \times 2} \quad \text {and} \quad D^{{{\textbf{s}}}}({{\textbf{x}}}^{{{\textbf{s}}}}) = \sigma ^k({{\textbf{x}}}^{{{\textbf{s}}}}) {\mathcal {I}}^{2 \times 2}. \end{aligned}$$

The contraction under $\sigma ^k({{\textbf{x}}})$ has a similar effect as operating on $(\det \partial _{{{\textbf{r}}}} {{\textbf{s}}})^2$ directly while being inherently less prone to yielding degenerate discrete maps.

While a formal proof is lacking, it is plausible to assume that the coupled system is well-posed under this choice of $D^{{{\textbf{s}}}}$ since for any bijective ${{\textbf{s}}}: {{\hat{\Omega }}}^{{{\textbf{r}}}} \rightarrow {{\hat{\Omega }}}^{{{\textbf{r}}}}$ (and given a diffeomorphic boundary correspondence ${\textbf{F}}^{{{\textbf{r}}}\rightarrow {{\textbf{x}}}}$), the coupled system (42) approximates a UNDG map ${{\textbf{x}}}^{{{\textbf{s}}}}: {{\hat{\Omega }}}^{{{\textbf{r}}}} \rightarrow {\Omega }$ that satisfies $\sigma ^k({{\textbf{x}}}^{{{\textbf{s}}}}) > 0$ (a.e.) such that $D^{{{\textbf{s}}}}({{\textbf{x}}}^{{{\textbf{s}}}}) \in \text {SPD}^{2 \times 2}({{\hat{\Omega }}}^{{{\textbf{r}}}})$. However, a root-finding algorithm may diverge in case the Newton increment accidentally causes ${{\textbf{x}}}^{{{\textbf{s}}}}$ to leave the set of UNDG maps. In practice, this is avoided by the barrier property of (43) and the scheme converges reliably using Newton’s method with line search for a wide range of choices $k > 0$ when the scheme is initialised with the tuple $({{\textbf{x}}}^{{{\textbf{r}}}}, {{\textbf{r}}})$ (i.e., the reference solution and reference controlmap).

**Fig. 14:**
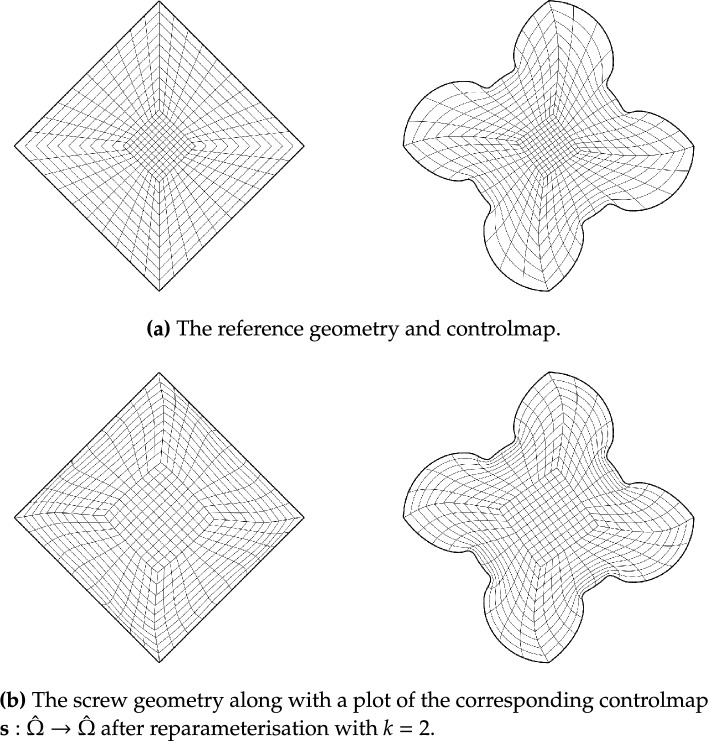
The screw geometry before and after reparameterisation under (55) with $k=2$

We are again considering the screw geometry depicted with the bilinearly covered parametric domain before reparameterisation in Fig. 14a. Here, the reference solution corresponds to ${{\textbf{r}}}({\varvec{\xi }}) = {\varvec{\xi }}$. Figure 14b shows the geometry along with the associated controlmap ${{\textbf{s}}}({\varvec{\xi }})$ after reparameterisation with $k=2$. Denoting the reparameterised maps by ${{\textbf{x}}}_h^k$, we define

56 $$\begin{aligned} \nu _{\text {Area}}^k := \frac{L_{\text {Area}}({{\textbf{x}}}_h^k)}{L_{\text {Area}}({{\textbf{x}}}_h^0)} \quad \text {and} \quad \nu _{\det J}^k := \frac{\sup \partial _{{\varvec{\mu }}} {{\textbf{x}}}_h^k}{\inf \partial _{{\varvec{\mu }}} {{\textbf{x}}}_h^k}, \end{aligned}$$

where the latter is approximated by sampling over the abscissae of a dense Gauss-Legendre quadrature scheme.

**Table 6:** Table showing the ratios between evaluating (53) in ${{\textbf{x}}}^k_h$ and the reference evaluation in ${{\textbf{x}}}_h^0$ for various values of *k* as well as the ratio of the maximum and minimum values of $\det \partial _{{\varvec{\mu }}} {{\textbf{x}}}_h^k$ sampled over a dense quadrature scheme

k	0	1	2	3
$\nu _{\text {Area}}^k$	1	0.749	0.680	0.649
$\nu _{\det J}^k$	23.3	6.67	4.22	5.69

Table 6 contains the values of $\nu _{\text {Area}}^k$ and $\nu _{\det J}^k$ for various values of $k \in [0, 3]$. The table clearly demonstrates that the methodology has the desired effect, reaching saturation for larger values of *k*. Furthermore, all parameterisations with $k > 0$ significantly reduce the anisotropy of $\det \partial _{{\varvec{\mu }}} {{\textbf{x}}}_h^k$. We mention that the diffusivity is merely essentially bounded since $\sigma ^{k}({{\textbf{x}}}^{{{\textbf{s}}}})$ is generally patchwise discontinuous. However, the problem requires no stabilisation in practice. A possible explanation is that cell size homogenisation counteracts the tendency to generate singularities for discrete approximations.

**Fig. 15:**
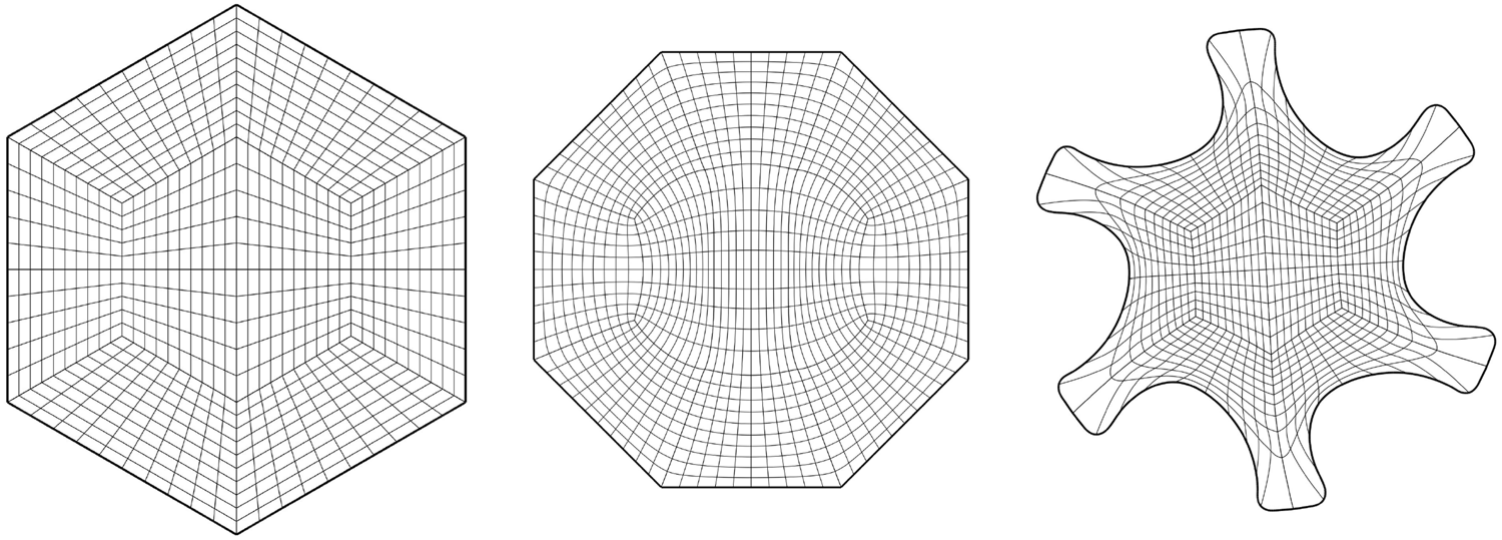
Depiction of ${{\hat{\Omega }}}$, the parameterisation of ${{\hat{\Omega }}^{{\textbf{r}}}}$ under ${\widehat{{{\textbf{r}}}}}: {{\hat{\Omega }}}\rightarrow {{\hat{\Omega }}^{{\textbf{r}}}}$ and the reference parameterisation of ${\Omega }$ under ${{\textbf{x}}}^{{\widehat{{{\textbf{r}}}}}}({\varvec{\xi }}): {{\hat{\Omega }}}\rightarrow {{\hat{\Omega }}^{{\textbf{r}}}}$

**Fig. 16:**
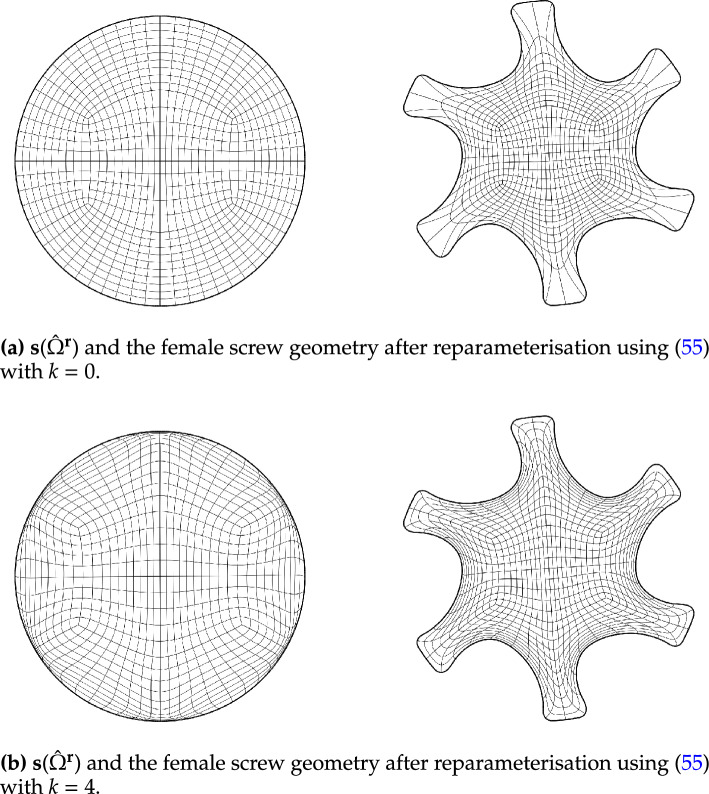
The female screw geometry after reparameterisation under (55) with $k=0$ and $k=4$

As a second example, we are considering the geometry depicted in Fig. 15 (right) along with the parametric domain ${{\hat{\Omega }}}$ given by a regular six-sided polygon. Unlike the geometry from Fig. 14a, this geometry has no corners. As such, we take ${{\hat{\Omega }}^{{\textbf{r}}}}$ to be the unit disc where the boundary correspondence ${\textbf{F}}^{{\varvec{\xi }}\rightarrow {{\textbf{r}}}}: \partial {{\hat{\Omega }}}\rightarrow \partial {{\hat{\Omega }}^{{\textbf{r}}}}$ is chosen such that the induced correspondence ${\textbf{F}}^{{{\textbf{r}}}\rightarrow {{\textbf{x}}}}: \partial {{\hat{\Omega }}^{{\textbf{r}}}}\rightarrow \partial {\Omega }$ is diffeomorphic. The interior of ${{\hat{\Omega }}^{{\textbf{r}}}}$ is parameterised by applying the bilinearly-blended Coons’ patch approach to each patch of ${{\hat{\Omega }}}$ individually. The reference parameterisation ${{\textbf{x}}}^{{\widehat{{{\textbf{r}}}}}}({\varvec{\xi }}): {{\hat{\Omega }}}\rightarrow {\Omega }$ and the associated reference controlmap ${\widehat{{{\textbf{r}}}}}: {{\hat{\Omega }}}\rightarrow {{\hat{\Omega }}^{{\textbf{r}}}}$ are depicted in Fig. 15. In this example, we are combining the techniques from this section with those from Sect. 4.2. As an a priori step, we substitute the reference parameterisation ${\widehat{{{\textbf{r}}}}}: {{\hat{\Omega }}}\rightarrow {{\hat{\Omega }}^{{\textbf{r}}}}$ in the diffusivity from (50) and solve the decoupled system (41) with this choice of $D^{{{\textbf{s}}}}$ for the controlmap that maps ${{\hat{\Omega }}^{{\textbf{r}}}}$ onto itself. This results in the new reference controlmap ${{\textbf{r}}}({\varvec{\xi }}): {{\hat{\Omega }}}\rightarrow {{\hat{\Omega }}^{{\textbf{r}}}}$ that largely removes the patch interfaces. The new parameterisation of ${{\hat{\Omega }}^{{\textbf{r}}}}$ and the associated new reference map ${{\textbf{x}}}^{{{\textbf{r}}}}: {{\hat{\Omega }}}\rightarrow {{\hat{\Omega }}^{{\textbf{r}}}}$ are depicted in Fig. 16a. Here, we do not stabilise using (47) since the discrete approximation remains uniformly nondegenerate with acceptable behaviour in the vicinity of the patch vertices.

**Table 7:** Table showing the ratios between evaluating (53) in ${{\textbf{x}}}^k_h$ and the reference evaluation in ${{\textbf{x}}}_h^0$ for various values of *k*

k	0	1	2	3	4
$\nu _{\text {Area}}^k$	1.02	0.496	0.414	0.386	0.373
$\nu _{\Gamma }^k$	0.117	0.142	0.158	0.171	0.180

Table 7 contains the values of

$$\begin{aligned} \nu ^k_{\text {Area}}&:= \frac{L_{\text {Area}}({{\textbf{x}}}^k_h)}{L_{\text {Area}}({{\textbf{x}}}^{{\widehat{{{\textbf{r}}}}}}_h)} \quad \text {and} \quad \\ \nu ^k_{\Gamma }&:= \frac{L_{\Gamma }({{\textbf{x}}}_h^k)}{L_{\Gamma }({{\textbf{x}}}^{{\widehat{{{\textbf{r}}}}}}_h)}, \quad \text {with } L_{\Gamma }(\, \cdot \,) \text { as in }(51), \end{aligned}$$

for various $k \in [0, 4]$. Figure 16 shows the parameterisation of the geometry for $k = 0$ and $k=4$. The table clearly demonstrates a monotonous reduction of $\nu ^k_{\text {Area}}$ (eventually reaching saturation for larger values of *k*) at the expense of a slight increase in the value of $\nu ^k_{\Gamma }$.

For more precise control over the expansion / contraction of cells, it can be helpful to decompose the diffusivity into the scaling $\sigma ^k({{\textbf{x}}}^{{{\textbf{s}}}})$ times the sum of two symmetric rank one tensors, i.e.,

57 $$\begin{aligned} D({{\textbf{x}}}^{{{\textbf{s}}}})&= \frac{2}{a_i + 1} \sigma ^k({{\textbf{x}}}^{{{\textbf{s}}}}) \left( a_i \hat{{\textbf{v}}}^{i, 1} \otimes \hat{{\textbf{v}}}^{i, 1} \right. \nonumber \\&\left. \quad + \hat{{\textbf{v}}}^{i, 2} \otimes \hat{{\textbf{v}}}^{i, 2} \right) , \quad \text { on } {{\textbf{r}}}({\hat{\Omega }}^i), \quad \text {where} \quad a_i > 0. \end{aligned}$$

Here, $\hat{{\textbf{v}}}^{i, 1}$ and $\hat{{\textbf{v}}}^{i, 2}$ have length one and are not parallel. Note that

$$\begin{aligned} {\text {tr}}\left( \frac{2}{a_i + 1}\left( a_i \hat{{\textbf{v}}}^{i, 1} \otimes \hat{{\textbf{v}}}^{i, 1} + \hat{{\textbf{v}}}^{i, 2} \otimes \hat{{\textbf{v}}}^{i, 2} \right) \right) = 2, \quad \text {as before}. \end{aligned}$$

**Fig. 17:**
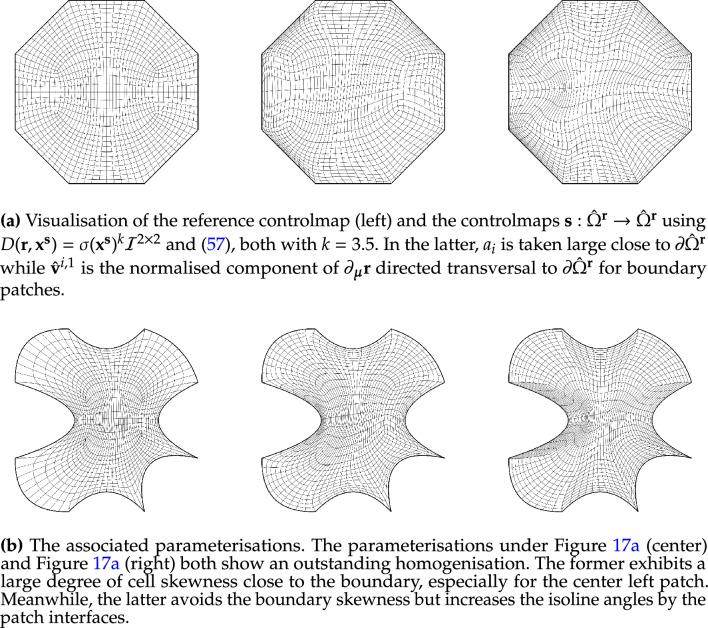
Reparameterisation of a irregular geometry using $D({{\textbf{r}}}, {{\textbf{x}}}^{{{\textbf{s}}}}) = \sigma ({{\textbf{x}}}^{{{\textbf{s}}}})^k {\mathcal {I}}^{2 \times 2}$ (center) and (57) (right). The latter avoids cell skewness close to the boundary

Taking $a_i$ large will force ${{\textbf{s}}}: {{\hat{\Omega }}^{{\textbf{r}}}}\rightarrow {{\hat{\Omega }}^{{\textbf{r}}}}$ to predominantly slide in the direction of ${\textbf{v}}^{i, 1}$ on ${{\textbf{r}}}({\hat{\Omega }}^i)$. For large values of *k*, cell size homogenisation can lead to a considerable degree of cell skewness close to the boundary. As an example, Fig. 17 (center) shows the homogenisation of a geometry with reference parameterisation depicted in Fig. 17 (left) using (57) under $D^{{{\textbf{s}}}}({{\textbf{x}}}^{{{\textbf{s}}}}) = \sigma ^k({{\textbf{x}}}^{{{\textbf{s}}}}) {\mathcal {I}}^{2 \times 2}$ with $k=3.5$. This effect can be avoided by setting $\hat{{\textbf{v}}}^{i, j} = {\widehat{\partial }}_{{\varvec{\mu }}_j} {{\textbf{r}}}$, where $\hat{{\textbf{v}}}^{i, 1} = {\widehat{\partial }}_{{\varvec{\mu }}^{\perp }} {{\textbf{r}}}$ for boundary patches, while taking the $a_i$ large close to the boundary of ${{\hat{\Omega }}^{{\textbf{r}}}}$. The result for $k = 3.5$ is depicted in Fig. 17 (right). With $\nu ^k_{\text {Area}} = 0.515$ for the former and $\nu ^k_{\text {Area}} = 0.518$ for the latter, the homogenisation is only marginally less effective under (57). However, the latter completely avoids cell skewness close to the boundary while sacrificing some regularity across the patch interfaces.

In contrast to method 1., method 2. requires $D^{{{\textbf{s}}}}$ to be a function of ${{\textbf{r}}}$ only and, for convenience, we assume $D^{{{\textbf{s}}}} = {\mathcal {I}}^{2 \times 2}$ such that ${{\textbf{s}}}({{\textbf{r}}}) = {{\textbf{r}}}$. The cell size homogenisation is now encouraged through a proper choice of $D^{{{\textbf{x}}}}({{\textbf{s}}}, {{\textbf{x}}}^{{{\textbf{s}}}})$. Similar to method 1., we take $D^{{{\textbf{x}}}}({{\textbf{s}}}, {{\textbf{x}}}^{{{\textbf{s}}}}) = \omega ({{\textbf{x}}}) {\mathcal {I}}^{2 \times 2}$, for some $\omega ({{\textbf{x}}}) > 0$. Method 2. has the advantage of decoupling the system from (41). This reduces the problem size of the iterative root-finding algorithm, which now computes only ${{\textbf{x}}}^{{{\textbf{s}}}}$ instead of the tuple $({{\textbf{x}}}^{{{\textbf{s}}}}, {{\textbf{s}}})$, with ${{\textbf{s}}}({\varvec{\xi }}) = {{\textbf{r}}}({\varvec{\xi }})$.

This choice of $D^{{{\textbf{x}}}}({{\textbf{s}}}, {{\textbf{x}}}^{{{\textbf{s}}}})$ encourages the contraction of ${{\textbf{r}}}({{\textbf{x}}})$ isolines in ${{\hat{\Omega }}^{{\textbf{r}}}}$ wherever $\omega ({{\textbf{x}}})$ is large and vice-versa. Exchanging the dependency ${{\textbf{r}}}({{\textbf{x}}}) \rightarrow {{\textbf{x}}}({{\textbf{r}}})$, the isolines will now be contracted in regions where $\omega ({{\textbf{x}}})$ is small. Inspired by method 1., we define the family monitor functions

$$\begin{aligned} \omega ({{\textbf{x}}}^{{{\textbf{s}}}})^k({{\textbf{r}}}):= \left( \det \partial _{{\varvec{\mu }}} {{\textbf{x}}}^{{{\textbf{s}}}}({{\textbf{r}}}) \right) ^{-k} \quad \text {on} \quad {{\textbf{r}}}({{\hat{\Omega }}}_i). \end{aligned}$$

**Fig. 18:**
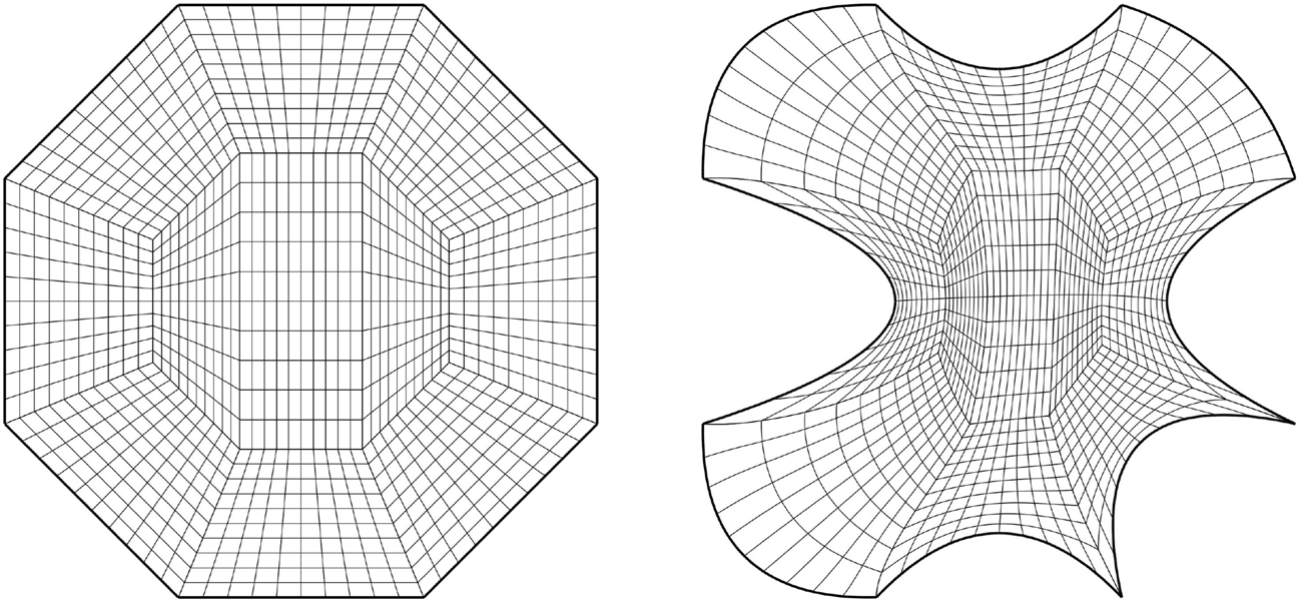
Figure depicting the parameterisations of the tuple $({{\hat{\Omega }}}, {\Omega })$ for $D^{{{\textbf{x}}}} = D^{{{\textbf{s}}}} = {\mathcal {I}}^{2 \times 2}$

**Fig. 19:**
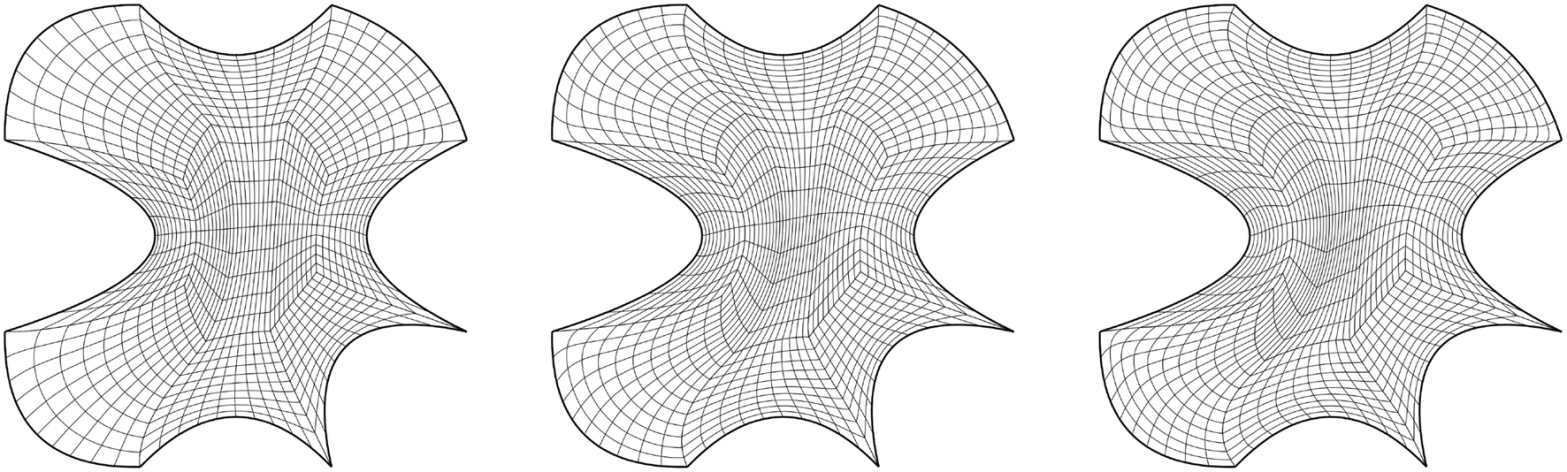
Figure depicting the reparameterisations of the reference parameterisation from Fig. 18 under $\omega ^k({{\textbf{x}}}^{{{\textbf{s}}}})$ for $k=1$, $k=4$ and $k=8$

As such, we are solving the decoupled system with $D^{{{\textbf{x}}}}({{\textbf{x}}}^{{{\textbf{s}}}}) = \omega ^{k}({{\textbf{x}}}^{{{\textbf{s}}}}) {\mathcal {I}}^{2 \times 2}$. Clearly, for a root-finding algorithm to converge, the value of $\det \partial _{{\varvec{\mu }}} {{\textbf{x}}}^{{{\textbf{s}}}}({{\textbf{r}}})$ has to stay positive. As before, the barrier property of (43) prevents intermediate iterates from leaving the set of UNDG maps and the scheme converges reliably for a wide range of $k > 0$ when initialised with the solution of one of the NDF formulations. We are considering the geometry with reference parameterisation from Fig. 18. The same figure shows the bilinearly covered parametric domain. We are monitoring the values of $\nu ^{k}_{\text {Area}}$ and $\nu ^{k}_{\det J}$ (cf. (56)) for $k \in \{0, \ldots , 8\}$. Table 8 contains the associated values, while Fig. 19 depicts the homogenised parameterisations for three different values of *k*. The table clearly demonstrates that the methodology is highly effective at homogenising the cell sizes in the local ${\varvec{\mu }}$ coordinate systems. We also observe a significant reduction in the anisotropy of $\det \partial _{{\varvec{\mu }}} {{\textbf{x}}}_h^{k}$, which is reduced from the initial $\nu _{\det J}^k = 54.7$ to $\nu _{\det J}^k = 6.05$ for $k=8$.

**Table 8:** Table showing the values of $\nu _{\text {Area}}^k$ and $\nu _{\det J}^k$ as defined in (56) after reparameterising the reference parameterisation from Fig. 18 under $\omega ^k({{\textbf{x}}}^{{{\textbf{s}}}})$ for various values of $k \ge 0$

k	0	1	2	3	4	5	6	7	8
$\nu _{\text {Area}}^k$	1	0.716	0.623	0.583	0.558	0.542	0.532	0.524	0.518
$\nu _{\det J}^k$	54.7	23.1	15.6	12.2	10.2	8.87	7.88	6.98	6.05

**Fig. 20:**
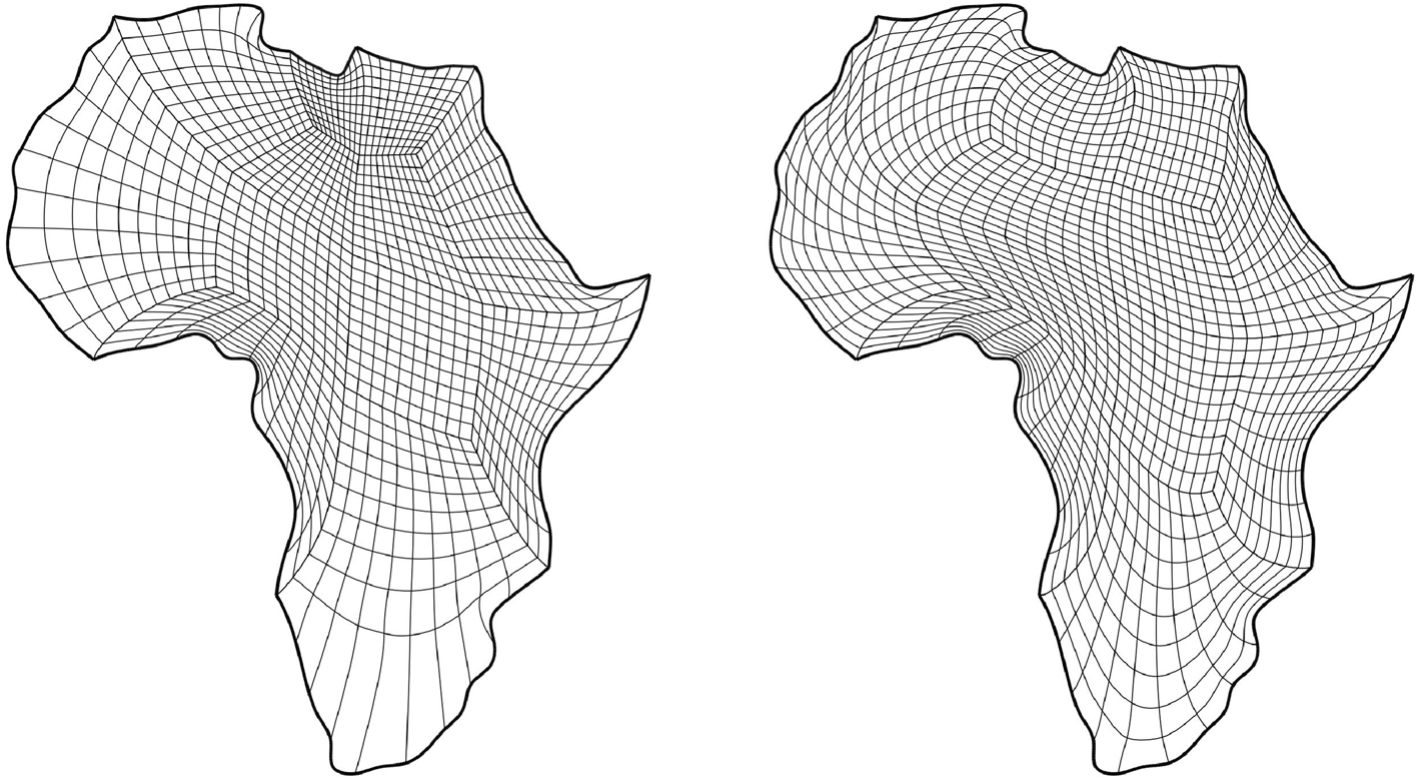
Figure depicting the geometry from Fig. 3 before and after reparameterisation under $\omega ^k({{\textbf{x}}}^{{{\textbf{s}}}})$ with $k=4$. The reparameterisation with $k=4$ significantly improves the cell size homogeneity with respect to the reference solution

Finally, we apply the same methodology to the geometry from Fig. 3. Figure 20 shows the parameterisation before and after reparameterisation with $k = 4$.

### Grid adaptation

In various applications it can be desirable to contract the map’s isolines in regions where a large value of a function or its gradient is assumed. Given a function $f: {\Omega }\rightarrow {\mathbb {R}}^{+}$, with $f \in C^{\infty }({\Omega })$, the clustering of isolines can be achieved by designing diffusivities that contract the map’s cell sizes in regions where $f: {\Omega }\rightarrow {\mathbb {R}}^{+}$ is large (and vice-versa). The most basic choice is $D^{{{\textbf{s}}}}({{\textbf{r}}}, {{\textbf{x}}}) = {\mathcal {I}}^{2 \times 2}$ and $D^{{{\textbf{x}}}}({{\textbf{s}}}, {{\textbf{x}}}) = \sigma ({{\textbf{x}}}) {\mathcal {I}}^{2 \times 2}$. As in Sect. 4.3, this choice decouples (42) and the first equation can be regarded as the Euler-Lagrange equation of

58 $$\begin{aligned} \min \limits _{{{\textbf{r}}}: {\Omega }\rightarrow {{\hat{\Omega }}^{{\textbf{r}}}}} \int \limits _{{\Omega }} \sigma ({{\textbf{x}}}) {\text {tr}} \left( G^{{{\textbf{x}}}\rightarrow {{\textbf{r}}}} \right) \textrm{d} {{\textbf{x}}}\quad \text {s.t.} \quad {{\textbf{r}}}= \left( {\textbf{F}}^{{{\textbf{r}}}\rightarrow {{\textbf{x}}}} \right) ^{-1} \text { on } \partial {\Omega }. \end{aligned}$$

As before, upon exchanging the dependencies ${{\textbf{r}}}({{\textbf{x}}}) \rightarrow {{\textbf{x}}}({{\textbf{r}}})$, the isolines will be contracted in regions where $\varvec{\sigma }({{\textbf{x}}})$ is small. To contract cells in the vicinity of large function values of $f: \Omega \rightarrow {\mathbb {R}}^+$, we design a suitable monitor function $\sigma (\cdot )$. A possible choice is given by [17, Chapter 9]:

$$\begin{aligned} \sigma ({{\textbf{x}}})= & {} \frac{1}{\nu _1 f({{\textbf{x}}})^k + \nu _2}, \quad \text {or} \quad \\ \sigma ({{\textbf{x}}})= & {} \frac{1}{\nu _1 \Vert \nabla f({{\textbf{x}}}) \Vert ^k + \nu _2} \quad \text {for gradient penalisation}. \end{aligned}$$

Here, $\nu _2 > 0$ avoids division by zero in case $f \rightarrow 0$ and the parameters $\nu _1 > 0$ and $k > 0$ tune the degree of penalisation. A numerical scheme is best initialised with the nondegenerate reference solution (i.e., the solution for $\sigma ({{\textbf{x}}}) = 1$).

**Fig. 21:**
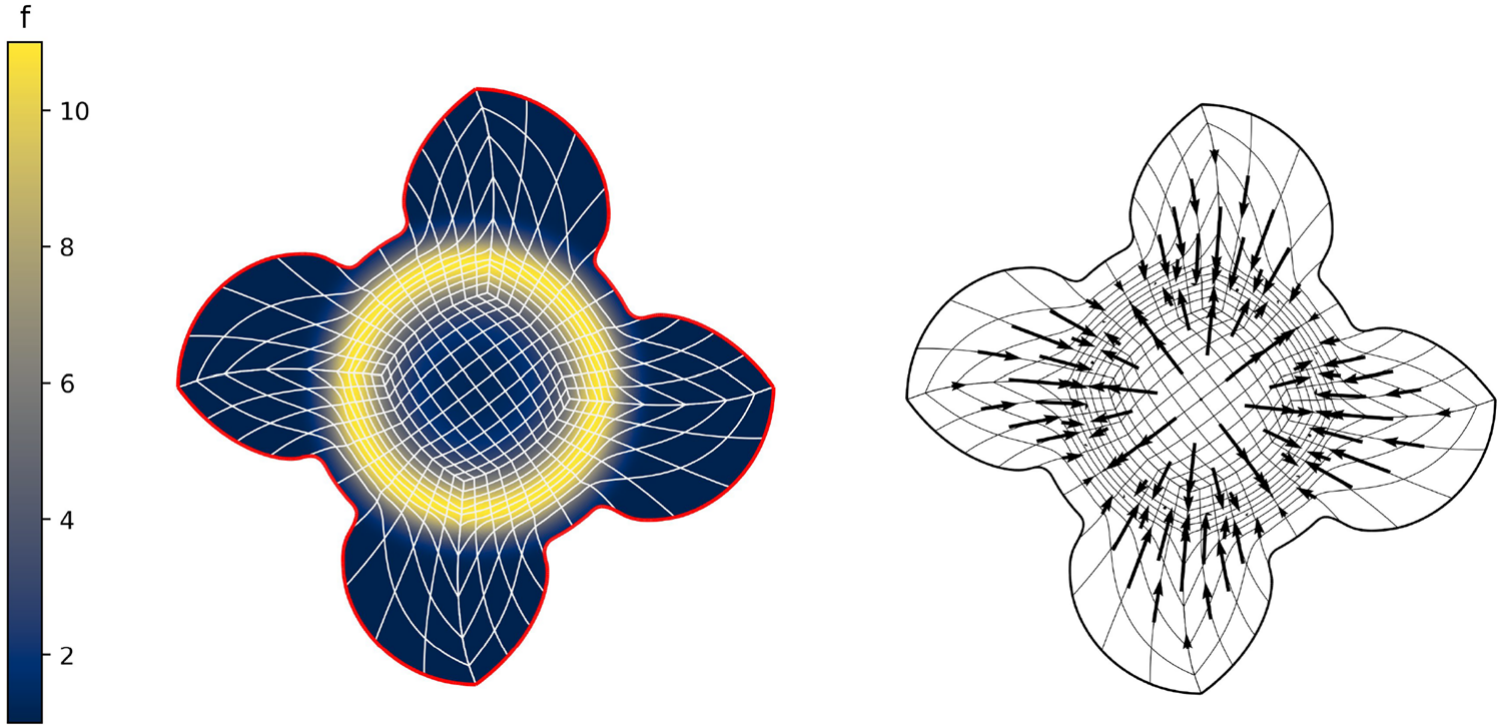
Figure showing the cell size contraction in the vicinity of large function values of a ring-shaped function $f \in C^{\infty }({\Omega })$. The arrows in the right figure show the movement of select points with respect to the reference parameterisation from Fig. 8b

We are considering the screw geometry with reference parameterisation from Fig. 8. Here, we take ${{\textbf{r}}}({\varvec{\xi }}) = {\varvec{\xi }}$ and ${{\hat{\Omega }}^{{\textbf{r}}}}= {{\hat{\Omega }}}$. We would like to contract cells based on the function value of a ring-shaped function $f \in C^{\infty }({\Omega })$ using $\nu _1 = 1$, $\nu _2 = 0.01$ and $k = 1$. Figure 21 depicts the result of reparameterising under $D^{{{\textbf{x}}}}({{\textbf{x}}})$ along with an arrow plot showing the movement of a select number of points with respect to the reference map.

The figure shows a strong contraction of cells in the vicinity of large function values, clearly demonstrating that the methodology has the desired effect. The cell contraction can be increased by increasing the value of $\nu _1$ or *k*. However, strong penalisation can have unpredictable effects on the cells, in particular close to the patch vertices.

**Fig. 22:**
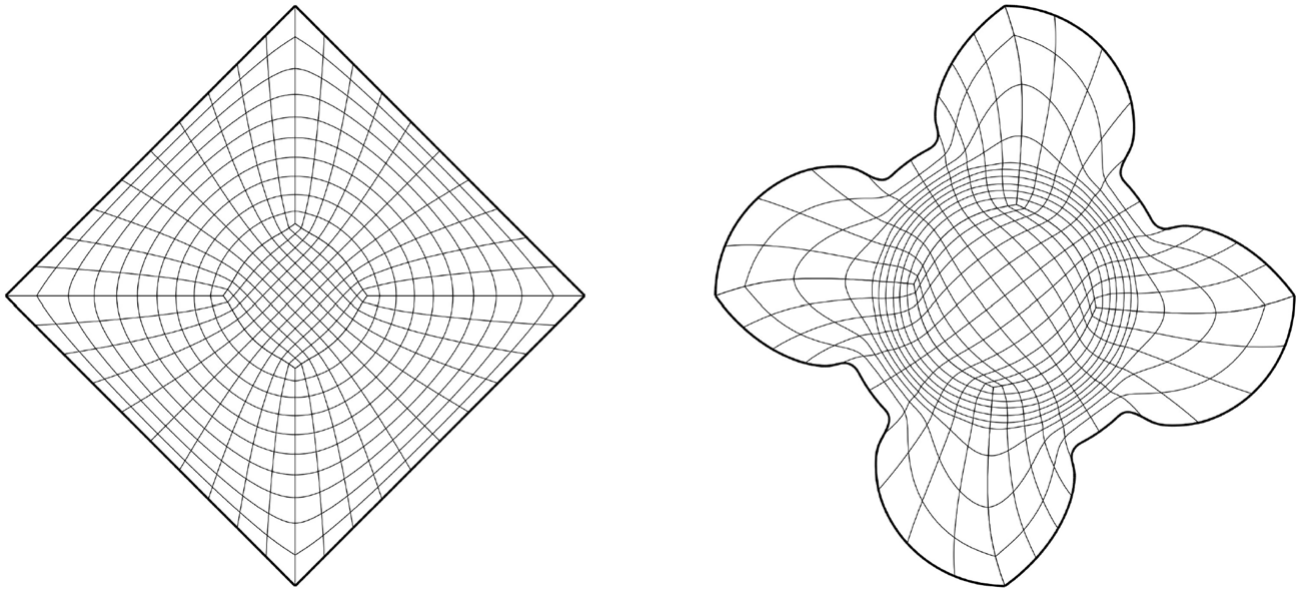
Figure showing the parameterisation of ${{\hat{\Omega }}^{{\textbf{r}}}}= {{\hat{\Omega }}}$ and the result of performing cell size contraction under the coordinate transformation

In practice, this can be avoided by performing patch interface removal using the techniques of Sect. 4.2. We are considering the same example as before while now performing interface removal with stabilisation in ${{\hat{\Omega }}^{{\textbf{r}}}}$. Figure 22 shows the images of locally drawn isolines in ${{\hat{\Omega }}^{{\textbf{r}}}}$ along with the result of performing cell contraction using the same parameters. Compared to Fig. 21, isolines crossing the patch interfaces exhibit less erratic behaviour and align well with the ring-shaped function.

**Fig. 23:**
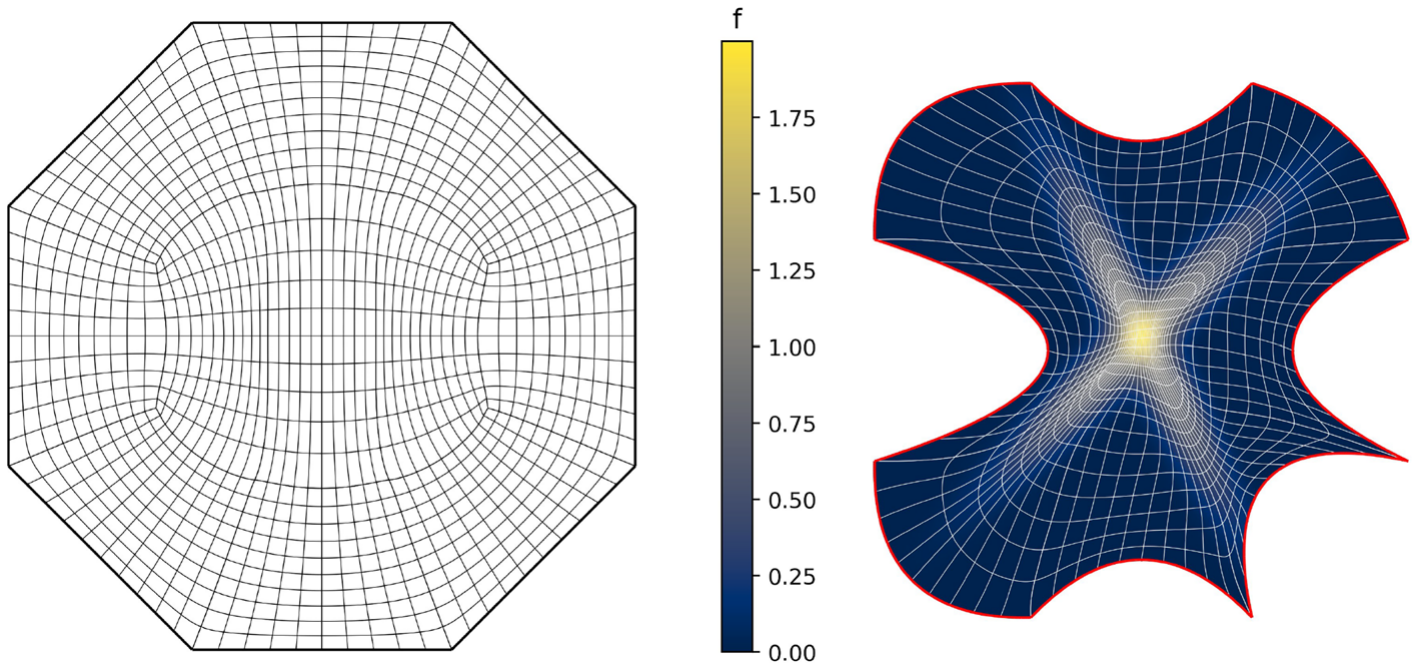
A further example of grid adaptation in the vicinity of large function values with interface removal

To demonstrate that the proposed technique (in combination with interface removal) is effective when applied to geometries with fewer symmetries than in Fig. 21, we refer to Fig. 23.

## Conclusion

We have presented a PDE-based parameterisation framework for planar multipatch domains based on the concept of harmonic maps. For this, we presented a total of four different numerical approaches capable of computing valid parameterisations for a wide range of piecewise smooth Lipschitz domains bounded by a collection of spline curves. We presented three different algorithms in nondivergence form, two of which are in mixed form and one based on $C^0$-DG. Furthermore, we presented one approach based on the inverse harmonicity requirement’s weak form. We concluded that the NDF-discretisations in mixed form performed similarly well in the essayed benchmark problems while consistently exhibiting slightly better convergence rates than the $C^0$-DG approach. On the other hand, we concluded that the $C^0$-DG approach is the computationally least expensive approach that can be initialised with a degenerate initial iterate. The experiments demonstrate that the weak form discretisation converges reliably when initialised with the solution of one of the NDF-discretisations while performing only marginally worse than or on par with Winslow’s original approach. Since the $C^0$-DG approach’s solution is usually sufficiently close to the discrete root, we concluded that a combination with the weak form discretisation constitutes a computationally feasible and effective means to compute a uniformly nondegenerate map for the geometries considered in this work. Hereby, the combination of the two approaches substantially reduces the need for a posteriori refinement in case the NDF-solution is degenerate, thanks to the weak form’s barrier property.

In Sect. 4 we augmented the numerical schemes from Sect. 3 with a general mechanism capable of controlling the parametric properties of the computed maps. For this, we introduced two differing techniques: (1) introducing a curvilinear coordinate system in the parametric domain and (2) using more general elliptic PDEs for the purpose of finding a surface parameterisation. The framework’s utility was demonstrated in three use cases: (1) patch interface removal (2) cell size homogenisation and (3) grid adaptation.

Utilising an only essentially bounded diffusivity for the purpose of inducing a coordinate transformation via a controlmap, while enabling many novel ways of controlling the outcome, is associated with a number of potential robustness bottlenecks, such as the possibility of creating singularities in the interior of the domain. Here, we proposed a stabilisation via Gaussian blending functions on the quadrangulation’s vertices. However, a more thorough investigation of the effect of the controlmap’s reduced regularity on the computed maps constitutes a topic for future research. Furthermore, given that the parametric domain is typically given by a convex polygon, we see great potential in the use of computationally inexpensive algebraic methods to create a controlmap that builds desired features into the harmonic map. Providing additional techniques for parametric control, based on the framework introduced in Sect. 4, will be the topic of a forthcoming publication.

## Data Availability

Not applicable.
